# Supplementary material for: Multivalent supramolecular fluorescent probes for accurate disease imaging
Source: Sci Adv. 2024 Oct 18;10(42):eadp8719. doi: 10.1126/sciadv.adp8719 (PMC11488570; doi:10.1126/sciadv.adp8719)
Supplement: 20241018-1 [file sciadv.adp8719.v1.pdf]

OPTICS

Multivalent supramolecular fluorescent probes for accurate disease imaging

Qian Wu†, Zhixuan Zhou†, Li Xu, Haichen Zhong, Bin Xiong, Tianbing Ren, Zhe Li, Lin Yuan*, Xiao-Bing Zhang*

Copyright © 2024 The Authors, some rights reserved; exclusive licensee American Association for the Advancement of Science. No claim to original U.S. Government Works. Distributed under a Creative Commons Attribution License 4.0 (CC BY).

Optical imaging is a powerful tool for early disease detection and effective treatment planning, but its accuracy is often compromised by the uptake of imaging materials by the mononuclear phagocyte system (MPS). Herein, we leverage multivalent host-guest interactions between cyanine dyes and β-cyclodextrin polymers to develop supramolecular probes with enhanced stability, optical, and transport profiles for accurate in vivo imaging. These multivalent interactions not only ensure the stability of the probes but also enhance fluorescence efficiency by minimizing nonradiative decay. Our self-assembly approach effectively modulates probe size and surface properties, enabling evasion of MPS clearance and promoting prolonged bloodstream circulation, thereby improving the signal-to-background ratio for imaging. The effectiveness of our design is demonstrated by substantial advancements in the early diagnosis of acute kidney injury and by providing high-contrast imaging and precise surgical navigation across various tumor models. Our strategy not only advances optical imaging materials toward clinical translation but also establishes a versatile platform applicable to multiple imaging modalities.

INTRODUCTION

The quest for accurate imaging with high signal-to-background ratio (SBR) is crucial in the early diagnosis, treatment, and prevention of numerous diseases that substantially affect human health (*1*). Optical imaging stands out among various modalities for its noninvasive, high-resolution, and real-time imaging capabilities, which are essential in clinical settings (*2*, *3*). Nevertheless, a notable challenge arises with the rapid uptake of imaging materials by the mononuclear phagocyte system (MPS) following intravenous administration, leading to their undesired accumulation in nontarget tissues (*4*–*6*). This rapid systemic clearance, coupled with nonspecific interactions with serum proteins, reduces the circulation half-life and thus hampers their efficient delivery to lesions, even when molecular probes are modified with targeting ligands, hindering the accurate and high-contrast imaging of diseases (*7*–*9*). Therefore, developing strategies to circumvent MPS capture, thereby enhancing the accumulation of molecular probes at the desired lesion sites, is pivotal for realizing the full clinical potential of optical imaging probes.

Current strategies for mitigating MPS capture typically involve conjugating hydrophilic moieties, such as sulfonates, polyethylene glycol, peptides, and macrocycles, to molecular probes (*10*–*14*). While this approach increases the renal clearance rate of the probes, it concurrently shortens their circulation half-life, thus restricting their use primarily to renal disease imaging (*13*). There is a pressing need for a versatile and effective probe design that circumvents MPS capture without accelerating clearance, thereby extending the circulation time of the probe, which is crucial for achieving accurate imaging with high SBRs across a variety of lesions.

Molecular self-assembly recently emerged as a versatile and effective strategy for developing materials with desired properties in optical, catalytic, and biomedical applications, effectively bypassing complex synthesis and purification processes (*15*–*18*). With its ability to finely regulate the nanoscale morphology, colloidal size, and surface chemistry of materials (*15*–*18*), which are critical for interactions with macrophages and subsequent phagocytosis (*19*, *20*), molecular self-assembly is positioned as a promising technique for enhancing the in vivo transport of materials. In addition, fabricating supramolecular materials through the self-assembly of hydrophobic fluorophores and complementary hydrophilic precursors not only improves the compatibility of the fluorophores with aqueous environments but also substantially reduces nonradiative energy decay, thereby enhancing fluorescence efficiency (*21*, *22*). Consequently, the design of supramolecular probes may offer a synergistic combination of optimal in vivo transport and superior optical properties, facilitating accurate imaging with high SBRs. However, translating these advancements into clinical settings remains a challenge, largely attributed to the dynamic and reversible nature of the self-assembly processes when interfaced with biological systems (*23*).

Herein, we report the development of a series of supramolecular fluorescent probes through the multivalent molecular self-assembly of cyanine dyes and β-cyclodextrin polymers (CDP) (Fig. 1). Inspired by the principle of multivalency, a hallmark of nature's strategy for high-affinity molecular interactions (*24*), we incorporated multiple hydrophobic moieties into the dye structures. This design fosters robust multivalent host-guest interactions with CDP, resulting in supramolecular probes with exceptional in vivo stability. These probes evade MPS clearance and demonstrate minimal interactions with serum proteins, attributed to the surface hydroxyl groups of the cyclodextrins, which reduce positive surface charge and enhance surface polarity. In addition, the encapsulation of the dyes within the cyclodextrins leads to pronounced fluorescence in aqueous environments, due to the effective prevention of aggregation and shielding of the dye molecules from the external environment. The combined in vivo stability, superior optical properties, and favorable transport profile of our supramolecular probes enable accurate imaging across a variety of diseases with elevated SBRs. The effectiveness of this approach is underscored by its application in the early detection of drug-induced acute kidney injury (AKI) and in providing precise imaging and surgical guidance in subcutaneous and orthotopic tumor

State Key Laboratory for Chemo/Bio-Sensing and Chemometrics, College of Chemistry and Chemical Engineering, Hunan University, Changsha 410082, China.
*Corresponding author. Email: lyuan@hnu.edu.cn (L.Y.); xbzhang@hnu.edu.cn (X.-B.Z.)
†These authors contributed equally to this work.

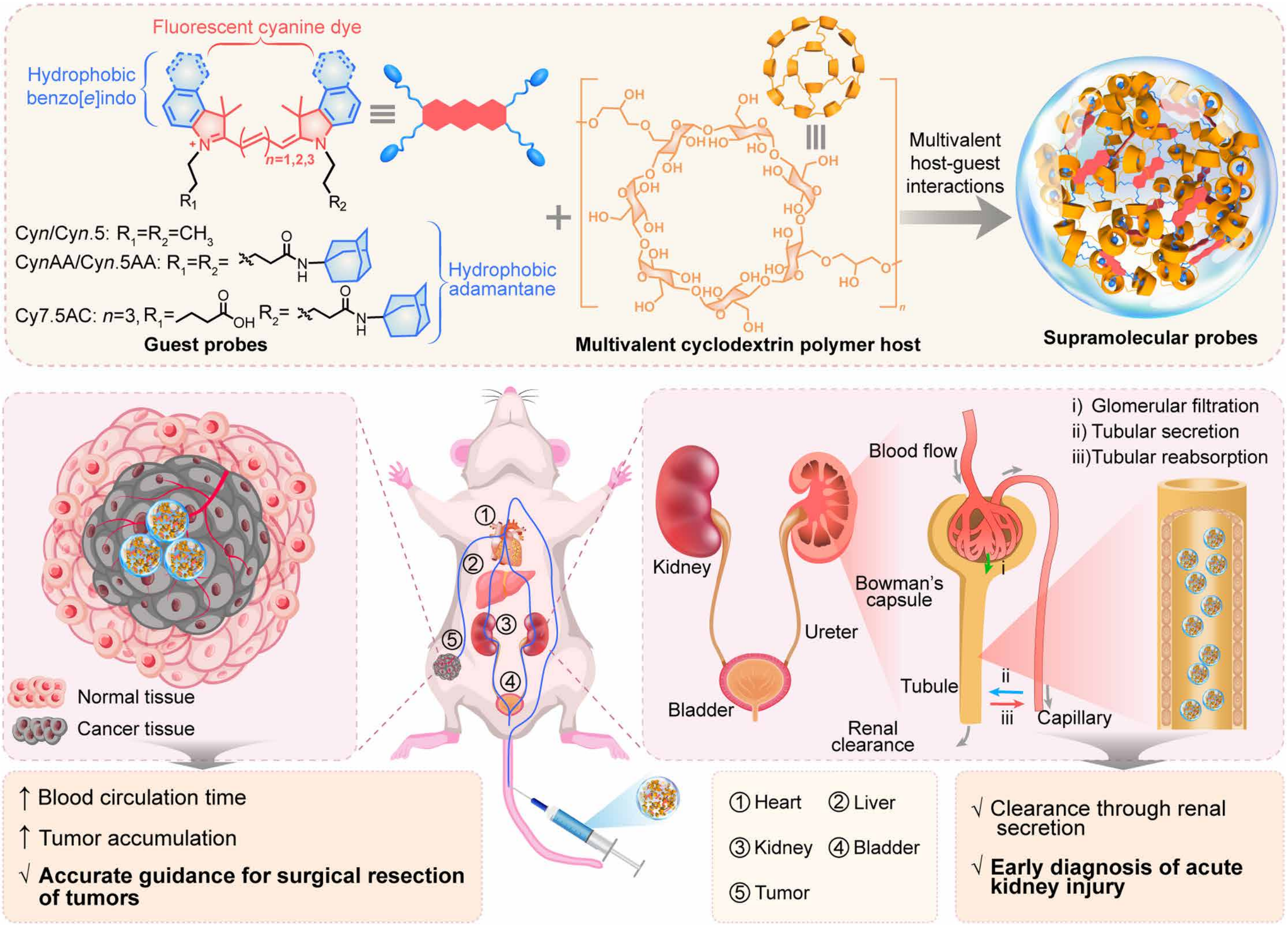

Fig. 1. Design and working mechanism of the supramolecular probes formed through multivalent self-assembly between β-cyclodextrin polymers and cyanine dyes. These host-guest interactions, marked by its multivalency, yields fluorescent supramolecular probes with a specific nanosize and strong in vivo stability. Incorporating cyclodextrin enhances the optical properties of the supramolecular probes in aqueous environments, further reducing MPS capture and minimizing its interaction with serum proteins. These attributes lead to prolonged blood circulation and enhanced tumor accumulation. Furthermore, the supramolecular probes undergo clearance through the renal secretion pathway. Such capabilities bolster both the early diagnosis of AKI and potent tumor imaging, providing precise guidance for tumor excision.

models. This versatile supramolecular platform not only lays the groundwork for enhanced applications in optical imaging diagnostics but also opens avenues for integration with clinical modalities like magnetic resonance imaging (MRI), x-ray computed tomography (CT), and positron emission tomography (PET). This expansion promises to substantially elevate clinical diagnostics, enabling more accurate disease characterization and tailored treatment strategies, thereby improving treatment outcomes across various medical disciplines.

RESULTS

Design and optimization of the supramolecular probes

Cyanine dyes were chosen for our study due to their narrow absorption half-peak widths, large molar extinction coefficients, and tunable absorption and emission profiles, characteristics that have been highlighted for their broad applications in bioimaging (*25*, *26*). To address stability concerns in biological systems, our probe design incorporates multivalency, a widespread and effective mechanism in nature that facilitates strong and selective interactions through multiple binding sites. This is particularly advantageous when univalent interactions prove insufficient. Following this design principle to ensure structural integrity and functional efficacy of our supramolecular probes in complex biological systems, hydrophobic indo or benzo[*e*]indo moieties were integrated as the terminal groups of the symmetric polymethine skeleton, which resulted in guest fluorophores denoted as Cy*n* and Cy*n*.5, where $n = 3$, 5, and 7. Subsequently, adamantane groups were introduced via the nitrogen atoms inherent in the dye structures, yielding symmetric derivatives Cy*n*AA and Cy*n*.5AA. Asymmetric Cy7.5 AC bearing one adamantane and one carboxyl group was also prepared. CDP was used for the fabrication of the supramolecular probes, given its ease of acquisition and propensity to enhance host-guest interaction strength via multivalency (Fig. 1).

The apparent association constant (K_a) between the designed cyanine dyes and CDP was determined through fluorescent titration experiments (*27*). As both the number of hydrophobic binding sites

and the length of the polymethine skeleton in the guest fluorophores expanded, the K_a for the host-guest interactions showed a pronounced increase. This increase spans from 10^5 to values exceeding 10^7 M^{-1} (Fig. 2A, fig. S1, and table S1), indicating affinities that not only are strong but, in some cases, also exceed those of many natural protein-ligand interactions (*28*, *29*). In addition, fluorescent titration experiments and isothermal titration calorimetry (ITC) analyses suggest that the interactions involving the univalent (2-hydroxypropyl)-β-cyclodextrin (HPβCD) and cyanine dyes were markedly reduced relative to those with the multivalent CDP (figs. S2 and S3 and table S2). These observations highlight the effectiveness of multivalent interactions in fabricating robust supramolecular systems, thereby facilitating the development of supramolecular nanomaterials that have adequate stability for in vivo applications. Further titration experiments on the supramolecular system with the highest K_a, Cy7.5AA@CDP, determined that the optimal concentration is 10 mM for CDP (fig. S6). The average number of Cy7.5AA dye molecules binding to each CDP in the synthesized Cy7.5AA@CDP was estimated to be 3.45 (fig. S7), closely matching the theoretical estimate of 3.24, calculated based on sample preparation parameters. While this value might seem modest, it not only confirms the precision and effectiveness of our synthetic methodology but is also crucial for ensuring the stability and functional integrity of the supramolecular probes in vivo, where adaptation to the complex and dynamic nature of biological environments is essential.

Dynamic light scattering (DLS) analysis revealed that the multivalent interactions between Cy7.5AA and CDP lead to the formation of a nanosized material with an average hydrodynamic diameter (D_h) of 459 nm (Fig. 2B) and a slightly positive zeta potential of 1.2 mV in phosphate-buffered saline (PBS) (Fig. 2C). In contrast, because of limited solubility, the small molecule precursor Cy7.5AA forms nanoaggregates with broad size distribution, with an average D_h of 531 nm and large aggregates of more than 1000 nm, and a considerably stronger positive zeta potential of 14.1 mV. An analog of the guest cyanine dyes, the US Food and Drug Administration (FDA)–approved indocyanine green (ICG), exhibited a D_h of 1.3 nm due to its good solubility in water endowed by its sulfonic acid groups (fig. S8). Scanning electron microscopy (SEM) images highlighted Cy7.5AA@CDP as spherical nanoparticles with approximate diameters of 323 ± 29 nm (fig. S9), while Cy7.5AA appeared as an amorphous aggregate (fig. S10). These observations were further corroborated by transmission electron microscopy (TEM) analysis (Fig. 2D and fig. S11). These observations suggested that multivalent host-guest interactions facilitate the formation of nanoparticles, endowing them defined colloidal sizes and enhanced compatibility with water.

The supramolecular probes presented well-defined absorption and emission spectra in PBS (Fig. 2, E and F), reminiscent of ICG in an organic phase (EtOH) (Fig. 2, E and F). Conversely, analogous small molecular cyanine dyes displayed pronounced aggregation in PBS, coupled with marked fluorescence quenching (figs. S12 and S13). The absolute fluorescence quantum yield of Cy7.5AA@CDP was found to be 15.5% in PBS, a notable improvement over ICG (2.0%) (table S3). Cy7.5AA@CDP exhibited good stability within the pH range of 4 to 11 (fig. S14), which is broader than the pH range encountered in in vivo biological systems that typically vary from slightly acidic to neutral and can occasionally extend to more acidic or alkaline conditions in specific microenvironments such as lysosomes or certain diseased tissues, suggesting the robustness of the supramolecular probe across various physiological conditions. Furthermore, self-assembly also improved the chemical and photostability of the fluorescence dye (Fig. 2G and figs. S15 to S28) and reduced nonspecific interactions with serum proteins (Fig. 2H and fig. S29). Collectively, these findings underscore that multivalent host-guest interactions endow supramolecular probes with commendable biostability and optical attributes, making them suitable for in vivo imaging.

In vivo transport and stability

Inspired by the defined nanoscale dimensions and favorable optical properties of the supramolecular probes, we explored their potential for in vivo applications. The biocompatibility of the probes was examined, revealing minimal cytotoxicity (figs. S30 to S32) and negligible tissue damage after intravenous injection (figs. S33 and S35). Furthermore, biochemistry and hematology analyses were conducted on healthy mice treated with Cy7.5AA@CDP up to 72 hours after injection (fig. S34), with all indicators remaining in the normal range, suggesting negligible biological toxicity of the supramolecular probes.

The in vivo transport profiles of the supramolecular probes were then investigated. Real-time in vivo imaging of probes using different hosts (CDP and HPβCD) and guest fluorophores (Cy7.5/Cy7AA/Cy7.5 AC/Cy7.5AA) was performed. Fluorescence signals were observed in both the kidneys and liver of the mice injected with any of these supramolecular probes (Fig. 2, I to K, and fig. S36). Mice injected with Cy7.5AA@CDP showed the most pronounced fluorescence in the kidneys, whereas weaker signals were detected in those given complexes with lower K_a. The accumulation of the supramolecular probes in the kidney is further supported by fluorescence images of mice organs post-injection of the probes (figs. S44 and S50). When comparing the in vivo imaging efficacy of the host-guest complexes, it became evident that strong multivalent interactions originating from both host and guest molecules are essential for good stability and shielding of the dye molecule from the aqueous environments (fig. S37) to obtain optimal imaging results of supramolecular probes in vivo.

The concentrations of either ICG or Cy7.5AA@CDP in the blood were quantified by collecting fluorescence signals post-intravenous injection via fluorescence imaging (Fig. 3, A and B) as well as high-performance liquid chromatography (HPLC) analysis (Fig. 3C). In both studies, Cy7.5AA@CDP exhibited an elimination half-life exceeding 210 min, with its concentration remaining above 30% of the injected dose (ID) even 300 min after injection. On the other hand, the concentration of ICG detected in the blood was 16% ID at 5 min after injection (Fig. 3B). The extended circulation time of the supramolecular probe was facilitated by its almost neutral surface charge and surface hydroxyl groups, which improved polarity and hydrophilicity, minimizing nonspecific protein interactions and uptake by the MPS in living organisms (*12*, *30*–*32*). This is further supported by the minimal cellular uptake of the supramolecular probe Cy5AA@CDP by hepatocyte cell line AML12 and macrophage cell line RAW264.7 compared to the corresponding small molecule probes Cy5 and Cy5AA (fig. S38). The clearance pathways of the probes were investigated using whole-body longitudinal fluorescence imaging (Fig. 3D). Five minutes after ICG was injected into mice, a strong fluorescent signal was detected in the liver, with the intensity increasing steadily over the course of 50 min (Fig. 3E). In contrast, mice treated with Cy7.5AA@CDP exhibited bright fluorescence signals in the kidneys (Fig. 3F). The fluorescence intensity ratios of Cy7.5AA@CDP to ICG in the kidneys and liver, 100 min after injection, were measured to be 4.01 ± 0.67 and 0.33 ± 0.02, respectively (Fig. 3G). These

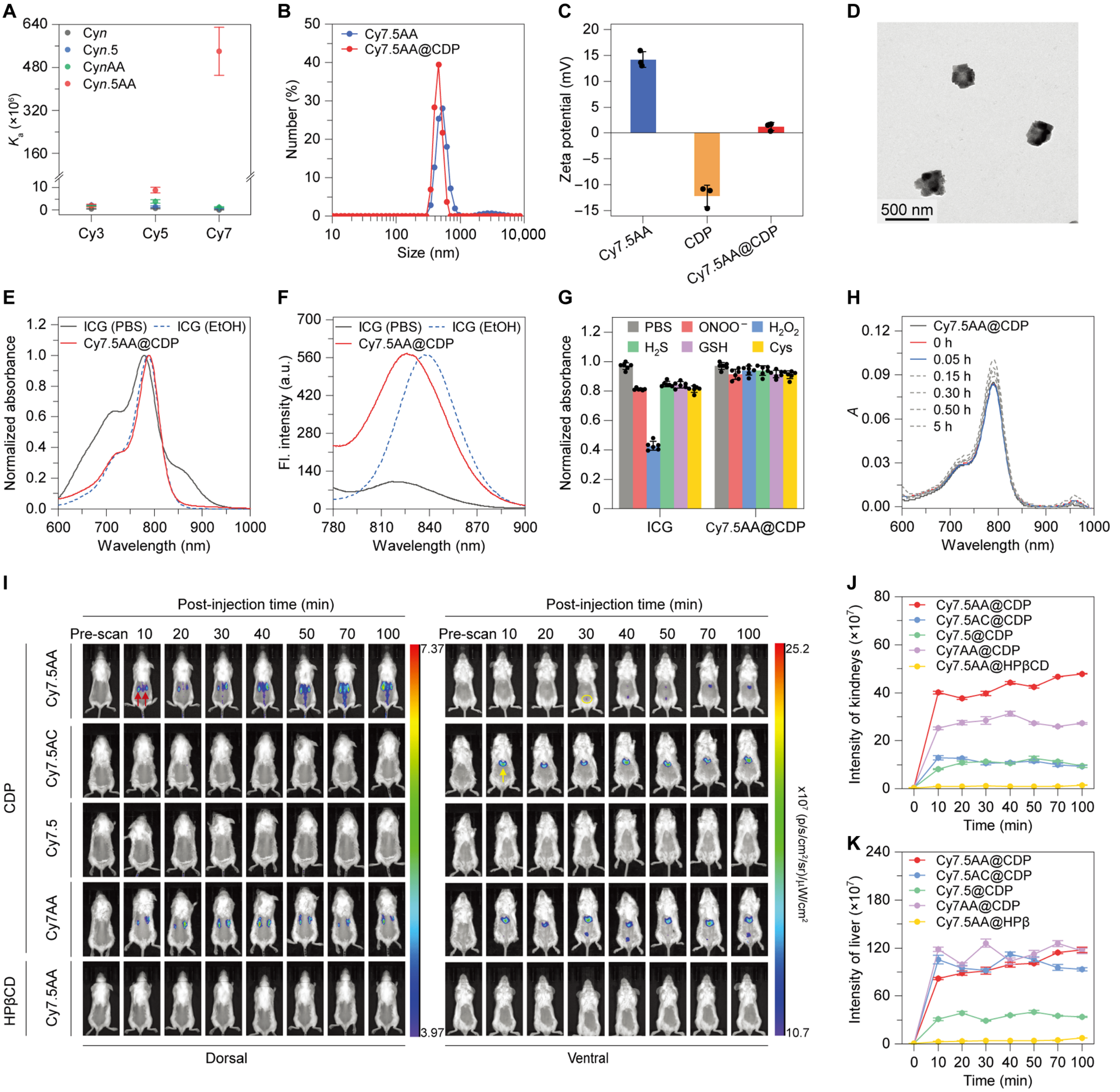

Fig. 2. Self-assembly, optical properties, and in vivo biodistribution of the supramolecular probes. (**A**) Apparent association constants (K_a) between the designed guest cyanine dyes and β-cyclodextrin polymers (CDP), determined through fluorescent titration experiments. (**B**) Hydrodynamic diameters of Cy7.5AA and Cy7.5AA@CDP in PBS determined by DLS analysis. (**C**) Zeta potential values for Cy7.5AA, CDP, and their assembly Cy7.5AA@CDP. (**D**) Representative TEM image of Cy7.5AA@CDP. (**E**) Normalized absorption spectra of ICG (10 μM, in PBS or EtOH) and Cy7.5AA@CDP (10 μM in PBS). (**F**) Fluorescence (Fl.) spectra of ICG (10 μM, in PBS or EtOH) and Cy7.5AA@CDP (10 μM in PBS). a.u., arbitrary units. (**G**) Relative absorbance for ICG (778 nm) and Cy7.5AA@CDP (788 nm) in PBS upon addition of agents such as $ONOO^-$ (5 μM), H_2O_2 (100 μM), H_2S (100 μM), glutathione (GSH) (1 mM), and Cys (100 μM). Cy7.5AA@CDP demonstrated superior stability with minimal changes, in contrast to ICG. (**H**) Time-dependent absorption spectra Cy7.5AA@CDP (1 μM) in PBS with HSA (50 μM) at 37°C. A consistent absorption profile with no peak shifts suggested minimal interaction between the supramolecular probe and HSA. (**I**) Fluorescence images of live mice post-intravenous administration of various supramolecular probes (100 μM, 100 μl) at different intervals. Red and yellow arrows indicate the kidneys and liver on the dorsal and ventral sides, respectively, while yellow circles indicate the bladder. (**J** and **K**) Post-injection fluorescence intensities over time in the kidneys (J) and liver (K) of mice administered with different supramolecular probes. Cy7.5AA@CDP, having the highest K_a, displayed the brightest kidney fluorescence. Conversely, weaker kidney signals and enhanced liver signals were noted for complexes with diminished binding strengths. The data are the means ± SD. $n = 3$ independent experiments.

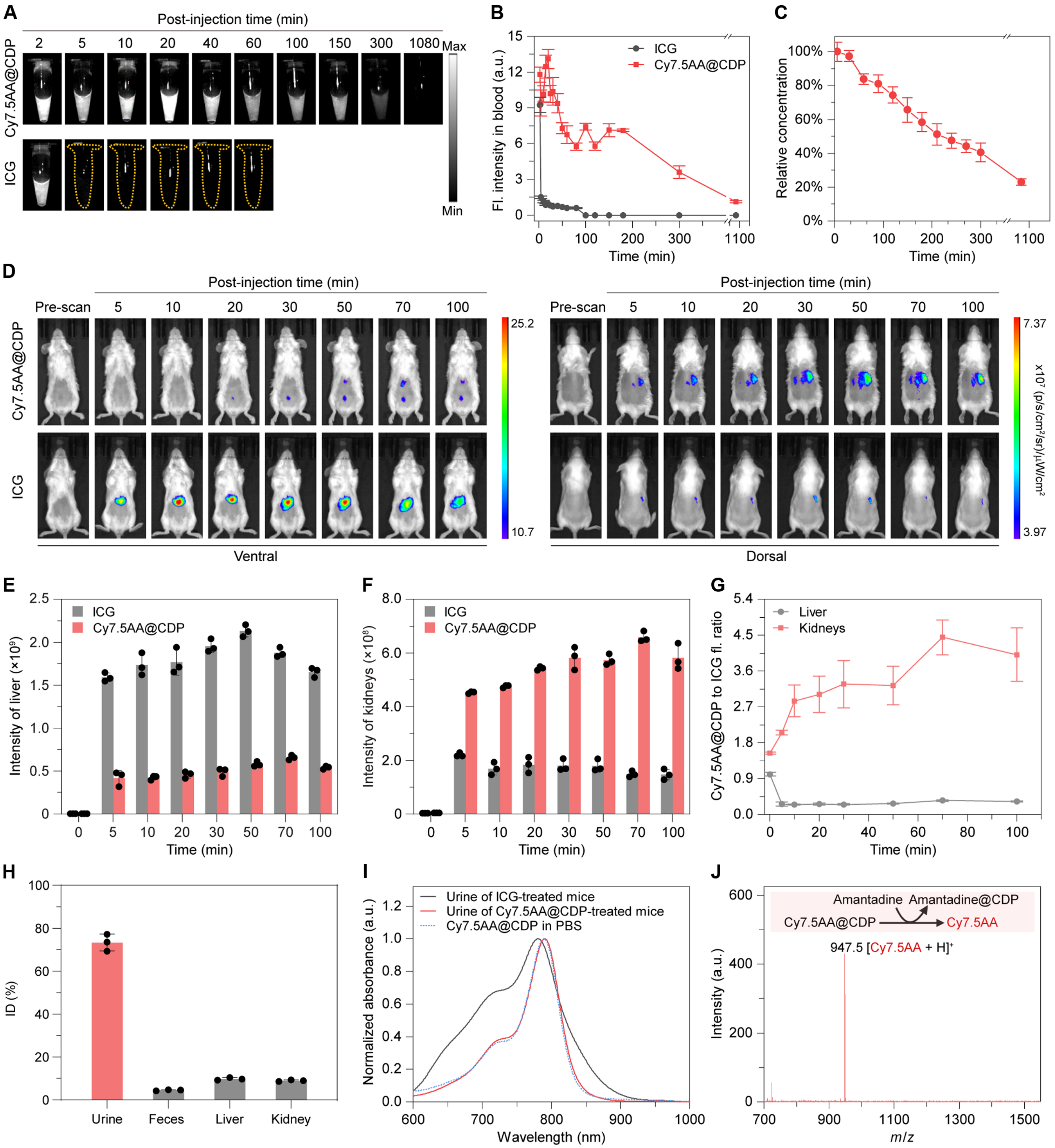

Fig. 3. In vivo clearance pathways of the supramolecular probes after intravenous injection. (**A** and **B**) Fluorescence images (A) and corresponding intensity (B) in blood samples from mice post-administration of either ICG or Cy7.5AA@CDP at various time intervals. (**C**) Relative concentrations of Cy7.5AA@CDP in blood samples from mice at various time intervals post-administration. (**D**) Representative fluorescence images of mice following treatment with ICG or Cy7.5AA@CDP, captured over different durations post-injection. (**E** and **F**) Fluorescence intensities of liver (E) and kidneys (F) in mice after injections of ICG or Cy7.5AA@CDP, tracked over different time intervals. (**G**) Fluorescence intensity ratio of Cy7.5AA@CDP to ICG in both kidneys and liver from mice at different post-injection timeframes. High kidney values and low liver values indicate a shift in clearance routes from hepatic to renal for the supramolecular probe. (**H**) Renal clearance study of Cy7.5AA@CDP using HPLC 24 hours after injection. Other organs were also examined but showed concentrations too low for quantification. (**I**) Normalized absorption spectra of urine samples of mice treated with ICG or Cy7.5AA@CDP and Cy7.5AA@CDP in PBS as a reference. (**J**) MALDI-TOF mass spectrum from urine samples of Cy7.5AA@CDP-treated mice after addition of amantadine (5 mM). This addition led to the release of the free Cy7.5AA molecule from the complex Cy7.5AA@CDP, rendering a discernible peak for Cy7.5AA in the spectrum, suggesting the optimal in vivo stability of Cy7.5AA@CDP during both circulation and clearance phases. The data are the means ± SD. $n = 3$ independent mice.

observations suggest that CDP encapsulation of the small molecular probes primarily shifts the clearance pathway from hepatic clearance to renal clearance.

Urinalysis was then conducted to quantify the differences in clearance pathways between the probe and the small molecule probe. The renal clearance efficiency of Cy7.5AA@CDP 24 hours after injection was determined to be 76.4 ± 9.3% ID, considerably higher than the efficiency of ICG at 1.8 ± 0.6% ID (figs. S39 and S40 and tables S4 and S5). This discrepancy is likely due to ICG's strong binding to serum proteins (fig. S29A) and its rapid transfer to the liver post-intravenous administration (*11*, *33*), resulting in minimal renal accumulation and an almost negligible urine signal. Comprehensive analysis of the urine, feces, and major organs 24 hours after injection using HPLC further supports that most (>70% ID) of the Cy7.5AA@CDP were renally cleared, 5% ID underwent hepatic clearance, and both the liver and kidney showed ~9% residual presence of the probe (Fig. 3H). These findings are supported by second near-infared (NIR-II) fluorescence images of the major organs of mice taken 24 hours after injection of Cy7.5AA@CDP, along with urine and feces collected over the entire period (fig. S42). Strong fluorescence signals were observed in the urine, while weak signals were detected in the liver, kidney, and feces.

The in vivo stability of Cy7.5AA@CDP was confirmed by the detection of its characteristic absorption peak in the urine of mice, consistent with that in PBS (Fig. 3I). Furthermore, mass spectrum signals corresponding to the free Cy7.5AA molecule were only detected when excess amantadine was added to the urine of mice treated with Cy7.5AA@CDP, leading to the release of free Cy7.5AA molecules from the complex (Fig. 3J) (*34*). This observation indicates that complex Cy7.5AA@CDP remained remarkably stable even after 24 hours of circulation and subsequent clearance.

Clearance of the supramolecular probe through renal secretion

Renal clearance encompasses three pathways: glomerular filtration, tubular secretion, and tubular reabsorption (Fig. 4A) (*35*). Considering the mesoscale colloidal sizes of our supramolecular probes, which exceed the glomerular filtration threshold (*36*), they likely passively localize within the renal proximal tubular epithelium during circulation, facilitating renal clearance through tubular mechanisms (*37*, *38*). To examine the renal clearance pathway of the supramolecular probes, mice were treated with cimetidine or probenecid, which selectively inhibit renal tubular secretion and reabsorption (*39*). All mice were then intravenously injected with Cy7.5AA@CDP, and the whole-body longitudinal fluorescence imaging was conducted (Fig. 4B and fig. S43). At 15 min after injection, mice that had received either inhibitor displayed pronounced fluorescent signals in their kidneys (Fig. 4C). This intensity consistently increased with the extension of imaging time. In comparison, saline-treated mice showed an initial increase in the kidney signal that plateaued between 60 and 90 min after injection and then began to gradually decline. Notably, an intense signal was detected in the bladders of saline-treated mice as early as 5 min after injection (Fig. 4D). This bladder signal was markedly diminished in the inhibitor-treated groups, indicating that the clearance of Cy7.5AA@CDP was predominantly via tubular secretion and reabsorption pathways. The ratios of kidney-to-bladder intensity in saline-treated mice were 0.56 ± 0.04 at 5 min and 0.63 ± 0.02 at 120 min after injection (Fig. 4E). This data clearly suggests the excretion of Cy7.5AA@CDP. In comparison, the ratios in cimetidine-treated mice were 1.61 ± 0.04 and 1.84 ± 0.10 at the aforementioned time intervals and 1.93 ± 0.22 and 2.78 ± 0.81 in probenecid-treated mice, implying a markedly slower clearance rate for Cy7.5AA@CDP in the presence of the inhibitors.

Supramolecular probe Cy5AA@CDP was used to investigate the clearance mechanism. Cy5AA@CDP was selected due to its similar in vivo transport and distribution profiles compared to Cy7.5AA@CDP (fig. S44), as well as its high fluorescence efficiency and brightness in the visible light region (table S3), matching the setups of commercial fluorescence microscopes. The supramolecular probe was introduced intravenously into mice, with kidneys dissected 10 min following the injection. Fluorescence images of the entire kidney sections revealed a widespread signal corresponding to the supramolecular probe throughout both the renal cortex and medulla (Fig. 4F). Detailed images of the renal cortex showcased intense fluorescence corresponding to the supramolecular probes primarily in the proximal tubule, suggesting that the supramolecular probes undergo renal clearance in vivo largely through renal secretion pathway. This unique clearance property not only extends its retention time within the body, facilitating enhanced accumulation at lesion sites and improved imaging efficacy compared to probes cleared rapidly through MPS or glomerular filtration, but also ensures minimal toxicity, in contrast to probes undergoing hepatic clearance, which often induce adverse toxic effects (*40*, *41*).

Real-time imaging of AKI

AKI, marked by a rapid decline in renal function, is a substantial global health concern that affects over 13 million people and resulting in 1.7 million deaths annually worldwide, with clinical drugs accounting for over 20% of these cases (*42*, *43*). A timely and accurate diagnosis in the early stages of AKI is crucial for initiating protective interventions and predicting potential drug toxicity (*44*). Although serum creatinine (CREA) and blood urea nitrogen (BUN) serve as standard biomarkers to assess kidney dysfunction, their applicability for early diagnosis is limited, and they can be influenced by various diseases (*45*). The renal tubules, notable for their high metabolic rates, are highly susceptible to drug-induced damage during secretion and reabsorption processes (*46*). This damage often originates from drug accumulation on the tubular surface or within cells. For instance, cisplatin, a prevalent anticancer drug, tends to accumulate in the kidneys (*47*), subsequently causing irreversible acute necrosis of the proximal tubular epithelial cells during AKI (*48*). In this context, our supramolecular probes distinguish themselves with a unique clearance mechanism through tubular secretion and reabsorption, offering a notable advantage over the recently reported optical probes (*49*, *50*), which depend on the glomerular filtration mechanism for the diagnosis of AKI (*51*–*53*). We anticipate that our supramolecular probes will be retained in the kidneys when faced with compromised renal function caused by AKI, leading to enhanced kidney fluorescence and reduced bladder fluorescence, thereby facilitating a more accurate and earlier diagnosis.

To explore this diagnostic potential, we conducted real-time fluorescence imaging on cisplatin-treated mice. After administering various doses of cisplatin intraperitoneally, Cy7.5AA@CDP was injected intravenously 24 hours after cisplatin treatment (fig. S45). Notably, after a 20 mg/kg dose of cisplatin, there was a marked increase in kidney signals, accompanied by a substantial reduction in bladder signals, indicative of nephrotoxicity. We then proceeded with whole-body longitudinal living fluorescence imaging to monitor the

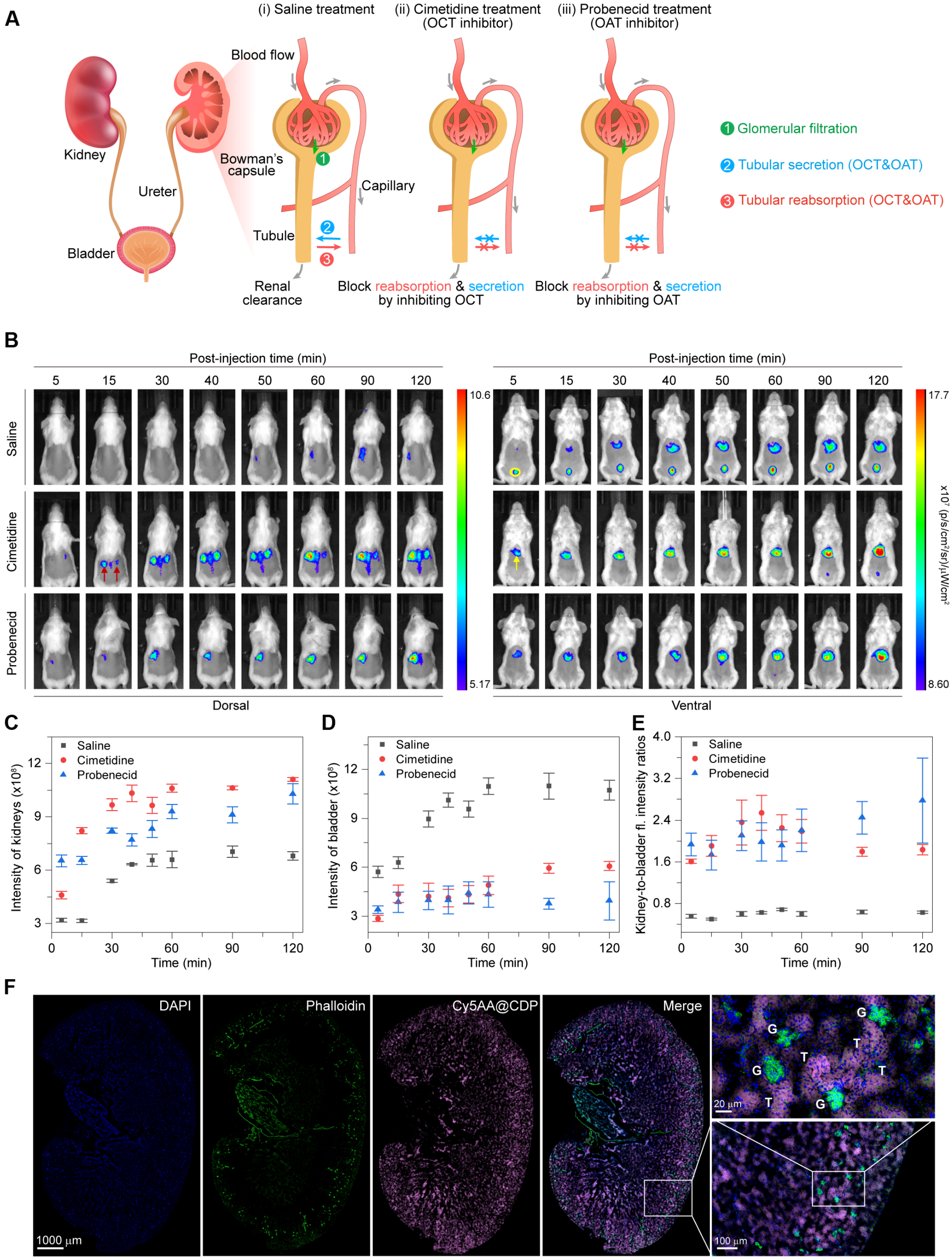

Fig. 4. Renal clearance mechanism of the supramolecular probes. (**A**) Schematic illustration of the renal clearance mechanisms, including glomerular filtration, tubular secretion, and tubular reabsorption. (**B**) Representative fluorescence images of live mice at diverse intervals after intraperitoneal administration of either saline, organic cation transporter (OCT) cimetidine (150 mg/kg), or organic anion transporter (OAT) probenecid (150 mg/kg), followed by an intravenous injection of Cy7.5AA@CDP (100 μM, 100 μl). Red and yellow arrows highlight the locations of the kidneys and liver, dorsally and ventrally, respectively, while the yellow circle indicates the bladder on the ventral side. (**C** and **D**) Time-dependent fluorescence intensities measured from the kidneys (C) and bladder (D) after administering Cy7.5AA@CDP to mice that were pretreated with either saline, cimetidine, or probenecid. (**E**) Time-dependent ratios of kidney-to-bladder fluorescence intensity post-injection of Cy7.5AA@CDP in mice pretreated with saline, cimetidine, or probenecid. (**F**) Fluorescence microscopy images of entire kidney sections from mice post-intravenous injection of Cy5AA@CDP. Fluorescent Alexa Fluor 568 phalloidin was used to highlight the abundant filamentous actin present in both glomeruli and the brush border. DAPI, 4′,6-diamidino-2-phenylindole; G, glomerulus; T, tubules. Intense fluorescence attributed to Cy5AA@CDP was primarily detected in the proximal tubule. This observation suggests that the supramolecular probe primarily follows a renal secretion mechanism for in vivo renal clearance. The data are the means ± SD. $n = 3$ independent mice.

progression of this induced damage (Fig. 5, A and B). Within the saline-treated control group, after Cy7.5AA@CDP administration, a progressively intensifying fluorescent signal became evident in the bladder (Fig. 5C). This signal revealed a gradual rise and plateaued over 2 hours, consistent with the fluorescence pattern in the kidneys. In contrast, 6 hours after cisplatin treatment, the introduction of Cy7.5AA@CDP resulted in a continuous enhancement in kidney signals, manifesting a fluorescence intensity surpassing that of the control group (Fig. 5D). Meanwhile, minimal signal enhancement is observed in the bladder. This trend persisted in mice treated with cisplatin for 12 and 24 hours, highlighting the prolonged retention of Cy7.5AA@CDP in the kidneys and diminished renal clearance after cisplatin exposure. Evaluating the kidney-to-bladder fluorescence intensity ratios after cisplatin exposure at 6-, 12-, and 24-hour intervals yielded ratios of 2.75 ± 0.29, 3.97 ± 0.17, and 5.51 ± 0.67, respectively, after 10 min Cy7.5AA@CDP administration (Fig. 5E). These values are markedly increased relative to the control group's ratio of 0.73 ± 0.07. This observation aligns with the reduced renal clearance of Cy7.5AA@CDP in cisplatin-treated mice for all examined doses and durations (fig. S46). Furthermore, histological examinations revealed discernible damage (fig. S47), including hyaline alterations and tubular edema, 48 hours after cisplatin treatment, suggesting that prolonged exposure to cisplatin led to its accumulation within renal tubules, inducing damage and consequently impeding the clearance of Cy7.5AA@CDP.

The translational potential of Cy7.5AA@CDP was further supported by online urinalysis, which entails the direct measurement of the fluorescent signal from the excreted Cy7.5AA@CDP in the urine of cisplatin-treated mice (Fig. 5F). A noticeable signal reduction compared to the control mice manifested 6 hours after cisplatin treatment. This decline in fluorescence continued, with reductions of 2.2-fold and 4.8-fold observed at 12 and 24 hours after cisplatin administration, respectively (Fig. 5G). This observation reflected findings from real-time fluorescence imaging, collectively indicating a decelerated clearance rate of Cy7.5AA@CDP after cisplatin-induced kidney damage. In comparison, traditional metrics CREA and BUN required 72 hours to provide a notable signal (Fig. 5, H and I). Thus, optical imaging and online urinalysis with Cy7.5AA@CDP offered a substantially earlier detection of cisplatin-induced AKI, advancing ahead of conventional methods by ~66 hours and exceeding other optical probes reported in prior studies.

Tumor accumulation and image-guided surgical resection

Currently, malignant tumors are among the leading health threats worldwide (*54*). Although the FDA-approved optical probe ICG has been extensively used in cardiovascular studies, liver function investigations, and intraoperative imaging, it still has limitations such as a short blood circulation half-life, rapid clearance, and poor stability, necessitating multiple injections to achieve an ideal imaging SBR (*55–57*). In contrast, our supramolecular probes, endowed with enhanced optical properties, extended blood circulation half-life, nanoscale dimensions favorable for potential enhanced permeability and retention (EPR) effects, and the capability of renal clearance, exhibit substantial promise for the accurate and convenient imaging of tumors.

NIR-II imaging offers enhanced spatial resolution and imaging depth due to reduced scattering compared to the NIR-I window (*58*, *59*). ICG captures only about 5% of the total emission in the NIR-II window (*60*), yet it remains effective for NIR-II imaging in applications such as lymphatic and vascular imaging (*61*, *62*). Given the structural similarities between our guest fluorophores and ICG, we explore the potential of the supramolecular probe in NIR-II imaging. In the penetration depth assay, Cy7.5AA@CDP displayed remarkable imaging depth and brightness in aqueous solutions, delineated the capillary's sharp contours with an SBR reaching 62, whereas ICG only achieved an SBR of 8 (fig. S48). Moreover, 1 hour after intravenous injection, NIR-II fluorescence imaging of the kidney with Cy7.5AA@CDP revealed a substantially improved in vivo SBR of 8.8 (fig. S49). Furthermore, we re-examined the in vivo transport profiles and clearance mechanism of Cy7.5AA@CDP, as well as its potential for AKI diagnosis, using NIR-II imaging. The results obtained are consistent with the imaging studies previously conducted in the NIR-I window, validating the reliability and robustness of our findings (figs. S50 to S52). These findings suggest that Cy7.5AA@CDP exhibits superior NIR-II optical attributes, positioning it as a promising candidate for effective NIR-II imaging in vivo.

Motivated by the prolonged circulation time and precise nanoscale dimensions, we investigated the in vivo tumor accumulation properties of the supramolecular probe. Upon intravenous injection of Cy7.5AA@CDP into mice with subcutaneous CT-26 cancer xenografts, we proceeded with NIR-II fluorescence imaging (Fig. 6A). A steady increase in fluorescence within the tumor, with a clear NIR-II signal noticeable at 1 hour, peaks at 24 hours. The imaging SBR for Cy7.5AA@CDP remained consistent at ~6.4 from 1 to 24 hours after injection, a value that was 4.6 times greater than the control group treated with ICG (averaging 1.4) (Fig. 6B). Moreover, experiments using a lower supramolecular probe dose still yielded imaging efficacy comparable to that of higher doses (fig. S53). By 48 hours, the tumor sites remained intensely fluorescent, in stark contrast to the kidney and liver, where signals had considerably receded. These findings suggest the effective passive accumulation of our supramolecular probe within tumor sites, likely attributed to the combined advantages of prolonged circulation time and the EPR effect (*63*), which together facilitate optimal imaging outcomes without the need for high injection dosages or frequent administration.

Image-guided surgery offers a powerful approach for precise tumor removal (*64–66*). Given the superior accumulation of Cy7.5AA@CDP in tumor sites compared to ICG, it emerges as a promising agent for fluorescence-guided surgical resection. To assess this potential, surgical resection was conducted on mice bearing a subcutaneous tumor 48 hours following the intravenous injection of the supramolecular probe (Fig. 6C). Under the guidance of the NIR-II fluorescence, the tumor boundaries were clearly delineated, leading to an efficient and accurate tumorectomy (Fig. 6D). The encapsulation of tumor remained intact with no evident distant metastasis (Fig. 6E). Further fluorescence imaging studies on tumor sections revealed that the fluorescence signals from Cy7.5AA@CDP were confined to the tumor tissues and were not present in adjacent normal tissues, suggesting the precise applicability of Cy7.5AA@CDP in surgical navigation (fig. S54). Post-surgery, the mice displayed robust health, no signs of tumor recurrence, and prolonged postoperative survival (figs. S55 and S56). These findings highlight the proficiency of NIR-II fluorescence imaging in ensuring comprehensive tumor removal while minimizing collateral damage to nearby healthy tissues.

We proceeded to evaluate orthotopic breast cancer models to assess the potential of supramolecular probes in primary tumor detection and deep surgical navigation. We established an orthotopic cancer model by injecting luciferase-expressing 4T1 cells directly into the fourth pair of mammary fat pads in mice (*67*) and administered

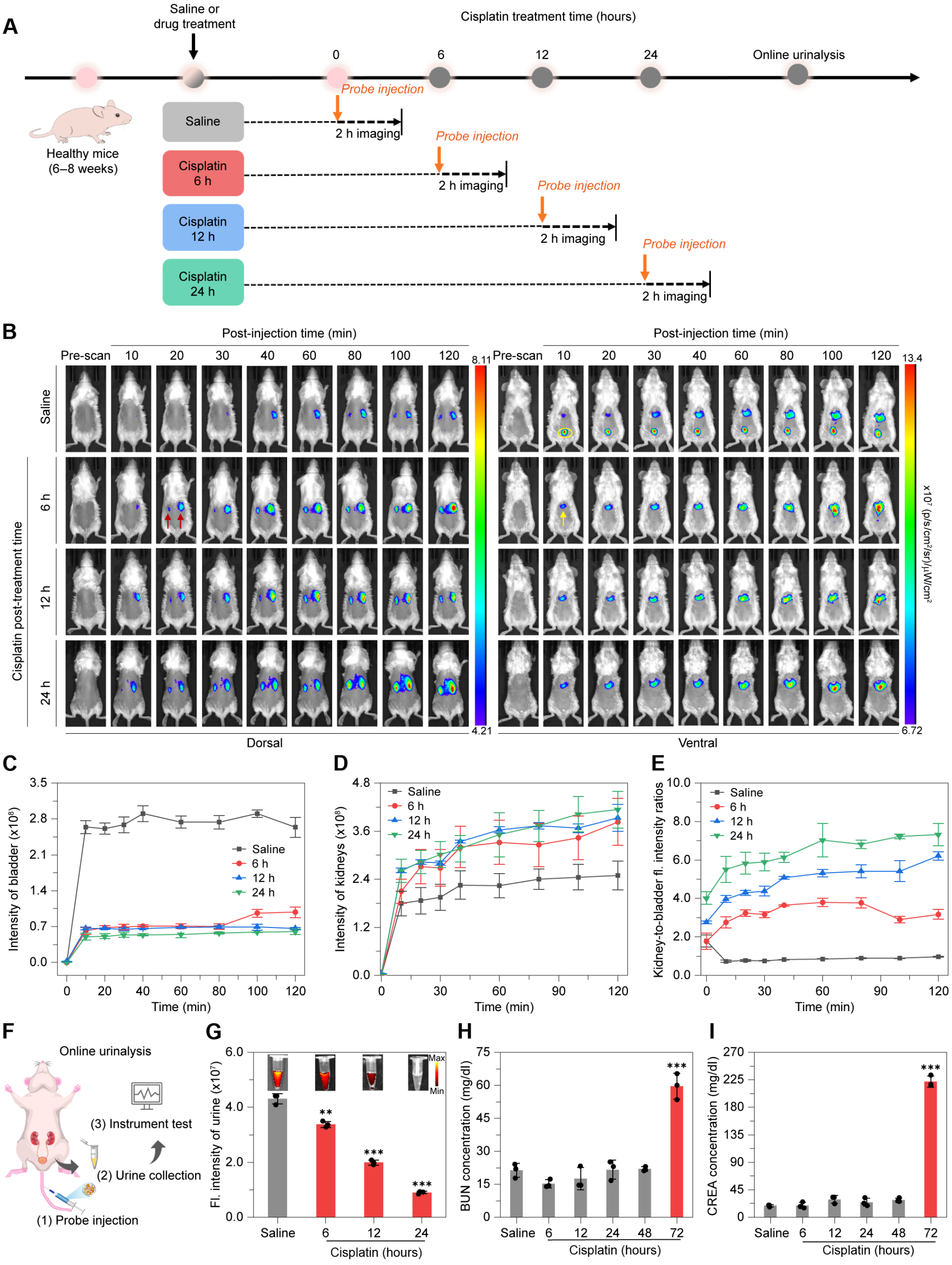

Fig. 5. Real-time in vivo fluorescence imaging of cisplatin-induced AKI using the supramolecular probes. (**A**) Schematic illustration of the development of cisplatin induced AKI mouse model and subsequent fluorescence imaging at various post-treatment intervals. (**B**) Representative in vivo fluorescence images of mice post-injection with either saline or cisplatin, followed by an intravenous Cy7.5AA@CDP injection (100 μM, 75 μl). Red and yellow arrows highlight the kidneys and liver, on the dorsal and ventral sides, respectively. The yellow circle denotes the bladder on the ventral side. (**C** and **D**) Time-resolved fluorescence intensities of the bladder (C) and kidneys (D) following Cy7.5AA@CDP injection in mice administered with saline or cisplatin. (**E**) Time-dependent kidney-to-bladder fluorescence intensity ratios in mice post-intraperitoneal administration of saline or cisplatin, followed by intravenous Cy7.5AA@CDP injection. (**F**) Schematic illustration of the online urinalysis procedure. (**G**) Fluorescence images and their corresponding intensities from urine samples of mice treated with cisplatin or saline, both followed by an intravenous injection of Cy7.5AA@CDP. Statistical significance was calculated by one-way analysis of variance (ANOVA) with a Tukey post hoc test; saline versus cisplatin-treated groups, $^{**}P < 0.01$; $^{***}P < 0.001$. (**H** and **I**) Alterations in BUN (H) and CREA (I) concentrations in mice post treatment with saline or cisplatin, captured at $t = 6$-, 12-, 24-, 48-, and 72-hour intervals. Statistical significance was calculated by one-way ANOVA with a Tukey post hoc test; saline versus cisplatin-treated groups, $^{***}P < 0.001$. The data are the means ± SD. $n = 3$ independent mice.

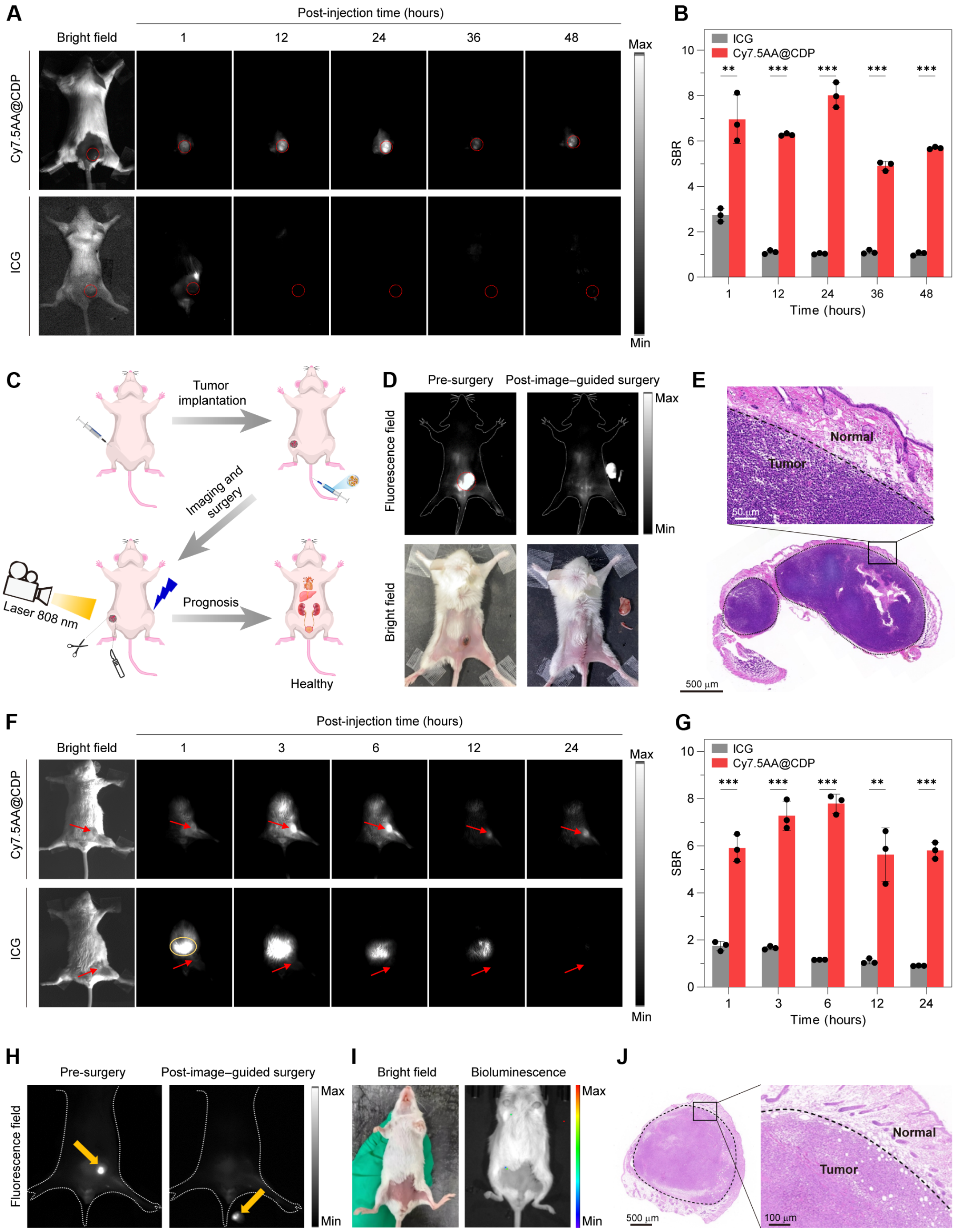

Fig. 6. NIR-II fluorescence-guided in vivo tumor imaging and resection using Cy7.5AA@CDP. (**A**) Time-dependent NIR-II fluorescence imaging of mice bearing subcutaneous CT-26 tumor xenografts post-intravenous administration of either Cy7.5AA@CDP or ICG. Red circles indicate subcutaneous CT-26 tumor. (**B**) Analysis of SBRs for Cy7.5AA@CDP and ICG during NIR-II imaging in mice with subcutaneous CT-26 tumors. (**C**) Illustrated workflow of tumor implantation followed by NIR-II fluorescence-assisted surgical navigation for tumor removal in mice. (**D**) Identification and subsequent resection of subcutaneous CT-26 tumor guided by the NIR-II fluorescence of Cy7.5AA@CDP. (**E**) Histological assessment (H&E staining) of excised subcutaneous CT-26 tumor sections confirming intact encapsulation and absence of remote metastasis. (**F**) Time-resolved NIR-II fluorescence images of mice with orthotopic breast cancer following intravenous injection of Cy7.5AA@CDP or ICG. Red arrows indicate the tumor site. (**G**) Analysis of SBR for Cy7.5AA@CDP and ICG during NIR-II imaging in mice bearing orthotopic breast tumors. (**H**) Identification and subsequent resection of orthotopic breast cancer tumor guided by the NIR-II fluorescence of Cy7.5AA@CDP. The post-injection time is 24 hours to ensure high imaging SBR by the supramolecular probe while reducing the fluorescence signals resulting from nonspecific accumulation of the probe. Yellow arrows highlight the tumor. (**I**) Post-surgical bioluminescence and bright-field images of treated mice. The absence of a bioluminescence signal suggests successful removal of the orthotopic breast tumor. (**J**) Histological assessment (H&E staining) of dissected orthotopic breast tumor samples, confirming comprehensive removal and the absence of residual or metastatic cancerous sites. The data are the means ± SD. $n = 3$ independent mice; two-tailed Student's t test; ICG-treated versus Cy7.5AA@CDP-treated groups, $^{**}P < 0.01$; $^{***}P < 0.001$.

Cy7.5AA@CDP or ICG intravenously. NIR-II fluorescence images at various time points were then captured (Fig. 6F). Predictably, mice treated with Cy7.5AA@CDP exhibited a steady enhancement in fluorescence signals at the tumor site. A notable imaging SBR above 7.0 between 3 and 6 hours after injection highlighted the efficient passive accumulation of Cy7.5AA@CDP within the orthotopic breast cancer (Fig. 6G). Conversely, ICG-injected mice showed barely any fluorescence changes at the tumor site, but the liver displayed pronounced and increasing signals. The correlation between imaging duration and SBR in mice was then examined, which pinpointed the optimal surgical navigation window for orthotopic breast cancer to 24 hours after injection. Leveraging the precision of the NIR-II imaging system, we accomplished complete resection of in situ mammary carcinoma in mice (Fig. 6H). Under fluorescence guidance, no minor or metastatic lesions were detected. After the surgical procedure, the mice's wounds were sutured, followed by an intraperitoneal injection of luciferase substrate. The absence of a bioluminescence signal, which arises only from the reaction between the luciferase substrate and luciferase expressed by tumor cells (*67*), confirmed the complete resection of the tumor (Fig. 6I). Histological evaluations further substantiated that the tumor's encapsulation was intact (Fig. 6J). Post-surgical observations indicated that the mice were in good health (fig. S57).

DISCUSSION

In summary, by using robust multivalent host-guest interactions between cyanine dyes and CDP, we present a straightforward yet potent approach to enhance the stability, optical properties, and transport efficiency of molecular optical probes in living systems. With association constants reaching 10^7 M^{-1}, these interactions ensure the structural robustness of the probes, overcoming a notable limitation often faced by supramolecular systems in biological environments. Moreover, CDP encapsulation prevents dye aggregation and protects it from the external environment, leading to a 7.8-fold increase in absolute fluorescence quantum yield compared to the clinically used dye ICG, addressing a key challenge in the application of optical imaging probes for clinical diagnostics.

CDP encapsulation introduces hydroxyl groups to the surface of the supramolecular probe, resulting in a near-neutral surface charge and increased polarity. These attributes substantially reduce the potential for MPS sequestration and minimize interactions with serum proteins. Further investigations reveal that the supramolecular probes undergo renal clearance predominantly through tubular secretion, attributed to their average hydrodynamic diameter of ~400 nm (*37*, *38*). This unique clearance property not only extends their retention time within the body compared to probes cleared rapidly through MPS or glomerular filtration but also substantially prolongs their circulation in the bloodstream, with an elimination half-life exceeding 210 min. These properties ensure that a higher proportion of the administered dose remains available for interaction with target lesions, resulting in a substantially higher SBR, which is a critical factor in achieving accurate imaging outcomes.

Consequently, the imaging and diagnostic capabilities of the probes are substantially enhanced, as demonstrated in the early detection of drug-induced AKI, where abnormalities were detectable as early as 6 hours, marking a notable 66-hour improvement over current clinical strategies. This improvement offers a reliable method for the mechanistic study of nephrotoxicity and enables timely renoprotective interventions in patients with early-stage AKI. In addition, the supramolecular probe achieves higher imaging SBRs across a spectrum of tumor models, facilitating precise excision in both subcutaneous and orthotopic tumor models under the guidance of NIR-II fluorescence.

Thus, our design introduces a generic strategy in the development of optical materials for accurate clinical disease imaging and holds promise for elucidating physiological and pathological mechanisms inherent in tumor progression, such as growth, invasion, and metastasis. Furthermore, the robust and versatile design principles underscored by our research encourage exploration into other molecular imaging modalities where evasion of MPS capture can be similarly beneficial. These include MRI, x-ray CT, and PET, ensuring safe and accurate lesion imaging crucial for clinical diagnostics, or the development of precise drug delivery systems that optimize the delivery of anticancer or immunotherapeutic agents to tumor lesions for enhanced treatment efficacy and safety.

In conclusion, our study not only demonstrates the potential of multivalent supramolecular probes to enhance optical imaging but also sets a foundation for future developments in clinical imaging and diagnostics. By addressing critical limitations related to MPS sequestration and providing a scalable platform for integrating various imaging modalities, this research promises substantial advancements in clinical diagnostics and therapeutic strategies, ultimately enhancing our ability to diagnose, monitor, and treat diseases with unprecedented precision.

MATERIALS AND METHODS

Materials and equipment

Unless otherwise stated, all chemical reagents were obtained from commercial suppliers and were used without further purification. The β-cyclodextrin polymer (CDP) was purchased from Sigma-Aldrich (United States). Solvents were purified using standard methods before use. All experiments used twice-distilled water. Matrix-assisted laser desorption/ionization time-of-flight (MALDI-TOF) analyses were conducted using a Bruker Ultraflex TOF/TOF instrument. High-resolution electrospray ionization (ESI-HRMS) mass spectra were acquired with an Orbitrap Exploris 120 (Thermo Fisher Scientific). Nuclear magnetic resonance (NMR) spectra were recorded using a Bruker-400 spectrometer with tetramethylsilane as an internal standard. Absorption spectra were obtained using a UV-1800 spectrophotometer (Shimadzu Corporation, Japan). Fluorescence spectra were measured at room temperature using a HITACHI F7000 fluorescence spectrophotometer and an Edinburgh FLS1000 machine. pH measurements were conducted using a PHS-3C pH meter (INESA Instrument). In vivo imaging of living mice was conducted using an IVIS Lumina XR (IS124N6071) imaging system. The NIR-II images were captured using a custom small animal imaging system equipped with a 640- × 512-pixel two-dimensional (2D) InGaAs NIRvana charge-coupled device (CCD) camera. Thin-layer chromatography analysis was carried out on silica gel plates, and column chromatography was conducted using silica gel (mesh 200-300), both sourced from Yantai Jiangyou Silica Gel Development Company Limited. SEM images were captured using a MIRA3 LMH (TESCAN), and TEM images were obtained with a JEM-F200 (JEOL). DLS and zeta potential measurements were conducted using a Malvern Zetasizer Nano ZS90 (Malvern). The absolute fluorescence quantum yields of the compounds in solutions were measured using an integrating sphere by an Edinburgh FLS1000 machine.

General procedure of the synthesis of guest fluorophores

The cyanine dyes (fig. S58) were synthesized based on previously described methods (fig. S59) (*68*).

General procedure for the synthesis of compound Cy3AA and Cy3.5AA

(B)1-COOH (2.0 mmol), *N*,*N*′-diphenylformamidine (1.0 mmol), and AcONa (2.0 mmol) were dissolved in Ac_2O (20.0 ml). Next, the mixture was stirred at 60°C for 4 to 6 hours. After cooling, Cy3/3.5COOH precipitated as a red powder and was collected by filtration (40 to 45%). Then, compound Cy3/3.5COOH (0.5 mmol), *O*-(7-azabenzotriazol-1-yl)-*N*,*N*,*N*′,*N*′-tetramethyluronium hexafluorophosphate (HATU; 0.75 mmol), and triethylamine (Et_3N; 200 μl) were dissolved in anhydrous CH_2Cl_2, and the mixture was stirred at room temperature for 0.5 hours. Subsequently, a CH_2Cl_2 solution of amantadine (1.0 mmol) was added dropwise and the reaction mixture was stirred for another 3 hours at room temperature. The solvent was removed under vacuum, and the mixture was purified by column chromatography on silica gel with CH_2Cl_2 and methanol (100/1, v/v) to offer compounds Cy3AA (88% yield) and Cy3.5AA (85% yield). Cy3AA: ^{1}H NMR (400 MHz, methanol-d_4) δ 8.54 (t, *J* = 13.4 Hz, 1H), 7.54 (d, *J* = 7.4 Hz, 2H), 7.47–7.42 (m, 2H), 7.37 (d, *J* = 7.8 Hz, 2H), 7.31 (t, *J* = 7.4 Hz, 2H), 6.50 (d, *J* = 13.5 Hz, 2H), 4.17 (t, *J* = 7.2 Hz, 4H), 3.21 (q, *J* = 7.3 Hz, 2H), 2.20 (t, *J* = 6.9 Hz, 4H), 1.98 (d, *J* = 8.3 Hz, 16H), 1.87–1.82 (m, 3H), 1.77 (s, 12H), 1.66 (s, 9H), and 1.29 (d, *J* = 2.5 Hz, 8H). ^{13}C NMR (101 MHz, methanol-d_4) δ 175.8, 152.0, 143.2, 142.0, 129.8, 126.6, 123.3, 112.3, 103.7, 52.6, 50.5, 44.7, 42.1, 37.2, 37.0, 32.6, 30.7, 28.2, 27.6, 24.1, 23.5, and 14.2. HRMS (ESI): mass/charge ratio (*m*/*z*) calcd for $C_{53}H_{71}N_4O_2^+$: 795.5572, found: 795.5551. Cy3.5AA: ^{1}H NMR (400 MHz, methanol-d_4) δ 8.80 (t, *J* = 13.5 Hz, 1H), 8.32 (d, *J* = 8.5 Hz, 2H), 8.05 (dd, *J* = 12.1, 8.4 Hz, 3H), 7.75–7.71 (m, 2H), 7.71–7.63 (m, 4H), 7.54 (t, *J* = 7.5 Hz, 2H), 6.57 (d, *J* = 13.5 Hz, 2H), 4.32 (q, *J* = 6.4 Hz, 4H), 4.24 (dd, *J* = 5.7, 2.1 Hz, 1H), 2.24 (t, *J* = 6.9 Hz, 3H), 2.12 (d, *J* = 3.7 Hz, 10H), 1.96 (s, 13H), 1.84–1.78 (m, 3H), 1.64 (s, 8H), 1.49–1.45 (m, 3H), 1.38–1.36 (m, 4H), and 1.33–1.30 (m, 10H). ^{13}C NMR (101 MHz, methanol-d_4) δ 177.2, 174.5, 140.6, 134.8, 133.4, 132.2, 132.1, 131.7, 131.0, 129.7, 129.1, 128.7, 126.2, 123.2, 112.1, 103.1, 66.4, 52.6, 52.2, 44.9, 42.1, 40.0, 37.2, 37.1, 31.4, 30.6, 30.5, 29.9, 27.9, 24.8, 24.1, 23.8, 23.5, 20.1, 14.2, 13.8, and 11.2. HRMS (ESI): *m*/*z* calcd for $C_{61}H_{75}N_4O_2^+$: 895.5855, found: 895.5862.

General procedure for synthesis of compound Cy5AA and Cy5.5AA

(B)1-COOH (2.0 mmol), *N*-(3-(phenylamino)allylidene)aniline hydrochloride (1.0 mmol), and AcONa (2.0 mmol) were dissolved in CH_3CN (20.0 ml). Next, the mixture was stirred at 75°C for 5 hours. After cooling, Cy5/5.5COOH precipitated as a blue powder and was collected by filtration (35 to 40% yield). Then, compound Cy5/5.5COOH (0.5 mmol), HATU (0.75 mmol), and Et_3N (200 μl) were dissolved in anhydrous CH_2Cl_2, and the mixture was stirred at room temperature for 0.5 hours. Subsequently, a CH_2Cl_2 solution of amantadine (1.0 mmol) was added dropwise, and the reaction mixture was stirred for another 3 hours at room temperature. The solvent was removed under vacuum, and the mixture was purified by column chromatography on silica gel with CH_2Cl_2 and methanol (100/2, v/v) to offer compounds Cy5AA (83% yield) and Cy5.5AA (80% yield). Cy5AA: ^{1}H NMR (400 MHz, methanol-d_4) δ 8.23 (t, *J* = 13.1 Hz, 2H), 7.49 (d, *J* = 7.4 Hz, 2H), 7.39 (d, *J* = 8.4 Hz, 2H), 7.31 (d, *J* = 7.9 Hz, 2H), 7.26 (t, *J* = 7.4 Hz, 2H), 6.66 (t, *J* = 12.4 Hz, 1H), 6.27 (d, *J* = 13.7 Hz, 2H), 4.11 (d, *J* = 4.4 Hz, 3H), 3.11 (s, 3H), 2.81 (s, 2H), 2.18 (t, *J* = 6.8 Hz, 4H), 2.01 (s, 6H), 1.98 (s, 10H), 1.84–1.79 (m, 3H), 1.73 (s, 13H), and 1.67 (s, 11H). ^{13}C NMR (101 MHz, methanol-d_4) δ 155.2, 143.4, 129.5, 126.7, 126.0, 123.2, 111.9, 104.4, 52.6, 50.4, 44.4, 42.1, 40.1, 37.3, 37.0, 30.7, 27.7, 27.4, and 24.1. HRMS (ESI): *m*/*z* calcd for $C_{55}H_{73}N_4O_2^+$: 821.5729, found: 821.5707. Cy5.5AA: ^{1}H NMR (400 MHz, methanol-d_4) δ 8.34 (t, *J* = 13.1 Hz, 2H), 8.25 (d, *J* = 8.5 Hz, 2H), 8.03–7.97 (m, 4H), 7.69–7.58 (m, 5H), 7.50 (d, *J* = 7.5 Hz, 2H), 7.29 (s, 1H), 6.71 (t, *J* = 12.1 Hz, 1H), 6.34 (d, *J* = 13.8 Hz, 2H), 4.31 (d, *J* = 6.6 Hz, 1H), 4.25 (t, *J* = 7.0 Hz, 4H), 2.21 (t, *J* = 6.8 Hz, 4H), 2.03 (s, 12H), 1.97 (s, 18H), 1.92–1.87 (m, 4H), 1.80–1.73 (m, 5H), and 1.65 (s, 10H). ^{13}C NMR (101 MHz, methanol-d_4) δ 175.7, 174.5, 174.4, 154.0, 140.8, 134.9, 133.2, 132.1, 131.5, 130.9, 129.7, 129.3, 128.5, 126.6, 125.9, 123.1, 111.9, 66.4, 52.7, 52.6, 52.2, 44.5, 42.1, 42.1, 37.3, 37.1, 37.0, 32.9, 30.6, 30.5, 30.3, 27.8, 27.7, 27.5, 24.1, 23.5, 20.0, 14.2, and 13.9. HRMS (ESI): *m*/*z* calcd for $C_{63}H_{77}N_4O_2^+$: 921.6042, found: 921.6017.

General procedure for synthesis of compound Cy7AA and Cy7.5AA

(B)1-COOH (2.0 mmol), *N*-((2*E*,4*E*)-5-(phenylamino)penta-2,4-dien-1-ylidene)aniline hydrochloride (1.0 mmol), and AcONa (2.0 mmol) were dissolved in EtOH (20.0 ml). Next, the mixture was stirred at 78°C for 6 hours. After cooling, Cy7/7.5COOH precipitated as a green powder and was collected by filtration (40 to 45% yield). Then, compound Cy7/7.5COOH (0.5 mmol), HATU (0.75 mmol), and Et_3N (200 μl) were dissolved in anhydrous CH_2Cl_2, and the mixture was stirred at room temperature for 0.5 hours. Subsequently, a CH_2Cl_2 solution of amantadine (1.0 mmol) was added dropwise and the reaction mixture was stirred for another 3 hours at room temperature. The solvent was removed under vacuum, and the mixture was purified by column chromatography on silica gel with CH_2Cl_2 and methanol (100/1, v/v) to offer compounds Cy7AA (80% yield) and Cy7.5AA (75% yield). Cy7AA: ^{1}H NMR (400 MHz, chloroform-*d*) δ 7.72 (t, *J* = 12.2 Hz, 2H), 7.42 (d, *J* = 10.9 Hz, 1H), 7.38–7.28 (m, 4H), 7.18 (t, *J* = 7.4 Hz, 2H), 7.08 (d, *J* = 7.9 Hz, 2H), 6.61 (t, *J* = 11.8 Hz, 2H), 6.12 (d, *J* = 13.4 Hz, 2H), 5.58 (s, 2H), 3.94 (d, *J* = 6.8 Hz, 4H), 3.48 (s, 4H), 2.80 (s, 1H), 2.23 (t, *J* = 6.5 Hz, 4H), 2.01 (d, *J* = 15.6 Hz, 17H), 1.78 (s, 8H), and 1.64 (s, 20H). ^{13}C NMR (101 MHz, chloroform-*d*) δ 171.8, 171.3, 142.3, 140.9, 128.8, 126.8, 124.9, 122.2, 110.5, 51.9, 50.9, 49.1, 44.1, 41.5, 38.7, 36.7, 36.4, 34.7, 29.8, 29.5, 27.9, 27.0, 26.8, 25.3, 23.0, 22.8, 22.7, 22.7, 14.2, and 11.5. HRMS (ESI): *m*/*z* calcd for $C_{57}H_{75}N_4O_2^+$: 847.5885, found: 847.5862. Cy7.5AA: ^{1}H NMR (400 MHz, chloroform-*d*) δ 8.10 (d, *J* = 8.5 Hz, 2H), 7.92 (d, *J* = 8.6 Hz, 5H), 7.85 (d, *J* = 12.6 Hz, 2H), 7.59 (t, *J* = 7.6 Hz, 2H), 7.45 (t, *J* = 7.5 Hz, 2H), 7.38 (d, *J* = 8.8 Hz, 2H), 6.71–6.60 (m, 2H), 6.17 (d, *J* = 13.3 Hz, 2H), 5.58 (s, 2H), 4.08 (d, *J* = 7.0 Hz, 4H), 2.27 (t, *J* = 6.6 Hz, 4H), 2.03 (d, *J* = 12.7 Hz, 17H), 1.96 (s, 11H), 1.86 (dd, *J* = 16.8, 10.9 Hz, 8H), and 1.66 (s, 14H). ^{13}C NMR (101 MHz, chloroform-*d*) δ 171.7, 131.7, 130.7, 130.0, 128.9, 127.7, 124.9, 122.1, 110.5, 51.9, 50.9, 41.4, 36.7, 36.4, 29.4, 27.5, 27.0, and 23.0. HRMS (ESI): *m*/*z* calcd for $C_{65}H_{79}N_4O_2^+$: 947.6198, found: 947.6182.

Determination of apparent association constants

For the determination of the apparent association constants (K_a), the fluorescence spectra of the supramolecular probes were assessed at various guest fluorophores concentrations. The relationship between the concentration and fluorescence intensity was then illustrated as a scatter plot. Nonlinear curve fitting, using the Hill 1 function in Origin2018, was used for data analysis (*27*). According to the Hill 1 function equation, the dissociation constant (K_d) corresponds to the

concentration at half of the maximum fluorescence intensity, and the complexation constant (K_a) is determined as $K_a = 1/K_d$. The calculation equation was estimated as

$$y = \frac{A + R \times [c]}{K_d + [c]}$$

where K_d is the apparent dissociation constant, A is a constant associated with multivalent supramolecular assembly systems, R is defined as the difference in fluorescence intensity before and after the reaction of supramolecular probes, and $[c]$ is the concentration of supramolecular probes.

ITC analysis on the supramolecular interactions

ITC experiments were performed using a NANO ITC instrument (TA Instruments, United States). Before each experiment, the sample cell and syringe were thoroughly cleaned with PBS containing 5% dimethyl sulfoxide (DMSO) (blank solvent). For the control group, Cy7.5AA was titrated with PBS containing 5% DMSO, with Cy7.5AA in the syringe and the blank solvent in the sample cell. During the experiment, the syringe contained Cy7.5AA (50 μl, 70 μM), which was titrated 25 times into the sample cell containing host molecules (10 μM), with each addition being 2 μl. The temperature was maintained at 25°C, with a stirring speed of 350 rpm, and injections were made every 2 min. The data obtained were analyzed using the independent model to determine parameters such as the dissociation constant ($1/K_a$) and stoichiometry ratio (n).

Preparation of the supramolecular probe

The multivalent supramolecular probe Cy7.5AA@CDP was prepared by combining Cy7.5AA with CDP (final CD concentration of 10 mM) in a 1-ml aqueous solution. This mixture was agitated at room temperature for 3 hours, followed by ultrafiltration using a filter with a molecular weight cutoff of 100 kDa. Subsequently, Cy7.5AA@CDP was isolated as a solid via freeze-drying. Throughout this manuscript, the expression of Cy7.5AA@CDP concentration is based on the amount of the encapsulated Cy7.5AA dye.

Determination of loading efficiency

To ascertain the loading efficiency of Cy7.5AA@CDP for in vivo applications, we dissolved 1.0 mg of the solid Cy7.5AA@CDP in 1.0 ml of PBS. The absorption spectrum of this resultant solution exhibited well-defined bands, with a prominent peak at 790 nm, characteristic of Cy7.5AA, and an absorbance of 1.0363 (fig. S7A).

To quantify the concentration of Cy7.5AA in the Cy7.5AA@CDP complex, we established a standard curve based on the absorbance measurements of varying concentrations of Cy7.5AA@CDP at 790 nm (fig. S7B). The relationship between the absorbance (A_{790}) and concentration (c) is described by the equation

$$A_{790} = 0.1118c - 0.019$$

In this equation, c represents the concentration of Cy7.5AA in the Cy7.5AA@CDP complex, while A_{790} denotes the absorbance of this complex in PBS. Applying this standard curve, the concentration of Cy7.5AA in our test solution was determined to be 9.4 μM.

Consequently, the mass of Cy7.5AA in the solution was calculated using the formula

$$m_{\mathrm{Cy7.5AA}} = c_{\mathrm{Cy7.5AA}} \times v \times M_{\mathrm{Cy7.5AA}}$$

Here, $m_{\mathrm{Cy7.5AA}}$ is the mass of Cy7.5AA, $c_{\mathrm{Cy7.5AA}}$ is its concentration of Cy7.5AA (9.4 μM) in the solution, v is the volume of the solution (1.0 ml), and $M_{\mathrm{Cy7.5AA}}$ is the molar mass of Cy7.5AA (947 g mol^{-1}). Therefore, the calculated mass of Cy7.5AA in the solution is 8.9 μg.

To determine the mass of CDP in our test solution, we use the following formula

$$m_{\mathrm{CDP}} = m_t - m_{\mathrm{Cy7.5AA}}$$

In this formula, m_t denotes the total mass of the solid added to prepare the solution (1.0 mg), and $m_{\mathrm{Cy7.5AA}}$ is the mass of Cy7.5AA obtained from previous calculations (8.9 μg). Consequently, the calculated mass of CDP in the solution is 991.1 μg.

The concentration of CDP in the solution can be calculated using the formula

$$c_{\mathrm{CDP}} = \frac{m_{\mathrm{CDP}}}{M_{\mathrm{CDP}} \times v}$$

Here, m_{CDP} represents the mass of CDP in the solution (991.1 μg), v is the volume of the solution (1.0 ml), and M_{CDP} is the average molar mass of CDP determined by gel penetration chromatography (90,331 g mol^{-1}). On the basis of this calculation, the concentration of CDP in the solution is found to be 10.9 μM.

To estimate the average number of Cy7.5AA molecules that can bind to each CDP in the test solution, we assume that each binding site on Cy7.5AA has an equal and independent chance of binding with any CD unit on a CDP. Under this assumption, the calculation uses the following formula

$$\text{Average number of Cy7.5AA per CDP} = \frac{c_{\mathrm{Cy7.5AA}} \times v \times \text{Binding sites per Cy7.5AA}}{c_{\mathrm{CDP}} \times v}$$

Given that each Cy7.5AA molecule has four binding sites for a CD unit, the average number of Cy7.5AA molecules that can bind to each CDP is ~3.45. This value closely matches the theoretical estimate of 3.24, calculated based on sample preparation parameters. While this value might seem modest, it not only confirms the precision and effectiveness of our synthetic methodology but is also crucial for ensuring the stability and functional integrity of the supramolecular probes in vivo, where adaptation to the complex and dynamic nature of biological environments is essential.

The loading capacity (LC) of Cy7.5AA@CDP is calculated using the formula

$$\mathrm{LC} = \frac{m_{\mathrm{Cy7.5AA}}}{m_t} \times 100$$

where m_t is the total mass of the solid used in the preparation (1.0 mg), and $m_{\mathrm{Cy7.5AA}}$ is the mass of Cy7.5AA as previously determined (8.9 μg). The calculated LC of Cy7.5AA@CDP is 0.89%, which closely aligns with the theoretical value of 0.83%, a figure derived from the parameters set during the sample preparation process.

Serum protein binding test

To assess the impact of supramolecular assembly on protein interactions with the probes, Cy7.5AA@CDP, ICG, Cl-Cy7AA@CDP, and Cl-Cy7COOH were incubated with human serum albumin (HSA) under physiological conditions at 37°C for 0 to 5 hours, followed by evaluation of their ultraviolet absorption spectra.

Preparation of PBS solutions and acidified/alkaline saline solutions with phosphate at different pH values
Commercially available PBS powder was dissolved in Milli-Q water to obtain a solution with a pH range of 7.2 to 7.4. To adjust the pH within the range of 6.0 to 8.0, HCl or NaOH was added to the PBS solution, with continuous stirring and monitoring using a pH meter until the desired pH was achieved. For more acidic or alkaline solutions, additional HCl or NaOH was added to obtain acidified or alkaline saline solutions with phosphate. Although these resulting solutions can no longer be considered standard PBS due to the overwhelmed buffering capacity of the phosphate components, they were used for comparison because they maintain similar ion types and concentrations compared to PBS.

Photostability assay
Solutions of Cy7.5AA@CDP and ICG (both at 10 μM) in PBS (10 mM, pH 7.4) were prepared, and their absorption spectra were recorded. The solutions were subjected to continuous light irradiation at 808 nm with a power density of 110 mW/cm^2. Absorbance of the solutions was recorded every 5 min. The absorbance of the fluorophores (788 nm for Cy7.5AA@CDP and 778 nm for ICG) was plotted against irradiation time.

Cytotoxicity
CT26, HK-2, or LX-2 cells were obtained from Wuhan Pricella Biotechnology Co. Ltd. The cells were plated in 96-well flat-bottom plates at 1×10^5 cells per well and allowed to grow overnight prior to exposure to the probes. Then, MTT (3-(4,5-dimethylthiazol-2-yl)-2,5 diphenyl tetrazolium bromide) reagent (0.5 mg/ml) was added for 4 hours at 37°C and DMSO (100 μl per well) was further incubated with cells to dissolve the precipitated formazan violet crystals at 37°C for 15 min. The absorbance was measured at 490 nm by a multidetector microplate reader. The following formula was used to calculate the viability of cell growth: Cell viability (%) = (mean of absorbance of treatment group/mean of absorbance of control) × 100.

Cellular uptake studies
AML-12 and RAW264.7 were obtained from Wuhan Pricella Biotechnology Co. Ltd. The cells were cultured in high-glucose Dulbecco's modified Eagle's medium (Hyclone) supplemented with 10% fetal bovine serum (BI) and 1% antibiotics [penicillin (100 U/ml) and streptomycin (100 μg/ml), Hyclone] at 37°C and 5% CO_2. Before imaging, the cells were washed three times with PBS. Microscopic imaging was performed using a Nikon confocal microscope equipped with a 640-nm excitation filter, and fluorescence was collected in the red channel within the wavelength range of 663 to 738 nm.

Blood biochemistry and hematology analysis
Healthy BALB/c mice ($n = 3$) received intravenous injections of either saline or Cy7.5AA@CDP (100 μM, 100 μl) and were observed for 24, 48, or 72 hours. Blood samples were collected following standard protocols and sent to Wuhan Servicebio Technology Co. Ltd. for analysis.

In vivo fluorescence imaging
All animal procedures were performed in accordance with license nos. SYXK (Xiang) 2018-0006 and SYXK (Xiang) 2023-0010 approved by the Laboratory Animal Center of Hunan, and experiments were approved by the Animal Ethics Committee of the College of Biology (Hunan University). The in vivo (living mice) imaging was carried out using an IVIS Lumina XR (IS1241N6071), with an excitation wavelength of 745 nm and an emission band at 820 to 880 nm. The imaging SBR was calculated as SBR = fluorescence/background. For tumor imaging, the background was determined by selecting an area contralateral to the tumor (normal tissue). Specifically, in the subcutaneous tumor models where tumors were implanted on the upper side of the right hind leg, the background was consistently selected from an area of the same size on the upper side of the left hind leg. For the orthotopic breast cancer model, where cells were injected into the fat pads beneath the fourth pair of mammary glands on the left side, an area of the same size on the right side was used as the background. For renal imaging, the background was consistently chosen from an area of the same size as the kidneys, located on the upper side of the hind legs of the mice. In the capillary depth experiments, the background signal was calculated as the average signal from the left and right sides of the capillary.

In vivo renal clearance kinetics and stability studies
BALB/c mice were intravenously injected with ICG or Cy7.5AA@CDP and then placed in metabolism cages. The separated mouse urine from ICG or Cy7.5AA@CDP was collected at 24 hours after injection. Subsequently, the urine was quantified based on absorption, and each probe's standard curve was constructed in control urine.

To determine the in vivo stability of Cy7.5AA@CDP, the absorption spectra of Cy7.5AA@CDP collected from urine were measured and compared with its pure form in PBS. In addition, after 24 hours of injection of the probe Cy7.5AA@CDP, urine was collected and tested for mass spectra, and the molecular weight of Cy7.5AA was determined following the addition of an excess amount of amantadine (5 mM) that release the free Cy7.5AA molecule by the formation of complex amantadine@CDP.

Pharmacokinetics studies
Pharmacokinetics studies were performed in accordance with previous methods (*6*). Mice were anesthetized using a rodent ventilator at 2 liters/min of air mixed with 4% isoflurane for the duration of the experiment. The end of the tail was cut for blood extraction, and the blood was sampled in heparinized capillary tubes as a reference before injection. Mice were intravenously injected with probes (ICG or Cy7.5AA@CDP), and the blood was sampled at different times thereafter.

For analysis using the fluorescence emission of the probes, the collected blood was placed in an anticoagulant solution, and the NIR-II fluorescence images of the blood samples were collected using a home-built small animal imaging system with a 640- × 512-pixel 2D InGaAs NIRvana CCD camera. The initial fluorescence intensity after probe injection was taken as the 100% intensity value, and the time point corresponding to half of the initial intensity value was considered as the in vivo half-life of the probe. Images were processed using the LightField imaging software and ImageJ. The excitation wavelength was 808 nm, and the emission band was at 1040 to 1700 nm.

For HPLC analysis, the collected blood samples were stored in an ice box to prevent clotting. An amantadine solution was added to release Cy7.5AA molecules from Cy7.5AA@CDP before centrifugation at 4500 rpm for 15 min. The concentrations of Cy7.5AA in the blood were then analyzed using HPLC. The HPLC analysis was performed by measuring the peak area for Cy7.5AA at a detection wavelength of 700 nm. The initial HPLC peak area after probe injection

was taken as the reference (100% concentration), and the subsequent peak areas were plotted as a function of time to calculate the elimination half-life.

Renal clearance studies

Mice were intravenously injected with Cy7.5AA@CDP or ICG (100 μM, 100 μl) and placed in metabolic cages ($n = 3$).

For analysis based on the absorbance of the probes, the urine was collected 24 hours after injection, and the absorbance of the urine were recorded.

For HPLC analysis, urine and feces were collected 24 hours after injection, diluted in an amantadine solution in ethanol, centrifuged at 4500 rpm for 10 min, and filtered using a 0.22-μm syringe filter. The resulting solution was analyzed using HPLC. The renal clearance rate of Cy7.5AA was calculated based on the standard curve of Cy7.5AA established in a control ethanol/saline solution. Mice were euthanized, and major organs were collected and homogenized in a 1:1 buffer solution [containing 10 mM PBS (pH 7.4) and 50% ethanol solution of amantadine]. The homogenates were centrifuged at 4500 rpm for 15 min to remove insoluble components. The supernatant containing the extracted molecules was analyzed using HPLC. Cy7.5AA was quantified based on the standard curve of Cy7.5AA established in a control ethanol/saline solution.

Histopathologic studies and serum kidney function assay

All tissues of bare BALB/c mice were immediately fixed in 10% formaldehyde immediately after resection. Histological examination was performed by Wuhan Servicebio Technology Co. Ltd. according to a conventional method and then applied for hematoxylin and eosin (H&E) staining.

For the serum biochemistry test, mouse serum was collected at $t = 6$, 12, 24, 48, and 72 hours after treatment with cisplatin (20 mg/kg) or saline. Creatinine and BUN levels were assessed using commercial kits, following the manufacturer's protocol. Three mice were analyzed per sample.

Fluorescent immunohistochemistry of kidney tissue

For immunofluorescence analysis, mice were intravenously injected with Cy5AA@CDP. Fluorescent Alexa Fluor 568 phalloidin was used for staining the filamentous actin, prevalent in the glomeruli and the brush border.

Animal model preparation

All animal procedures were performed in accordance with the Guidelines for Care and Use of Laboratory Animals of Hunan University, and all animal experiments were approved by the Animal Ethics Committee of the College of Biology (Hunan University). Female BALB/c mice (18 to 20 g) were purchased from Hunan SJA Laboratory Animal Co., Ltd., China.

For the establishment of drug-induced AKI models, mice were randomly selected and treated with different doses of cisplatin (10, 15, and 20 mg/kg) or at different times (6, 12, and 24 hours). The control groups were treated with saline (0.2 ml).

For the establishment of subcutaneous tumor models, 50 μl of CT-26 cells (2×10^6) was subcutaneously implanted on the right side of the back of BALB/c female mice.

For the establishment of orthotopic breast tumor models, 50 μl of the luciferase-expressing 4T1 (4T1-Luc) cells (1×10^6) was injected into the fourth pair of mammary fat pads at the base of the nipple followed by continued tracking and analysis of tumor growth. One week after implantation, these mice were used for in vivo imaging.

Urinalysis of kidney injured mice

Online urinalysis: Urine samples were collected from living mice that were intravenously injected Cy7.5AA@CDP (100 μM, 100 μl) at $t = 6$, 12, and 24 hours after treatment with cisplatin (20 mg/kg) or saline-treated mice. The collected urine samples were centrifuged at 5000 rpm for 5 min. Fluorescence images were acquired using the IVIS Lumina XR (IS1241N6071). The excitation wavelength was 745 nm. The emission band was at 820 to 880 nm.

Statistics and reproducibility

Data were reported as mean values with error bars representing the SD. Differences were considered statistically significant when $P < 0.05$. Unless otherwise specified, all of the experiments were repeated at least three times with similar results to ensure reproducibility. Cell and animal imaging experiments were performed in biologically independent replicates with the n (at least three times) noted in the figure captions. Previous studies have demonstrated that a sample size of three is sufficient to detect significant differences in similar experimental setups, particularly when the effects are robust and consistent across replicates. In addition, we aimed to minimize the use of animals in accordance with ethical guidelines, ensuring that we only use the minimum number of animals necessary to achieve reliable results.

Supplementary Materials

This PDF file includes:
Supplementary Text
Figs. S1 to S86
Tables S1 to S5
References

REFERENCES AND NOTES

1. H.-H. Han, H. Tian, Y. Zang, A. C. Sedgwick, J. Li, J. L. Sessler, X.-P. He, T. D. James, Small-molecule fluorescence-based probes for interrogating major organ diseases. *Chem. Soc. Rev.* **50**, 9391–9429 (2021).
2. G. Hong, A. L. Antaris, H. Dai, Near-infrared fluorophores for biomedical imaging. *Nat. Biomed. Eng.* **1**, 0010 (2017).
3. J. Huang, X. Chen, Y. Jiang, C. Zhang, S. He, H. Wang, K. Pu, Renal clearable polyfluorophore nanosensors for early diagnosis of cancer and allograft rejection. *Nat. Mater.* **21**, 598–607 (2022).
4. K. M. Tsoi, S. A. MacParland, X.-Z. Ma, V. N. Spetzler, J. Echeverri, B. Ouyang, S. M. Fadel, E. A. Sykes, N. Goldaracena, J. M. Kaths, J. B. Conneely, B. A. Alman, M. Selzner, M. A. Ostrowski, O. A. Adeyi, A. Zilman, I. D. McGilvray, W. C. W. Chan, Mechanism of hard-nanomaterial clearance by the liver. *Nat. Mater.* **15**, 1212–1221 (2016).
5. J. Shi, P. W. Kantoff, R. Wooster, O. C. Farokhzad, Cancer nanomedicine: Progress, challenges and opportunities. *Nat. Rev. Cancer* **17**, 20–37 (2017).
6. X. Jiang, B. Du, J. Zheng, Glutathione-mediated biotransformation in the liver modulates nanoparticle transport. *Nat. Nanotechnol.* **14**, 874–882 (2019).
7. J. Huang, J. Li, Y. Lyu, Q. Miao, K. Pu, Molecular optical imaging probes for early diagnosis of drug-induced acute kidney injury. *Nat. Mater.* **18**, 1133–1143 (2019).
8. H. S. Choi, S. L. Gibbs, J. H. Lee, S. H. Kim, Y. Ashitate, F. Liu, H. Hyun, G. Park, Y. Xie, S. Bae, M. Henary, J. V. Frangioni, Targeted zwitterionic near-infrared fluorophores for improved optical imaging. *Nat. Biotechnol.* **31**, 148–153 (2013).
9. M.-J. Baek, D.-T. Nguyen, D. Kim, S.-Y. Yoo, S. M. Lee, J.-Y. Lee, D.-D. Kim, Tailoring renal-clearable zwitterionic cyclodextrin for colorectal cancer-selective drug delivery. *Nat. Nanotechnol.* **18**, 945–956 (2023).
10. H. S. Choi, K. Nasr, S. Alyabyev, D. Feith, J. H. Lee, S. H. Kim, Y. Ashitate, H. Hyun, G. Patonay, L. Strekowski, M. Henary, J. V. Frangioni, Synthesis and in vivo fate of zwitterionic near-infrared fluorophores. *Angew. Chem. Int. Ed. Engl.* **50**, 6258–6263 (2011).
11. B. Du, Y. Chong, X. Jiang, M. Yu, U. G. Lo, A. Dang, Y.-A. Chen, S. Li, E. Hernandez, J. C. Lin, J.-T. Hsieh, J. Zheng, Hyperfluorescence imaging of kidney cancer enabled by renal

secretion pathway dependent efflux transport. *Angew. Chem. Int. Ed. Engl.* **60**, 351–359 (2021).
12. Y. Chen, P. Pei, Z. Lei, X. Zhang, D. Yin, F. Zhang, A promising NIR-II fluorescent sensor for peptide-mediated long-term monitoring of kidney dysfunction. *Angew. Chem. Int. Ed. Engl.* **60**, 15809–15815 (2021).
13. P. Cheng, K. Pu, Molecular imaging and disease theranostics with renal-clearable optical agents. *Nat. Rev. Mater.* **6**, 1095–1113 (2021).
14. C. Yao, Y. Chen, M. Zhao, S. Wang, B. Wu, Y. Yang, D. Yin, P. Yu, H. Zhang, F. Zhang, A bright, renal-clearable NIR-II brush macromolecular probe with long blood circulation time for kidney disease bioimaging. *Angew. Chem. Int. Ed. Engl.* **61**, e202114273 (2022).
15. J. Zhou, L. Rao, G. Yu, T. R. Cook, X. Chen, F. Huang, Supramolecular cancer nanotheranostics. *Chem. Soc. Rev.* **50**, 2839–2891 (2021).
16. J. Zhao, Z. Zhou, G. Li, P. J. Stang, X. Yan, Light-emitting self-assembled metallacages. *Natl. Sci. Rev.* **8**, nwab045 (2021).
17. S. Chagri, D. Y. W. Ng, T. Weil, Designing bioresponsive nanomaterials for intracellular self-assembly. *Nat. Rev. Chem.* **6**, 320–338 (2022).
18. A. Levin, T. A. Hakala, L. Schnaider, G. J. L. Bernardes, E. Gazit, T. P. J. Knowles, Biomimetic peptide self-assembly for functional materials. *Nat. Rev. Chem.* **4**, 615–634 (2020).
19. R. M. Williams, J. Shah, B. D. Ng, D. R. Minton, L. J. Gudas, C. Y. Park, D. A. Heller, Mesoscale nanoparticles selectively target the renal proximal tubule epithelium. *Nano Lett.* **15**, 2358–2364 (2015).
20. Y. Chen, S. Wang, F. Zhang, Near-infrared luminescence high-contrast in vivo biomedical imaging. *Nat. Rev. Bioeng.* **1**, 60–78 (2023).
21. J.-S. Guo, J.-J. Li, Z.-H. Wang, Y. Liu, Y.-X. Yue, H.-B. Li, X.-H. Zhao, Y.-J. Sun, Y.-H. Ding, F. Ding, D.-S. Guo, L. Wang, Y. Chen, Dual hypoxia-responsive supramolecular complex for cancer target therapy. *Nat. Commun.* **14**, 5634 (2023).
22. C. Chen, X. Ni, H.-W. Tian, Q. Liu, D.-S. Guo, D. Ding, Calixarene-based supramolecular AIE dots with highly inhibited nonradiative decay and intersystem crossing for ultrasensitive fluorescence image-guided cancer surgery. *Angew. Chem. Int. Ed. Engl.* **59**, 10008–10012 (2020).
23. M. Raynal, P. Ballester, A. Vidal-Ferran, P. W. N. M. van Leeuwen, Supramolecular catalysis. Part 1: Non-covalent interactions as a tool for building and modifying homogeneous catalysts. *Chem. Soc. Rev.* **43**, 1660–1733 (2014).
24. C. Fasting, C. A. Schalley, M. Weber, O. Seitz, S. Hecht, B. Koksch, J. Dernedde, C. Graf, E.-W. Knapp, R. Haag, Multivalency as a chemical organization and action principle. *Angew. Chem. Int. Ed. Engl.* **51**, 10472–10498 (2012).
25. W. Sun, S. Guo, C. Hu, J. Fan, X. Peng, Recent development of chemosensors based on cyanine platforms. *Chem. Rev.* **116**, 7768–7817 (2016).
26. V. G. Bandi, M. P. Luciano, M. Saccomano, N. L. Patel, T. S. Bischof, J. G. P. Lingg, P. T. Tsrunchev, M. N. Nix, B. Ruehle, C. Sanders, L. Riffle, C. M. Robinson, S. Difilippantonio, J. D. Kalen, U. Resch-Genger, J. Ivanic, O. T. Bruns, M. J. Schnermann, Targeted multicolor in vivo imaging over 1,000 nm enabled by nonamethine cyanines. *Nat. Methods* **19**, 353–358 (2022).
27. K. Hirose, A practical guide for the determination of binding constants. *J. Incl. Phenom. Macrocycl. Chem.* **39**, 193–209 (2001).
28. C. Dalvit, I. Gmür, P. Rößler, A. D. Gossert, Affinity measurement of strong ligands with NMR spectroscopy: Limitations and ways to overcome them. *Prog. Nucl. Magn. Reson. Spectrosc.* **138-139**, 52–69 (2023).
29. D. Shetty, J. K. Khedkar, K. M. Park, K. Kim, Can we beat the biotin–avidin pair? Cucurbit[7]uril-based ultrahigh affinity host–guest complexes and their applications. *Chem. Soc. Rev.* **44**, 8747–8761 (2015).
30. S. C. Penchala, M. R. Miller, A. Pal, J. Dong, N. R. Madadi, J. Xie, H. Joo, J. Tsai, P. Batoon, V. Samoshin, A. Franz, T. Cox, J. Miles, W. K. Chan, M. S. Park, M. M. Alhamadsheh, A biomimetic approach for enhancing the in vivo half-life of peptides. *Nat. Chem. Biol.* **11**, 793–798 (2015).
31. P. Aggarwal, J. B. Hall, C. B. McLeland, M. A. Dobrovolskaia, S. E. McNeil, Nanoparticle interaction with plasma proteins as it relates to particle biodistribution, biocompatibility and therapeutic efficacy. *Adv. Drug Deliv. Rev.* **61**, 428–437 (2009).
32. R. Bilardo, F. Traldi, A. Vdovchenko, M. Resmini, Influence of surface chemistry and morphology of nanoparticles on protein corona formation. *Wiley Interdiscip. Rev. Nanomed. Nanobiotechnol.* **14**, e1788 (2022).
33. H. Shinohara, A. Tanaka, T. Kitai, N. Yanabu, T. Inomoto, S. Satoh, E. Hatano, Y. Yamaoka, K. Hirao, Direct measurement of hepatic indocyanine green clearance with near-infrared spectroscopy: Separate evaluation of uptake and removal. *Hepatology* **23**, 137–144 (1996).
34. X. Ma, Y. Zhao, Biomedical applications of supramolecular systems based on host–guest interactions. *Chem. Rev.* **115**, 7794–7839 (2015).
35. J. Huang, C. Xie, X. Zhang, Y. Jiang, J. Li, Q. Fan, K. Pu, Renal-clearable molecular semiconductor for second near-infrared fluorescence imaging of kidney dysfunction. *Angew. Chem. Int. Ed. Engl.* **58**, 15120–15127 (2019).
36. B. Du, X. Jiang, A. Das, Q. Zhou, M. Yu, R. Jin, J. Zheng, Glomerular barrier behaves as an atomically precise bandpass filter in a sub-nanometre regime. *Nat. Nanotechnol.* **12**, 1096–1102 (2017).
37. B. Du, M. Yu, J. Zheng, Transport and interactions of nanoparticles in the kidneys. *Nat. Rev. Mater.* **3**, 358–374 (2018).
38. R. M. Williams, J. Shah, H. S. Tian, X. Chen, F. Geissmann, E. A. Jaimes, D. A. Heller, Selective nanoparticle targeting of the renal tubules. *Hypertension* **71**, 87–94 (2018).
39. W. Lee, R. B. Kim, Transporters and renal drug elimination. *Annu. Rev. Pharmacol. Toxicol.* **44**, 137–166 (2004).
40. B. Wang, X. He, Z. Zhang, Y. Zhao, W. Feng, Metabolism of nanomaterials in vivo: Blood circulation and organ clearance. *Acc. Chem. Res.* **46**, 761–769 (2013).
41. L. Ye, K.-T. Yong, L. Liu, I. Roy, R. Hu, J. Zhu, H. Cai, W.-C. Law, J. Liu, K. Wang, J. Liu, Y. Liu, Y. Hu, X. Zhang, M. T. Swihart, P. N. Prasad, A pilot study in non-human primates shows no adverse response to intravenous injection of quantum dots. *Nat. Nanotechnol.* **7**, 453–458 (2012).
42. J. A. Kellum, P. Romagnani, G. Ashuntantang, C. Ronco, A. Zarbock, H.-J. Anders, Acute kidney injury. *Nat. Rev. Dis. Primers.* **7**, 52 (2021).
43. J. A. Kellum, J. R. Prowle, Paradigms of acute kidney injury in the intensive care setting. *Nat. Rev. Nephrol.* **14**, 217–230 (2018).
44. M. Joannidis, W. Druml, L. G. Forni, A. B. J. Groeneveld, P. M. Honore, E. Hoste, M. Ostermann, H. M. Oudemans-van Straaten, M. Schetz, Prevention of acute kidney injury and protection of renal function in the intensive care unit: Update 2017. *Intensive Care Med.* **43**, 730–749 (2017).
45. M. Darmon, M. Ostermann, J. Cerda, M. A. Dimopoulos, L. Forni, E. Hoste, M. Legrand, N. Lerolle, E. Rondeau, A. Schneider, B. Souweine, M. Schetz, Diagnostic work-up and specific causes of acute kidney injury. *Intensive Care Med.* **43**, 829–840 (2017).
46. Y. Xu, H. Ma, J. Shao, J. Wu, L. Zhou, Z. Zhang, Y. Wang, Z. Huang, J. Ren, S. Liu, X. Chen, J. Han, A role for tubular necroptosis in cisplatin-induced AKI. *J. Am. Soc. Nephrol.* **26**, 2647–2658 (2015).
47. C. Tang, M. J. Livingston, R. Safirstein, Z. Dong, Cisplatin nephrotoxicity: New insights and therapeutic implications. *Nat. Rev. Nephrol.* **19**, 53–72 (2023).
48. A. Ozkok, C. L. Edelstein, Pathophysiology of cisplatin-induced acute kidney injury. *Biomed. Res. Int.* **2014**, 967826 (2014).
49. J. Weng, Y. Wang, Y. Zhang, D. Ye, An activatable near-infrared fluorescence probe for in vivo imaging of acute kidney injury by targeting phosphatidylserine and caspase-3. *J. Am. Chem. Soc.* **143**, 18294–18304 (2021).
50. D. Jiang, Z. Ge, H.-J. Im, C. G. England, D. Ni, J. Hou, L. Zhang, C. J. Kutyreff, Y. Yan, Y. Liu, S. Y. Cho, J. W. Engle, J. Shi, P. Huang, C. Fan, H. Yan, W. Cai, DNA origami nanostructures can exhibit preferential renal uptake and alleviate acute kidney injury. *Nat. Biomed. Eng.* **2**, 865–877 (2018).
51. P. Cheng, W. Chen, S. Li, S. He, Q. Miao, K. Pu, Fluoro-photoacoustic polymeric renal reporter for real-time dual imaging of acute kidney injury. *Adv. Mater.* **32**, 1908530 (2020).
52. J. Huang, Y. Lyu, J. Li, P. Cheng, Y. Jiang, K. Pu, A renal-clearable duplex optical reporter for real-time imaging of contrast-induced acute kidney injury. *Angew. Chem. Int. Ed. Engl.* **58**, 17796–17804 (2019).
53. M. Yu, J. Zhou, B. Du, X. Ning, C. Authement, L. Gandee, P. Kapur, J.-T. Hsieh, J. Zheng, Noninvasive staging of kidney dysfunction enabled by renal-clearable luminescent gold nanoparticles. *Angew. Chem. Int. Ed. Engl.* **55**, 2787–2791 (2016).
54. R. L. Siegel, K. D. Miller, A. Jemal, Cancer statistics, 2020. *CA Cancer J. Clin.* **70**, 7–30 (2020).
55. Z. Hu, C. Fang, B. Li, Z. Zhang, C. Cao, M. Cai, S. Su, X. Sun, X. Shi, C. Li, T. Zhou, Y. Zhang, C. Chi, P. He, X. Xia, Y. Chen, S. S. Gambhir, Z. Cheng, J. Tian, First-in-human liver-tumour surgery guided by multispectral fluorescence imaging in the visible and near-infrared-I/II windows. *Nat. Biomed. Eng.* **4**, 259–271 (2020).
56. H. Wang, X. Li, B. W.-C. Tse, H. Yang, C. A. Thorling, Y. Liu, M. Touraud, J. B. Chouane, X. Liu, M. S. Roberts, X. Liang, Indocyanine green-incorporating nanoparticles for cancer theranostics. *Theranostics* **8**, 1227–1242 (2018).
57. L. van Manen, H. J. M. Handgraaf, M. Diana, J. Dijkstra, T. Ishizawa, A. L. Vahrmeijer, J. S. D. Mieog, A practical guide for the use of indocyanine green and methylene blue in fluorescence-guided abdominal surgery. *J. Surg. Oncol.* **118**, 283–300 (2018).
58. A. L. Antaris, H. Chen, K. Cheng, Y. Sun, G. Hong, C. Qu, S. Diao, Z. Deng, X. Hu, B. Zhang, X. Zhang, O. K. Yaghi, Z. R. Alamparambil, X. Hong, Z. Cheng, H. Dai, A small-molecule dye for NIR-II imaging. *Nat. Mater.* **15**, 235–242 (2016).
59. E. D. Cosco, A. L. Spearman, S. Ramakrishnan, J. G. P. Lingg, M. Saccomano, M. Pengshung, B. A. Arús, K. C. Y. Wong, S. Glasl, V. Ntziachristos, M. Warmer, R. R. McLaughlin, O. T. Bruns, E. M. Sletten, Shortwave infrared polymethine fluorophores matched to excitation lasers enable non-invasive, multicolour in vivo imaging in real time. *Nat. Chem.* **12**, 1123–1130 (2020).
60. J. A. Carr, D. Franke, J. R. Caram, C. F. Perkinson, M. Saif, V. Askoxylakis, M. Datta, D. Fukumura, R. K. Jain, M. G. Bawendi, O. T. Bruns, Shortwave infrared fluorescence imaging with the clinically approved near-infrared dye indocyanine green. *Proc. Natl. Acad. Sci. U.S.A.* **115**, 4465–4470 (2018).
61. T. Kitai, T. Inomoto, M. Miwa, T. Shikayama, Fluorescence navigation with indocyanine green for detecting sentinel lymph nodes in breast cancer. *Breast Cancer* **12**, 211–215 (2005).
62. M. G. Jørgensen, A. P. Hermann, A. R. Madsen, S. Christensen, J. A. Sørensen, Indocyanine green lymphangiography is superior to clinical staging in breast cancer-related lymphedema. *Sci. Rep.* **11**, 21103 (2021).

63. B. Ouyang, W. Poon, Y.-N. Zhang, Z. P. Lin, B. R. Kingston, A. J. Tavares, Y. Zhang, J. Chen, M. S. Valic, A. M. Syed, P. MacMillan, J. Couture-Senécal, G. Zheng, W. C. W. Chan, The dose threshold for nanoparticle tumour delivery. *Nat. Mater.* **19**, 1362–1371 (2020).
64. Q. Wen, Y. Zhang, C. Li, S. Ling, X. Yang, G. Chen, Y. Yang, Q. Wang, NIR-II fluorescent self-assembled peptide nanochain for ultrasensitive detection of peritoneal metastasis. *Angew. Chem. Int. Ed. Engl.* **58**, 11001–11006 (2019).
65. D. Zhong, W. Chen, Z. Xia, R. Hu, Y. Qi, B. Zhou, W. Li, J. He, Z. Wang, Z. Zhao, D. Ding, M. Tian, B. Z. Tang, M. Zhou, Aggregation-induced emission luminogens for image-guided surgery in non-human primates. *Nat. Commun.* **12**, 6485 (2021).
66. P. Wang, Y. Fan, L. Lu, L. Liu, L. Fan, M. Zhao, Y. Xie, C. Xu, F. Zhang, NIR-II nanoprobes in-vivo assembly to improve image-guided surgery for metastatic ovarian cancer. *Nat. Commun.* **9**, 2898 (2018).
67. D. M. Mofford, G. R. Reddy, S. C. Miller, Aminoluciferins extend firefly luciferase bioluminescence into the near-infrared and can be preferred substrates over D-luciferin. *J. Am. Chem. Soc.* **136**, 13277–13282 (2014).
68. H. Langhals, A. Varja, P. Laubichler, M. Kernt, K. Eibl, C. Haritoglou, Cyanine dyes as optical contrast agents for ophthalmological surgery. *J. Med. Chem.* **54**, 3903–3925 (2011).
69. S. M. Usama, K. Burgess, Hows and whys of tumor-seeking dyes. *Acc. Chem. Res.* **54**, 2121–2131 (2021).
70. S. M. Usama, C.-M. Lin, K. Burgess, On the mechanisms of uptake of tumor-seeking cyanine dyes. *Bioconjug. Chem.* **29**, 3886–3895 (2018).
71. P. Kumar, A. Nagarajan, P. D. Uchil, Analysis of cell viability by the MTT assay. *Cold Spring Harb. Protoc.* **2018**, pdb.prot095505 (2018).
72. B. Li, L. Lu, M. Zhao, Z. Lei, F. Zhang, An efficient 1064 nm NIR-II excitation fluorescent molecular dye for deep-tissue high-resolution dynamic bioimaging. *Angew. Chem. Int. Ed. Engl.* **57**, 7483–7487 (2018).
73. T.-B. Ren, Z.-Y. Wang, Z. Xiang, P. Lu, H.-H. Lai, L. Yuan, X.-B. Zhang, W. Tan, A general strategy for development of activatable NIR-II fluorescent probes for in vivo high-contrast bioimaging. *Angew. Chem. Int. Ed. Engl.* **60**, 800–805 (2021).
74. M. Matsui, S. Ando, M. Fukushima, T. Shibata, Y. Kubota, K. Funabiki, Fluorescence properties of indolenium carbocyanine dyes in solid state. *Tetrahedron* **71**, 3528–3534 (2015).
75. E. A. Owens, H. Hyun, J. G. Tawney, H. S. Choi, M. Henary, Correlating molecular character of NIR imaging agents with tissue-specific uptake. *J. Med. Chem.* **58**, 4348–4356 (2015).
76. A. Samanta, M. Vendrell, R. Das, Y.-T. Chang, Development of photostable near-infrared cyanine dyes. *Chem. Commun.* **46**, 7406–7408 (2010).
77. C. L. Lawrence, A. O. Okoh, V. Vishwapathi, S. T. McKenna, M. E. Critchley, R. B. Smith, N-alkylated linear heptamethine polyenes as potent non-azole leads against Candida albicans fungal infections. *Bioorg. Chem.* **102**, 104070 (2020).
78. E. A. Owens, N. Bruschi, J. G. Tawney, M. Henary, A microwave-assisted and environmentally benign approach to the synthesis of near-infrared fluorescent pentamethine cyanine dyes. *Dyes Pigm.* **113**, 27–37 (2015).
79. J. Peng, A. Samanta, X. Zeng, S. Han, L. Wang, D. Su, D. T. B. Loong, N.-Y. Kang, S.-J. Park, A. H. All, W. Jiang, L. Yuan, X. Liu, Y.-T. Chang, Real-time in vivo hepatotoxicity monitoring through chromophore-conjugated photon-upconverting nanoprobes. *Angew. Chem. Int. Ed. Engl.* **56**, 4165–4169 (2017).

Acknowledgments
Funding: This work was supported by the National Science Foundation of China (22325401 and 22234003). **Author contributions:** X.-B.Z., L.Y., Q.W., and Z.Z. conceived and designed the study. Q.W. performed the probe synthesis and spectral experiments. Q.W. and B.X. performed the complexation constant determination experiments. Q.W. performed the cellular and animal in vivo experiments. L.X. and Z.L. performed the resection of tumors. Q.W. and Z.Z. contributed to the analysis and interpretation of the results. All authors provided critical feedback and helped shape the research, analysis, and manuscript. **Competing interests:** The authors declare that they have no competing interests. **Data and materials availability:** All data needed to evaluate the conclusions in the paper are present in the paper and/or the Supplementary Materials.

Submitted 19 April 2024
Accepted 16 September 2024
Published 18 October 2024
10.1126/sciadv.adp8719
