# Supplementary material for: Multivalent supramolecular fluorescent probes for accurate disease imaging
Source: Sci Adv. 2024 Oct 18;10(42):eadp8719. doi: 10.1126/sciadv.adp8719 (PMC11488570; doi:10.1126/sciadv.adp8719)
Supplement: Supplementary file 3 — Erratum published 25 April 2025: The original version of the Research Article contained errors in two main text figures and one supplemental figure. The following corrections have been implemented: • In Fig. 2, J and K, the x axes were corrected to display consistent increments. The display of datapoints in the figure has been updated following the x axes adjustments. • In Fig. 2K, the label for the yellow line has been corrected from “Cy7.5AA@HPβ” to “Cy7.5AA@HPβCD.” • In Fig. 3D, the image used for the Cy7.5AA@CDP group at the ventral position at the 10-minute postinjection time point was inadvertently duplicated from the 5-minute time point during figure assembly. The image for the 10-minute postinjection time has been corrected. • In Fig. 3D, the image for the ICG group at the ventral position at the 50-minute postinjection time was inadvertently duplicated from the 30-minute time point during figure assembly. The image for the 50-minute postinjection time point has been corrected. • In fig. S45A, the images of mice following intraperitoneal injections of cisplatin at 20 mg/kg (bottom row of the figure), inadvertently used images from a different experiment conducted under identical conditions but on a different date. These images, which also appear in the bottom row of Figure 5B, were inadvertently reused due to similarity in file structure and experimental setup. The images in the bottom row of fig. S45A have been corrected. Figures 2 and 3 have been corrected to reflect these changes in the updated PDF and HTML versions of the article and the Supplementary Materials PDF has been corrected. The original version of the Supplementary Materials is available here: [file sciadv.adp8719_sm.v1.pdf]

Science Advances
AAAS

Supplementary Materials for

Multivalent supramolecular fluorescent probes for accurate disease imaging

Qian Wu *et al.*

Corresponding author: Lin Yuan, lyuan@hnu.edu.cn; Xiao-Bing Zhang, xbzhang@hnu.edu.cn

Sci. Adv. **10**, eadp8719 (2024)
DOI: 10.1126/sciadv.adp8719

This PDF file includes:

Supplementary Text
Figs. S1 to S86
Tables S1 to S5
References

1. Spectroscopy data of Cyanine Dyes

Cl-Cy7AA: ^{1}H NMR (400 MHz, Chloroform-*d*) δ 8.34 (d, *J* = 14.1 Hz, 2H), 7.41 – 7.34 (m, 4H), 7.23 (t, *J* = 7.5 Hz, 2H), 7.16 (d, *J* = 8.0 Hz, 2H), 6.15 (d, *J* = 14.1 Hz, 2H), 5.60 (s, 2H), 4.06 (t, *J* = 7.2 Hz, 4H), 2.79 (s, 7H), 2.69 (t, *J* = 5.8 Hz, 3H), 2.23 (t, *J* = 7.0 Hz, 4H), 2.03 (s, 5H), 1.98 (s, 11H), 1.89 – 1.84 (m, 4H), 1.80 – 1.75 (m, 4H), 1.71 (s, 12H), 1.64 (s, 10H). ^{13}C NMR (101 MHz, Chloroform-*d*) δ 172.3, 171.7, 150.8, 144.6, 142.2, 141.0, 129.0, 127.6, 125.3, 122.2, 111.0, 101.1, 51.9, 49.3, 44.4, 41.4, 38.6, 36.5, 36.4, 29.5, 28.2, 26.6, 26.4, 23.1. HRMS (ESI): *m*/z calcd for $C_{60}H_{78}ClN_4O_2^+$: 921.5808, found: 921.5792.

Cl-Cy7.5AA: ^{1}H NMR (400 MHz, Methanol-*d₄*) δ 8.55 (d, *J* = 14.2 Hz, 2H), 8.30 – 8.26 (m, 2H), 8.01 (d, *J* = 3.4 Hz, 3H), 7.64 (t, *J* = 3.6 Hz, 2H), 7.50 (t, *J* = 7.5 Hz, 2H), 7.31 (s, 1H), 6.34 (d, *J* = 14.2 Hz, 2H), 4.33 (t, *J* = 6.5 Hz, 4H), 2.80 (d, *J* = 4.7 Hz, 6H), 2.17 (t, *J* = 6.6 Hz, 4H), 2.05 (s, 12H), 1.94 (s, 6H), 1.91 (s, 14H), 1.61 (d, *J* = 13.5 Hz, 15H), 1.28 (d, *J* = 3.7 Hz, 7H). ^{13}C NMR (101 MHz, Chloroform-*d*) δ 173.6, 171.8, 150.1, 143.6, 139.6, 133.8, 132.0, 131.0, 130.2, 128.1, 127.8, 127.3, 122.0, 110.8, 100.6, 51.9, 51.1, 44.5, 41.4, 36.5, 36.4, 34.7, 29.4, 27.7, 26.9, 26.4, 25.3, 23.1, 22.6. HRMS (ESI): *m*/z calcd for $C_{68}H_{82}ClN_4O_2^+$: 1021.6121, found: 1021.6101.

Cy7.5AC: ^{1}H NMR (400 MHz, Methanol-d_4) δ 8.21 (d, *J* = 8.6 Hz, 2H), 8.06 – 7.94 (m, 6H), 7.66 – 7.53 (m, 5H), 7.52 – 7.41 (m, 3H), 6.59 (t, *J* = 12.6 Hz, 2H), 6.39 – 6.29 (m, 2H), 4.21 (s, 4H), 3.20 (q, *J* = 7.3 Hz, 2H), 2.38 (t, *J* = 6.8 Hz, 2H), 2.18 (t, *J* = 6.6 Hz, 3H), 2.11 – 2.07 (m, 2H), 2.04 – 2.01 (m, 2H), 1.98 (s, 10H), 1.92 (s, 4H), 1.81 – 1.73 (m, 5H), 1.63 (s, 5H), 1.30 (t, *J* = 7.2 Hz, 4H). HRMS (ESI): *m*/z calcd for $C_{55}H_{64}N_3O_3^+$: 814.4943, found: 814.4929.

Cl-Cy7COOH: ^{1}H NMR (400 MHz, Methanol-d_4) δ 8.40 (d, *J* = 14.1 Hz, 2H), 7.49 (d, *J* = 7.3 Hz, 2H), 7.40 (t, *J* = 7.4 Hz, 2H), 7.32 (d, *J* = 7.9 Hz, 2H), 7.25 (t, *J* = 7.4 Hz, 2H), 6.28 (d, *J* = 14.1 Hz, 2H), 4.17 (t, *J* = 7.1 Hz, 4H), 2.71 (t, *J* = 5.7 Hz, 4H), 2.35 (t, *J* = 6.7 Hz, 4H), 1.97 – 1.90 (m, 2H), 1.89 – 1.80 (m, 4H), 1.70 (s, 14H), 1.25 (d, *J* = 5.2 Hz, 2H).

2. Supplementary Figures

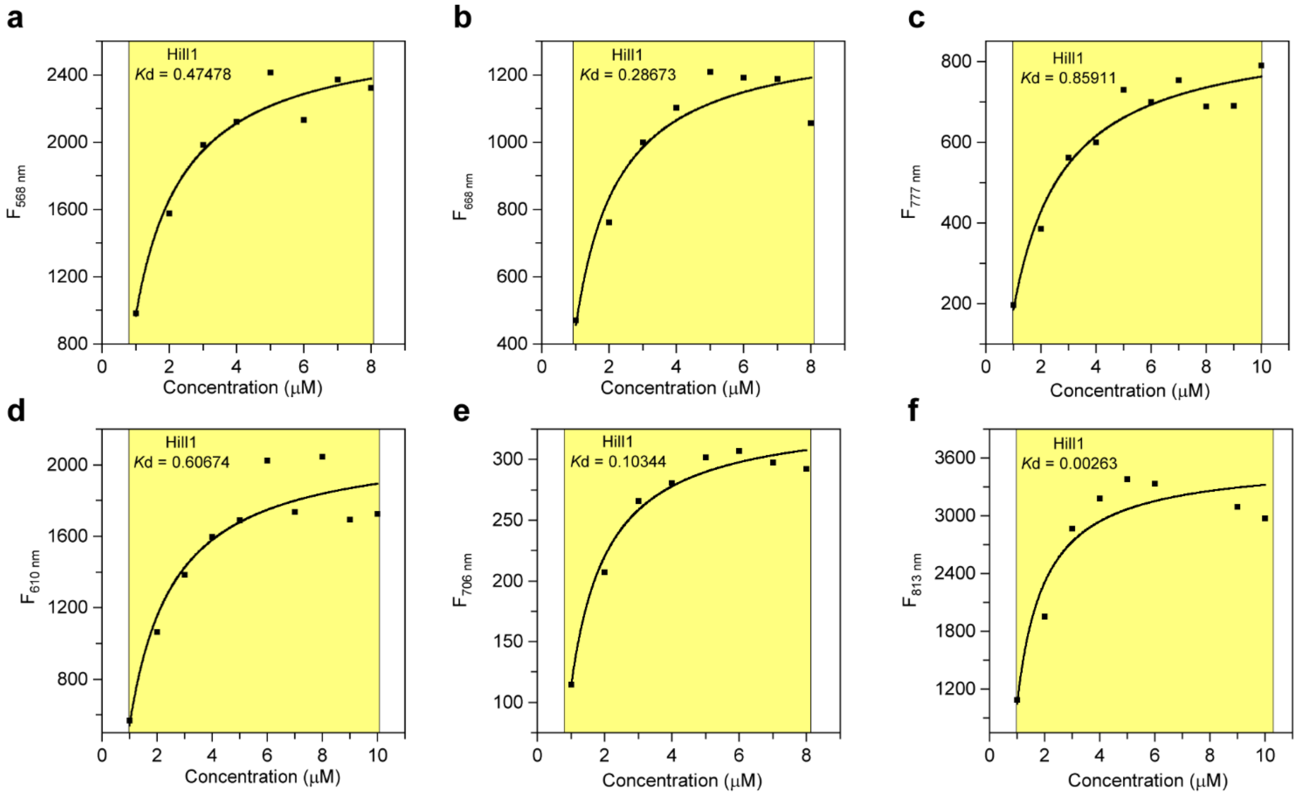

Fig. S1. Apparent association constants (K_a) between the designed cyanine dyes and β-cyclodextrin polymers (CDP). **a-f**, Fluorescence intensity changes of Cy3AA@CDP (**a**), Cy5AA@CDP (**b**), Cy7AA@CDP (**c**), Cy3.5AA@CDP (**d**), Cy5.5AA@CDP (**e**), and Cy7.5AA@CDP (**f**) in the titration experiments. For the determination of the apparent association constants (K_a), the fluorescence spectra of the supramolecular probes were first tested for different guest dye concentrations, and then the relationship between concentration and fluorescent intensity was plotted as a scatter plot. The nonlinear curve fitting function (Hill 1) in Origin2018 was chosen to fit the curve. According to the Hill 1 function equation, the dissociation constant (K_d) value is equal to the concentration value at half of the maximum fluorescence intensity, while the complexation constant is equal to $K_a = 1/K_d$.

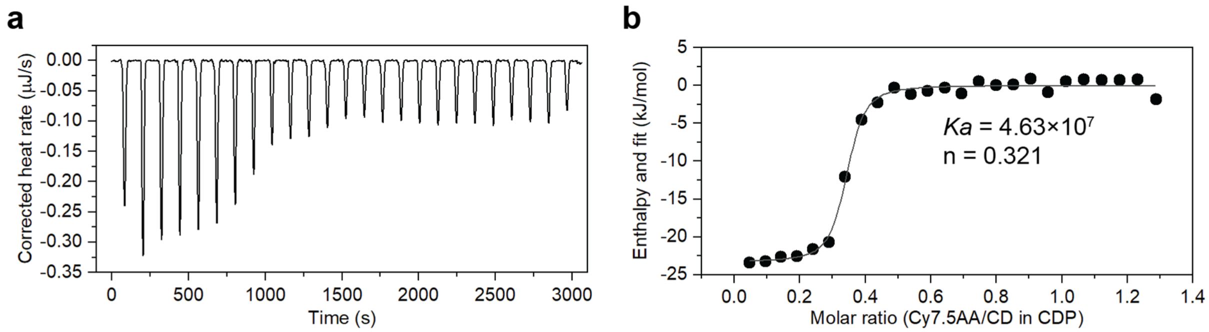

Fig. S2. Isothermal titration calorimetry (ITC) isotherms for the interaction between Cy7.5AA and CDP at 298 K. Raw heat changes observed during the titration of CDP with Cy7.5AA. Binding isotherm fitted to the raw data using independent model.

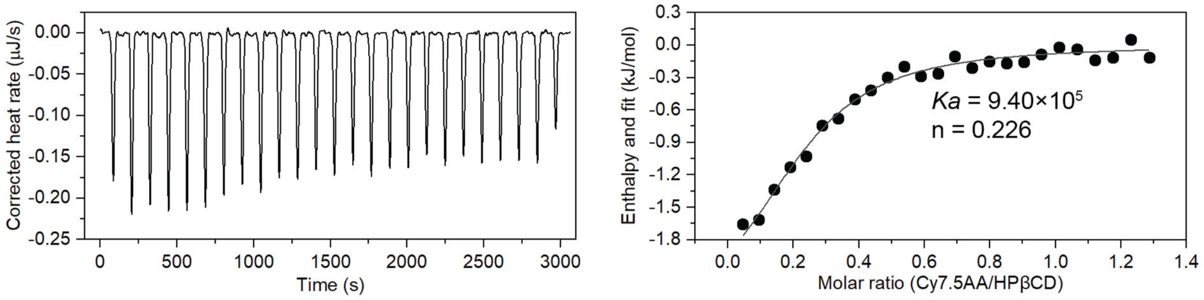

Fig. S3. Isothermal titration calorimetry (ITC) isotherms for the interaction between Cy7.5AA and HPβCD at 298 K. Raw heat changes observed during the titration of HPβCD with Cy7.5AA. Binding isotherm fitted to the raw data using independent model.

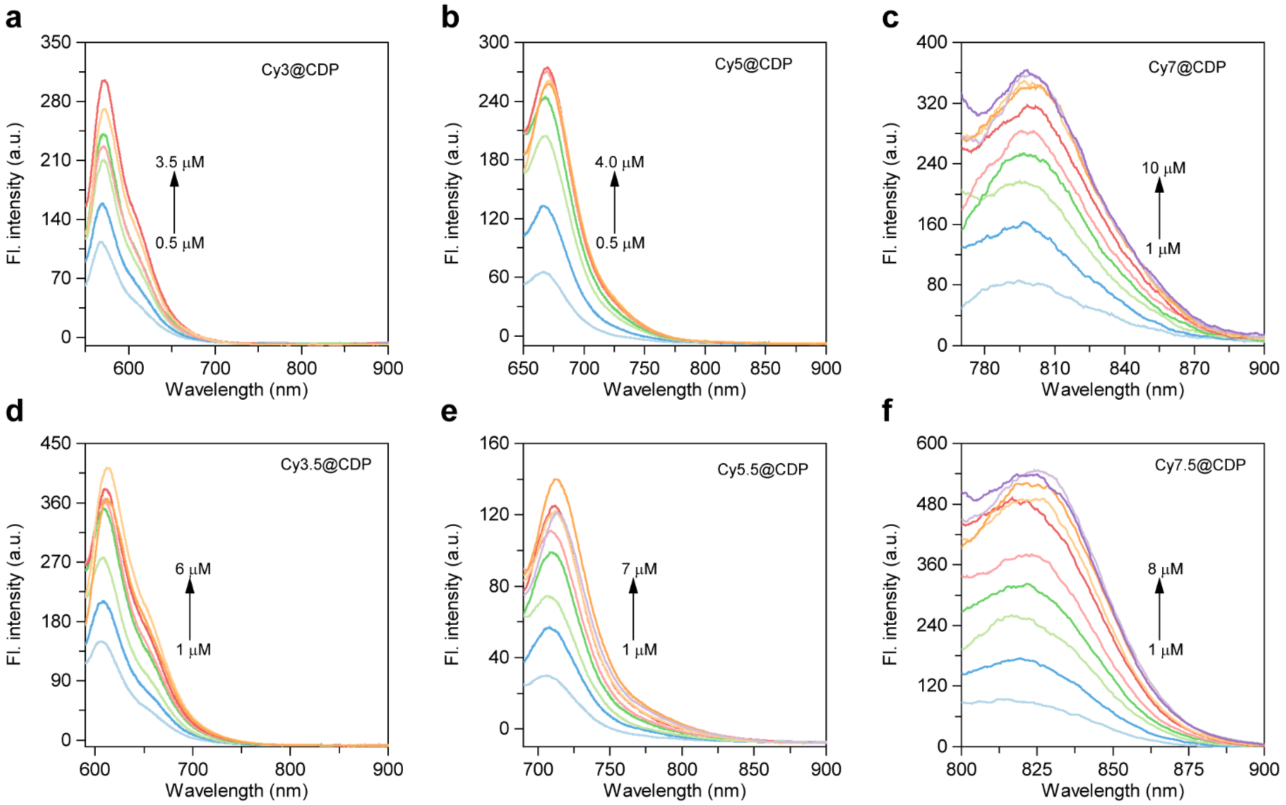

Fig. S4. Optical properties of the supramolecular probes. a-f, Concentration-dependent fluorescence emission spectra of Cy3@CDP (**a**), Cy5@CDP (**b**), Cy7@CDP (**c**), Cy3.5@CDP (**d**), Cy5.5@CDP (**e**), Cy7.5@CDP (**f**) in PBS (10 mM, pH = 7.4).

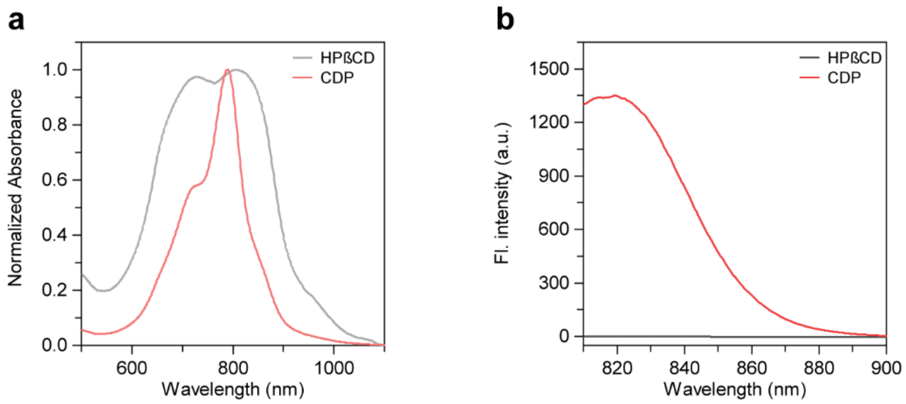

Fig. S5. Optical properties of Cy7.5AA@CDP and Cy7.5AA@HPβCD. **a-b**, Normalized absorption (**a**) and fluorescence emission spectra (**b**) of Cy7.5AA@CDP and Cy7.5AA@HPβCD in PBS (10 mM, pH = 7.4). The molar ratio for self-assembly between Cy7.5AA and HPβCD was 1:2, the concentration of Cy7.5AA was 10 μM.

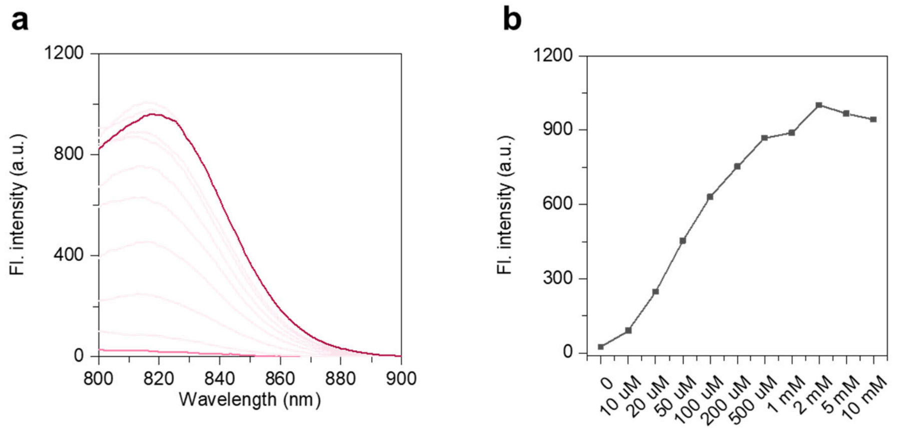

Fig. S6. Optical properties of supramolecular probe Cy7.5AA@CDP at different assembly ratios. **a**, Fluorescence emission spectra of Cy7.5AA with different concentrations of CDP in PBS (10 mM, pH=7.4). **b**, The fluorescence intensity of Cy7.5AA@CDP at 813 nm. The concentration of Cy7.5AA was 10 μM, and the concentrations of CDP were 10 μM, 20 μM, 50 μM, 100 μM, 200 μM, 500 μM, 1 mM, 2 mM, 5 mM 10 mM.

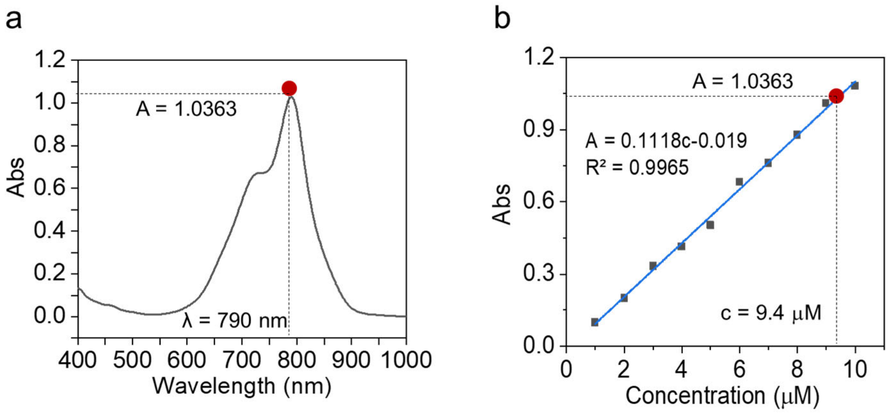

Fig. S7. Determination of loading efficiency of Cy7.5AA@CDP. a, Absorption spectra of the test solution (Cy7.5AA@CDP in PBS, 1 mg/ml). The complex exhibited absorbance of 1.0363 at 790 nm. **b,** Standard curve of Cy7.5AA@CDP in PBS. Based on the absorbance of the test solution, the concentration of Cy7.5AA in our test solution was determined to be 9.4 μM.

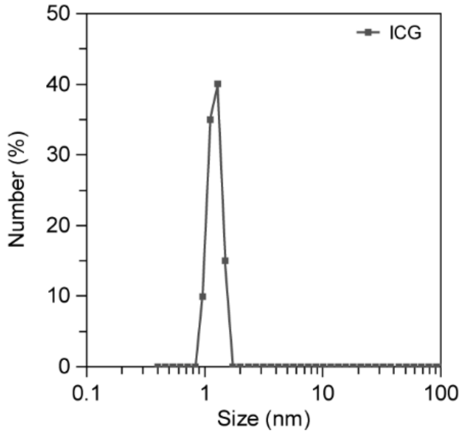

Fig. S8. Hydrodynamic diameters of indocyanine green (ICG) in PBS determined by dynamic light scattering analysis. As an analog of the cyanine dyes, the FDA-approved ICG exhibited a hydrodynamic diameter of 1.3 nm., suggesting ICG present as a small molecule in PBS.

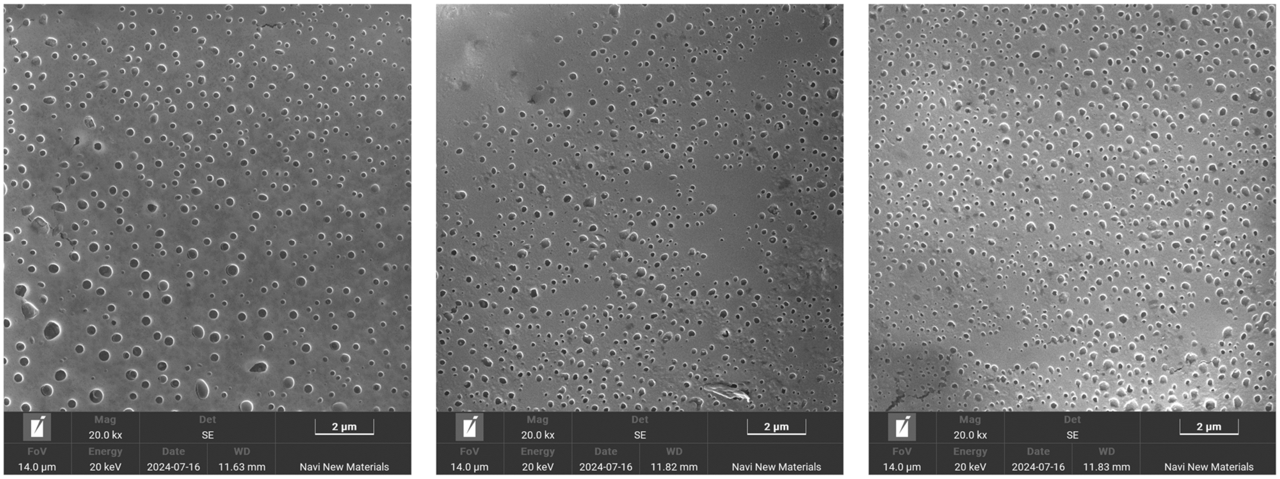

Fig. S9. Scanning electron microscopy (SEM) images of Cy7.5AA@CDP. The SEM images depict Cy7.5AA@CDP as spherical nanoparticles with diameters of 323 nm ± 29 nm (*n* = 20).

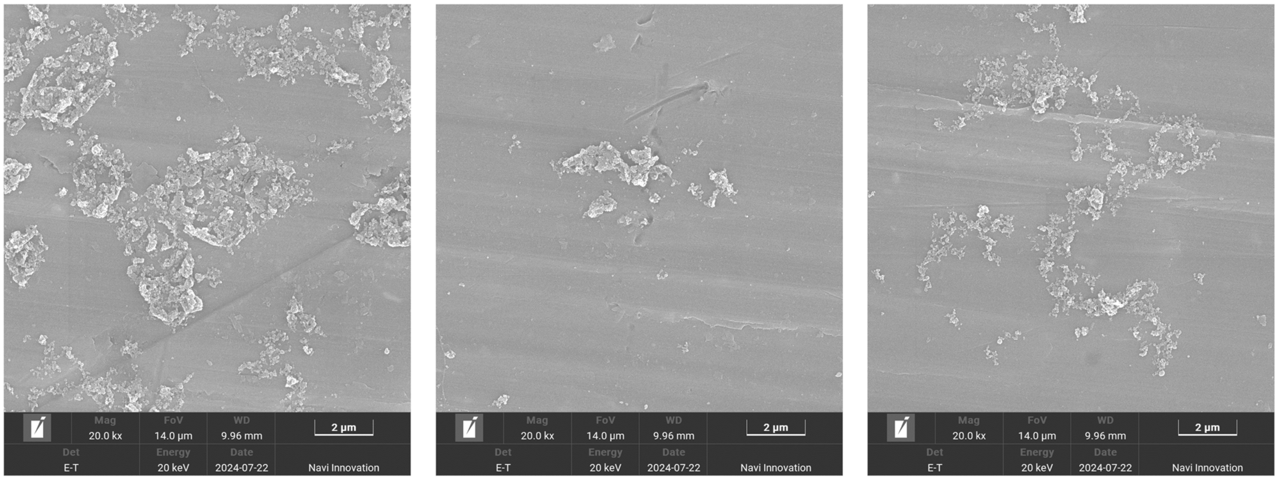

Fig. S10. Scanning electron microscopy (SEM) images of Cy7.5AA. The SEM images reveal Cy7.5AA to be an amorphous aggregate with varying sizes.

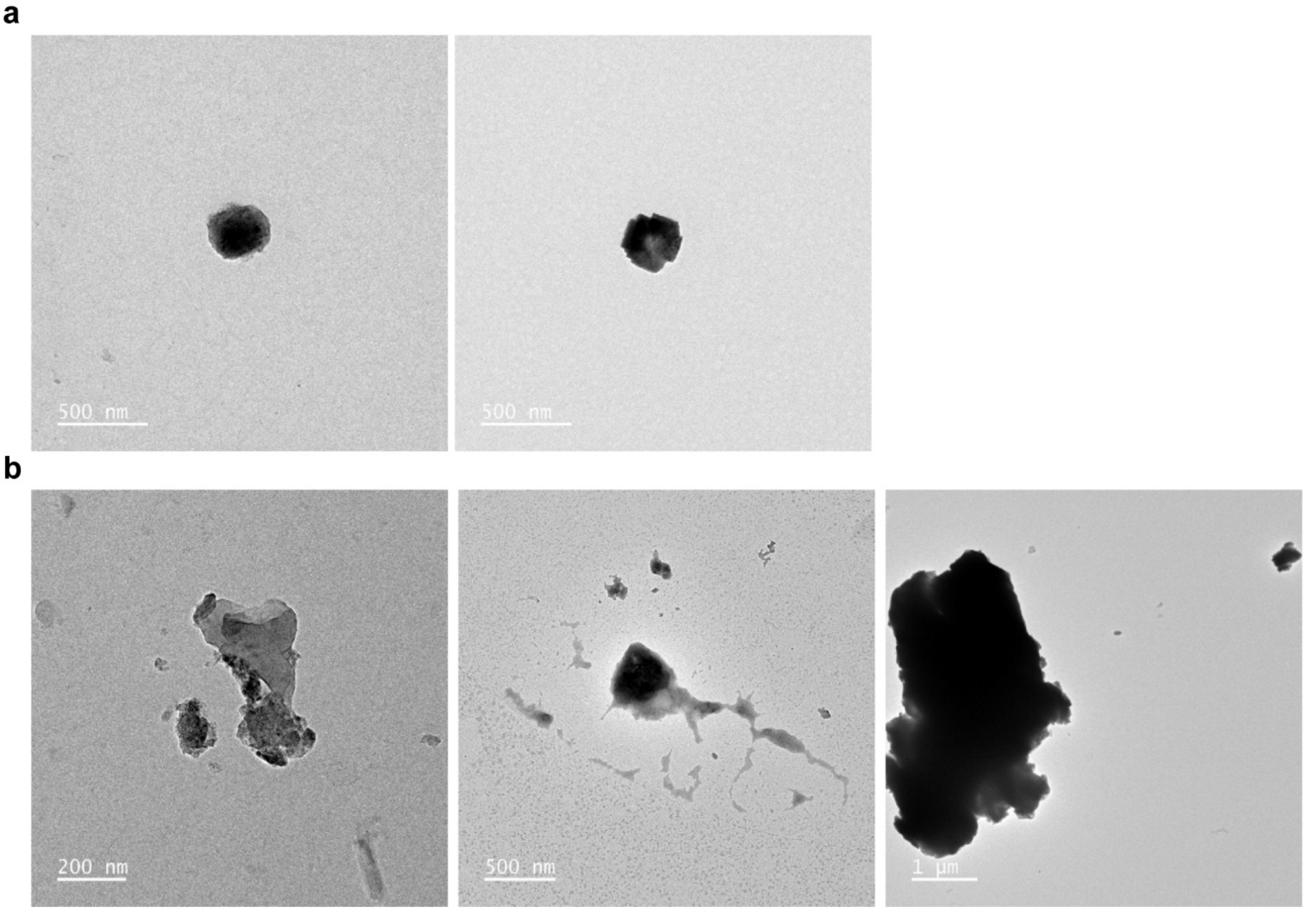

Fig. S11. Transmission electron microscopy (TEM) analysis of Cy7.5AA@CDP and Cy7.5AA. a, TEM images of Cy7.5AA@CDP, which reveal spherical nanoparticles with diameters of 365 ± 39 nm (*n* = 20). **b**, TEM images of Cy7.5AA, which reveal amorphous aggregates with varying sizes.

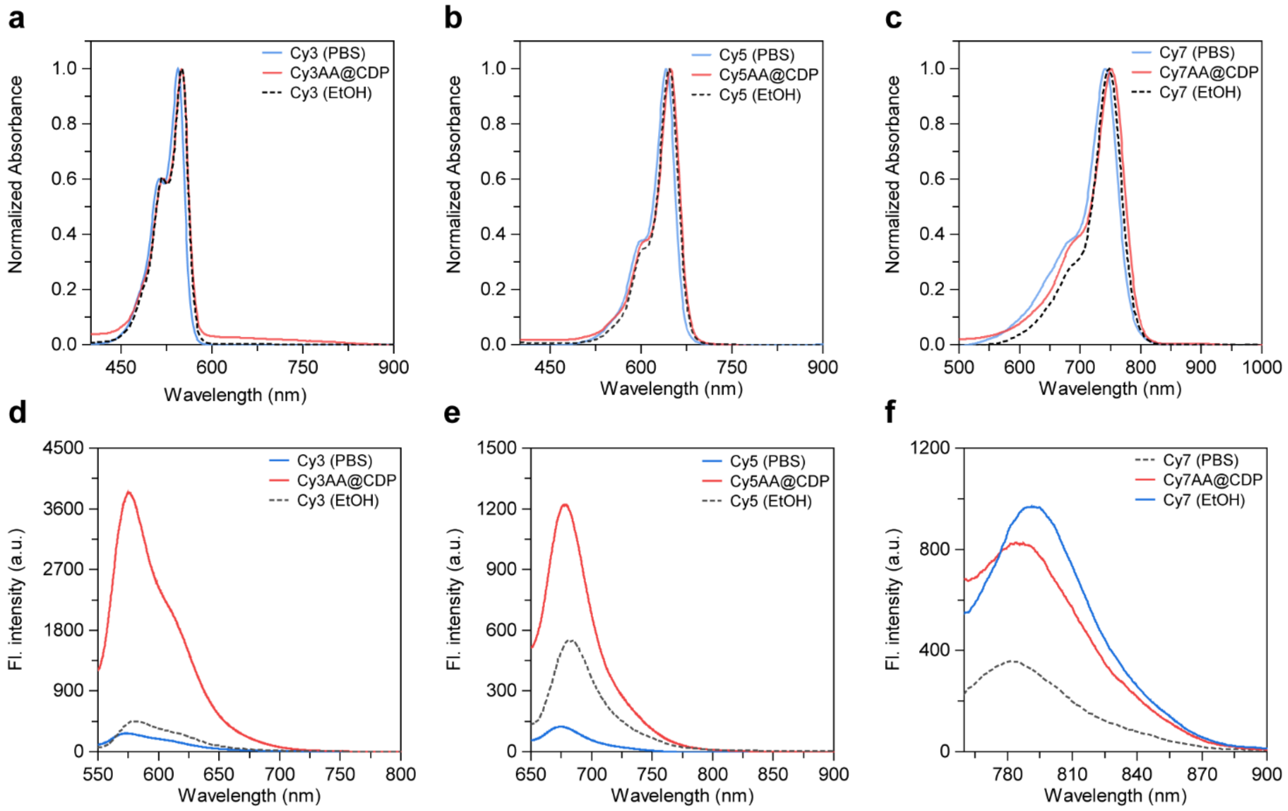

Fig. S12. Spectroscopy of small molecule and supramolecular probes in various solvents. a-c, Normalized absorption spectra of Cy3, Cy5, Cy7 (10 μM, in PBS or EtOH) and Cy3AA@CDP, Cy5AA@CDP, Cy7AA@CDP (10 μM in PBS). **d-f**, Fluorescence spectral changes of Cy3, Cy5, Cy7 (10 μM, in PBS or EtOH) and Cy3AA@CDP, Cy5AA@CDP, Cy7AA@CDP (10 μM in PBS).

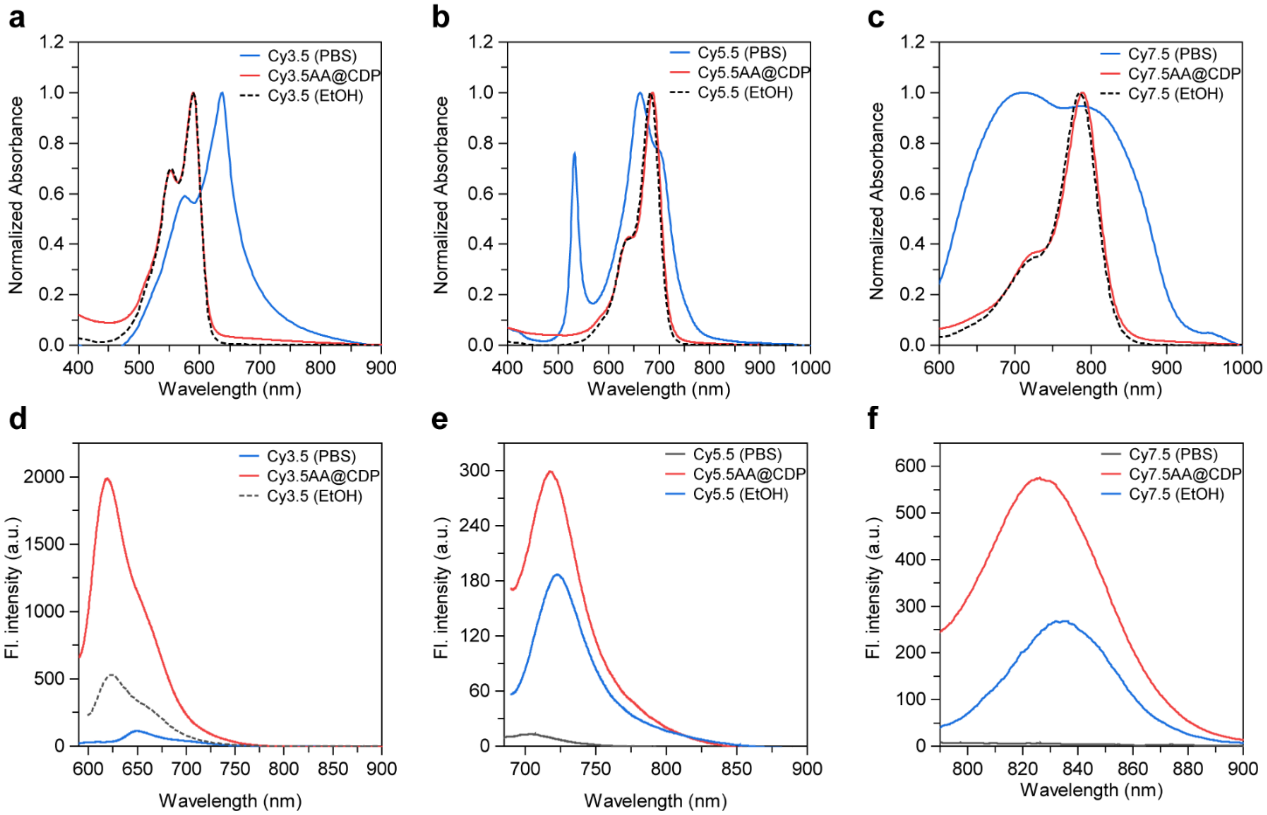

Fig. S13. Spectroscopy of small molecule and supramolecular probes in various solvents. a-c, Normalized absorption spectra of Cy3.5, Cy5.5, Cy7.5 (10 μM, in PBS or EtOH) and Cy3.5AA@CDP, Cy5.5AA@CDP, Cy7.5AA@CDP (10 μM in PBS). **d-f**, Fluorescence spectral changes of Cy3.5, Cy5.5, Cy7.5 (10 μM, in PBS or EtOH) and Cy3.5AA@CDP, Cy5.5AA@CDP, Cy7.5AA@CDP (10 μM in PBS).

a

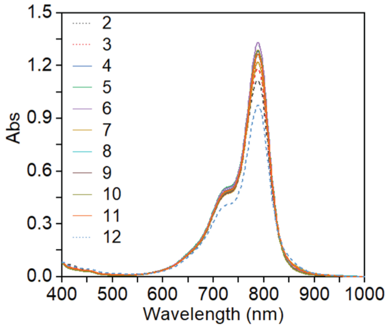

b

pH	Abs (788 nm)	Relative Abs
**2**	1.12	0.92
**3**	1.18	0.97
**4**	1.28	1.05
**5**	1.33	1.09
**6**	1.33	1.09
**7**	1.22	1.00
**8**	1.26	1.03
**9**	1.26	1.04
**10**	1.28	1.05
**11**	1.27	1.04
**12**	1.12	0.92

c

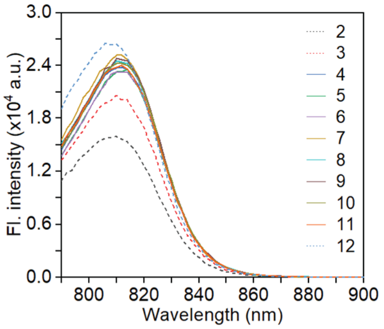

d

pH	Fl. (813 nm)	Relative Fl.
**2**	1.12	0.62
**3**	1.18	0.81
**4**	1.28	0.96
**5**	1.33	0.96
**6**	1.33	0.95
**7**	1.22	1.00
**8**	1.26	0.98
**9**	1.26	0.99
**10**	1.28	0.97
**11**	1.27	0.96
**12**	1.12	1.01

Fig. S14. Optical properties of supramolecular probe Cy7.5AA@CDP (10 μM) at PBS (10 mM) at different pH levels. a, Absorption spectra of Cy7.5AA@CDP in saline at various pH levels. **b**, Summary of the absorbance of Cy7.5AA@CDP at 788 nm across different pH levels. **c**, Fluorescence emission spectra of Cy7.5AA@CDP in saline at various pH levels. **d**, Summary of the fluorescence intensity of Cy7.5AA@CDP at 813 nm across different pH levels.

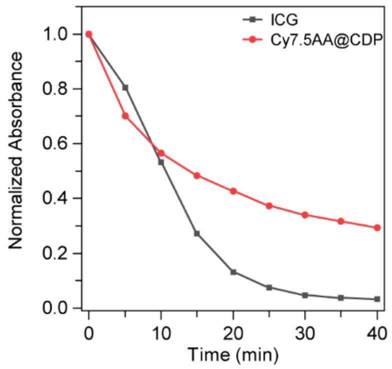

Fig. S15. Photostability of Cy7.5AA@CDP and ICG. Relative absorbance of ICG (778 nm) and Cy7.5AA@CDP (788 nm) in PBS (10 mM, pH = 7.4) with corresponding laser (808 nm) at a power density of ~110 mW/cm^2. Compared to ICG, Cy7.5AA@CDP demonstrated superior resistance to photobleaching, which indicated that cyclodextrin encapsulation improved the photostability of the fluorescence probe.

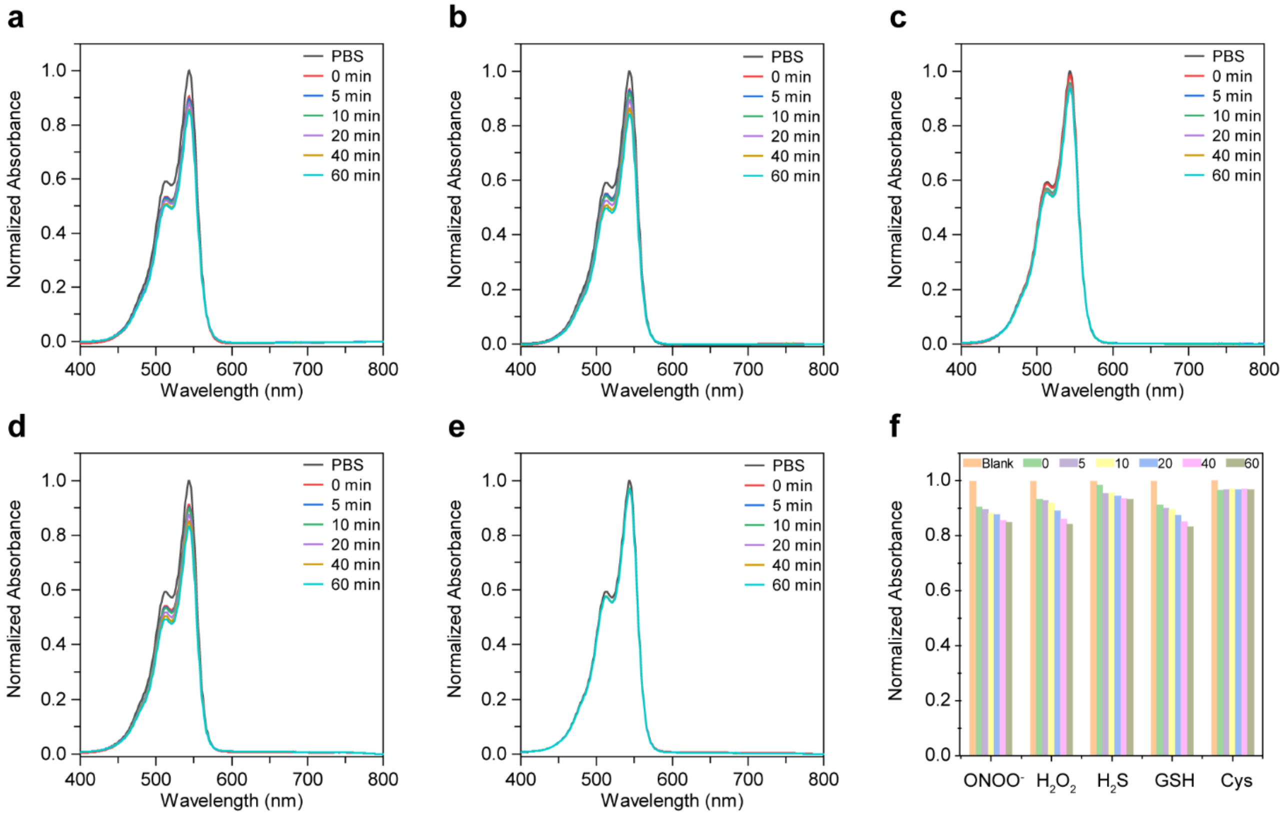

Fig. S16. Time-dependent normalized absorption spectra of Cy3 (10 μM) with various active agents in PBS (10 mM, pH = 7.4). **a**, $ONOO^-$ (5 μM); **b**, H_2O_2 (100 μM); **c**, H_2S (100 μM); **d**, GSH (1 mM); **e**, Cys (100 μM). A declining absorption profile observed in (**a-d**), indicates that Cy3 is susceptible to damage by reactive agents such as $ONOO^-$, H_2O_2, H_2S, GSH.

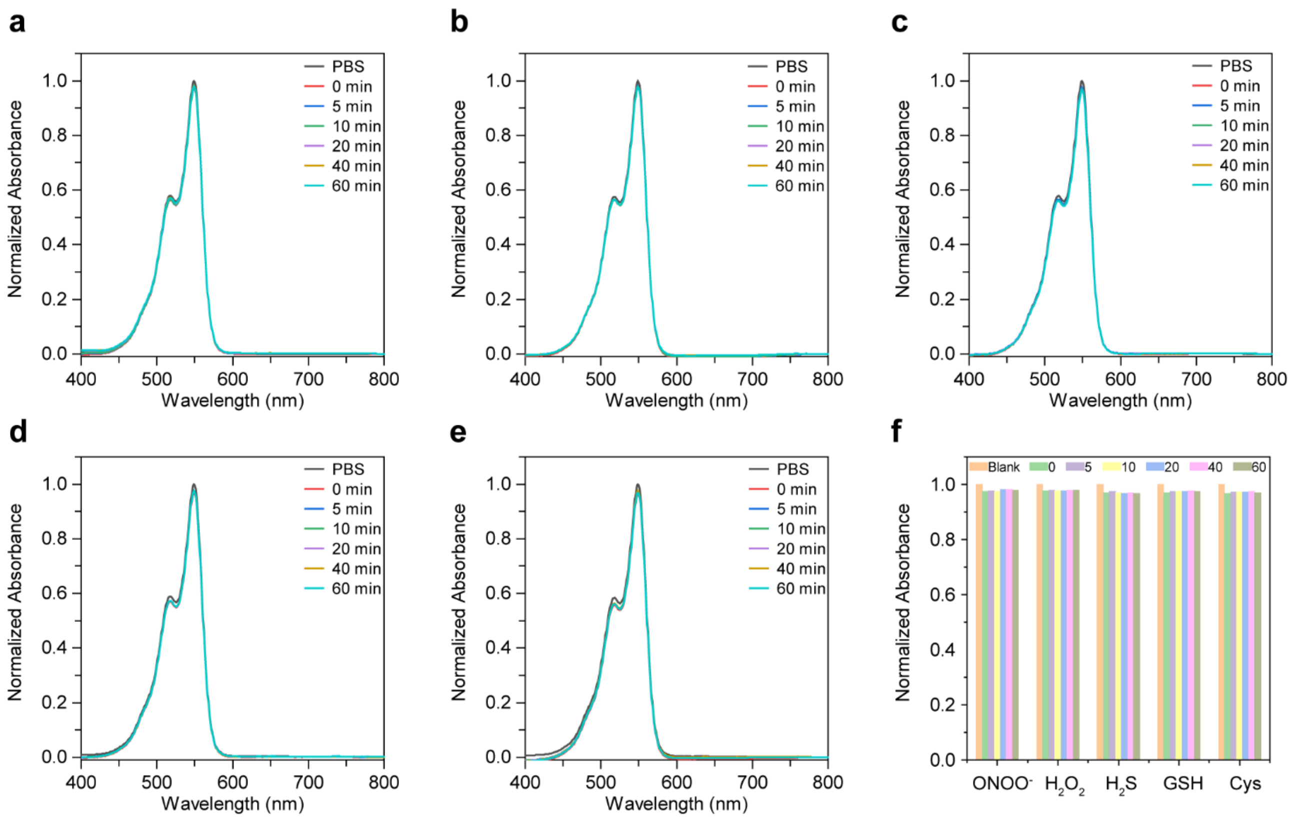

Fig. S17. Time-dependent normalized absorption spectra of Cy3AA@CDP (10 μM) with various active agents in PBS (10 mM, pH = 7.4). **a**, $ONOO^-$ (5 μM); **b**, H_2O_2 (100 μM); **c**, H_2S (100 μM); **d**, GSH (1 mM); **e**, Cys (100 μM). A consistent absorption profile with no peak shifts suggested minimal reaction between the supramolecular probe and the reactive agents.

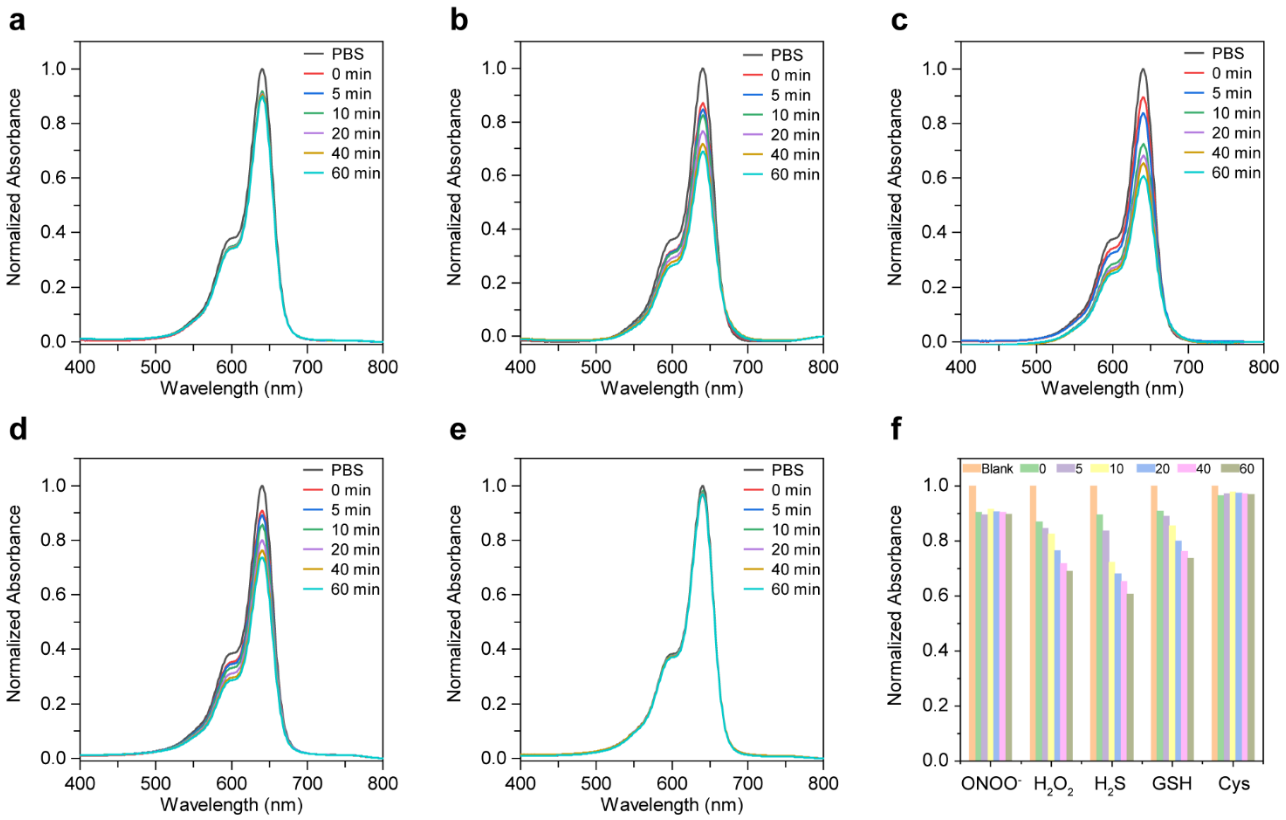

Fig. S18. Time-dependent normalized absorption spectra of Cy5 (10 μM) with various active agents in PBS (10 mM, pH = 7.4). **a**, $ONOO^-$ (5 μM); **b**, H_2O_2 (100 μM); **c**, H_2S (100 μM); **d**, GSH (1 mM); **e**, Cys (100 μM). A declining absorption profile observed in (**a-d**), indicates that Cy5 is susceptible to damage by reactive agents such as $ONOO^-$, H_2O_2, H_2S, GSH.

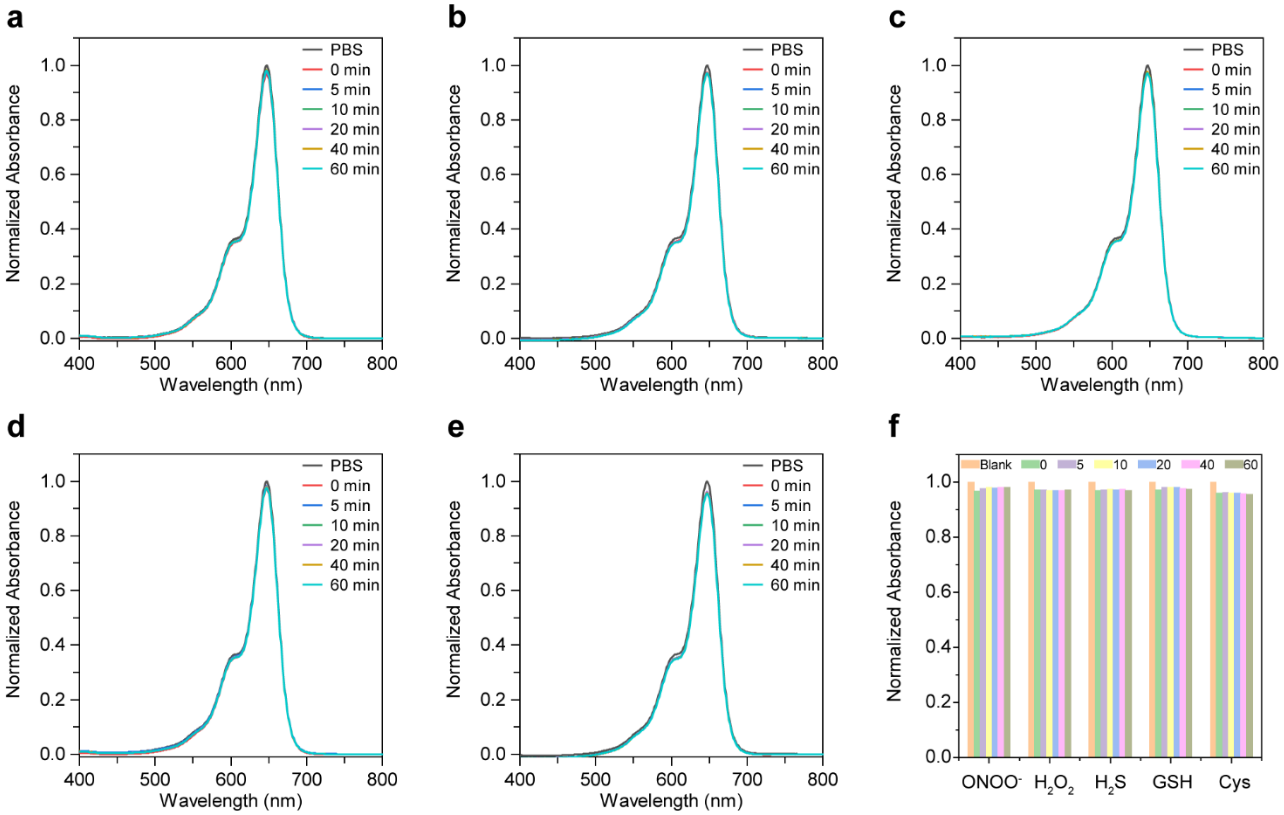

Fig. S19. Time-dependent normalized absorption spectra of Cy5AA@CDP (10 μM) with various active agents in PBS (10 mM, pH = 7.4). a, $ONOO^-$ (5 μM); **b**, H_2O_2 (100 μM); **c**, H_2S (100 μM); **d**, GSH (1 mM); **e**, Cys (100 μM). A consistent absorption profile with no peak shifts suggested minimal reaction between the supramolecular probe and the reactive agents.

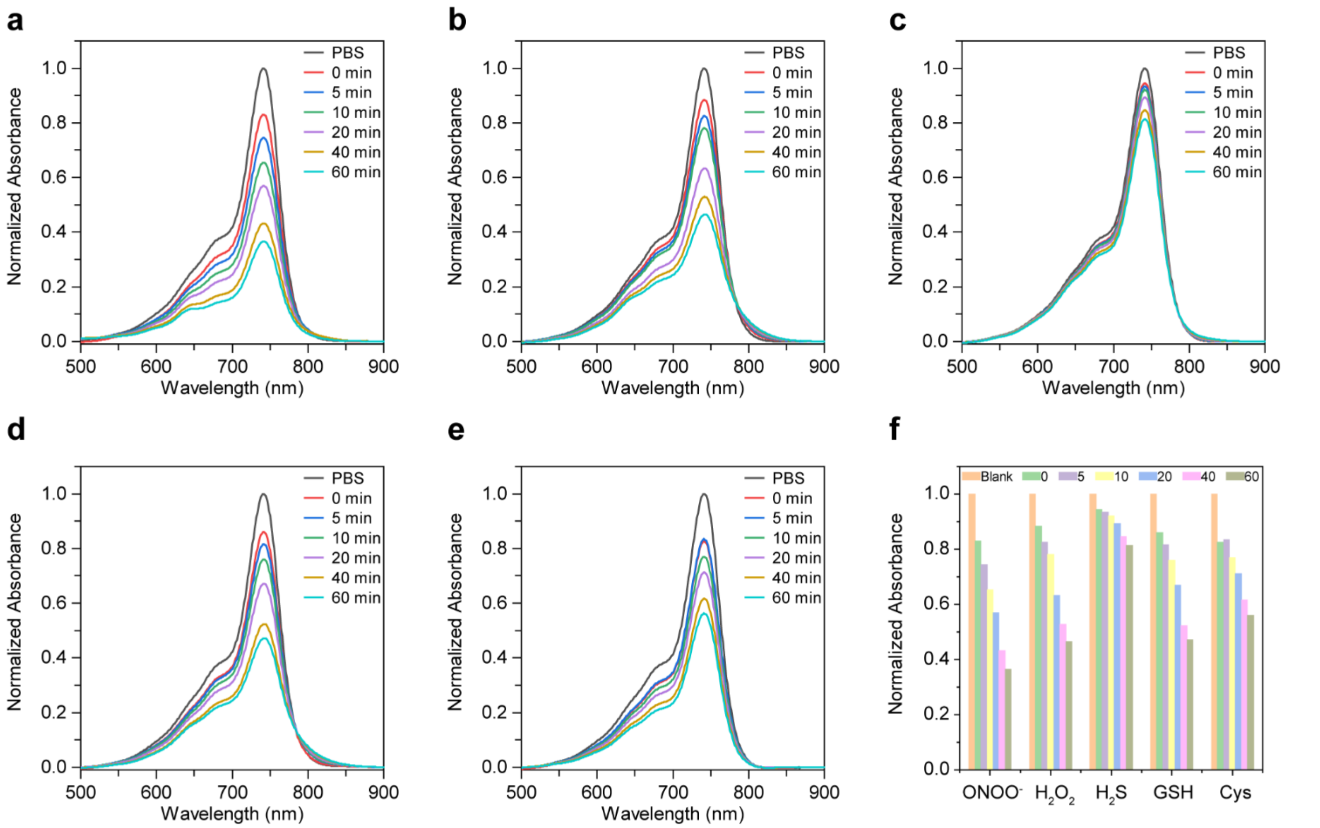

Fig. S20. Time-dependent normalized absorption spectra of Cy7 (10 µM) with various active agents in PBS (10 mM, pH = 7.4). **a**, $ONOO^-$ (5 µM); **b**, H_2O_2 (100 µM); **c**, H_2S (100 µM); **d**, GSH (1 mM); **e**, Cys (100 µM). A declining absorption profile observed in (**a-d**), indicates that Cy7 is susceptible to damage by reactive agents such as $ONOO^-$, H_2O_2, H_2S, GSH.

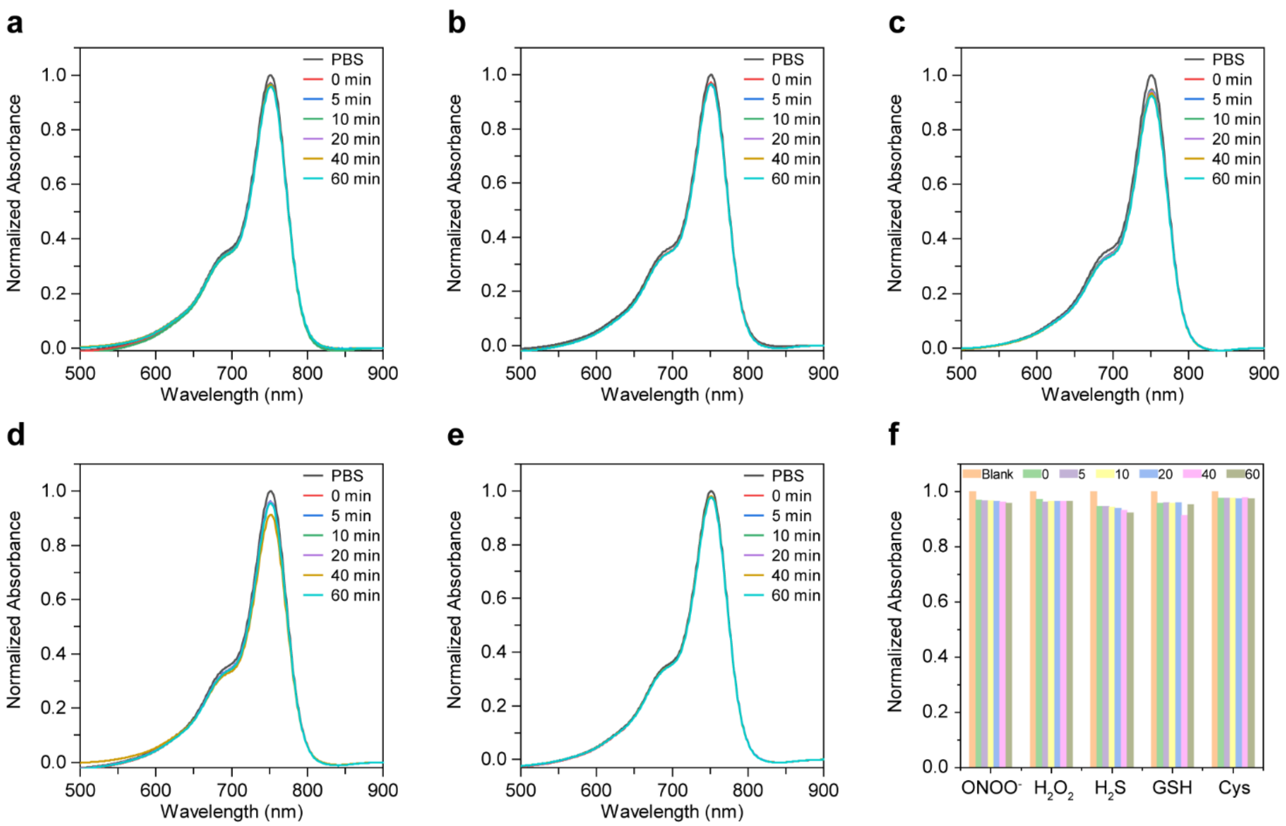

Fig. S21. Time-dependent normalized absorption spectra of Cy7AA@CDP (10 μM) with various active agents in PBS (10 mM, pH = 7.4). **a**, $ONOO^-$ (5 μM); **b**, H_2O_2 (100 μM); **c**, H_2S (100 μM); **d**, GSH (1 mM); **e**, Cys (100 μM). A consistent absorption profile with no peak shifts suggested minimal reaction between the supramolecular probe and the reactive agents.

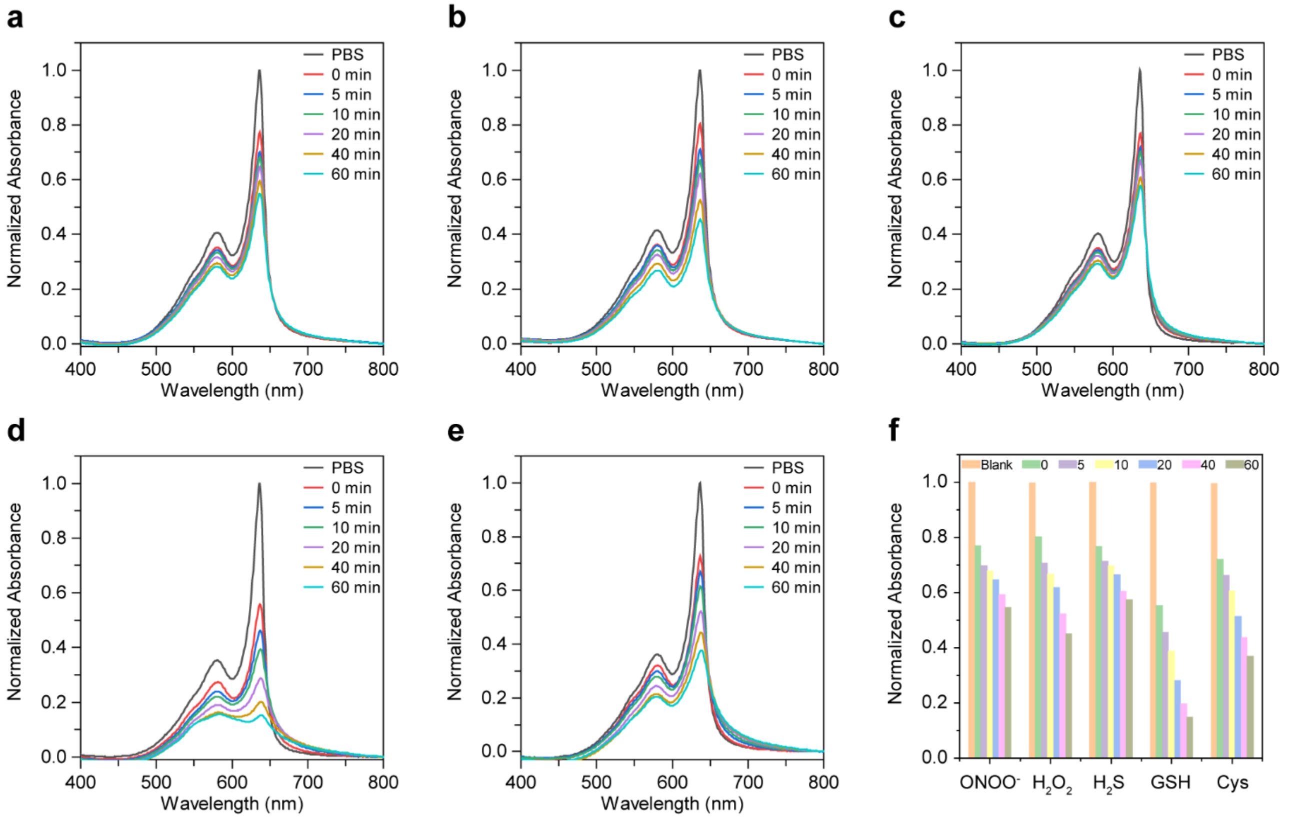

Fig. S22. Time-dependent normalized absorption spectra of Cy3.5 (10 μM) with various active agents in PBS (10 mM, pH = 7.4). a, $ONOO^-$ (5 μM); **b**, H_2O_2 (100 μM); **c**, H_2S (100 μM); **d**, GSH (1 mM); **e**, Cys (100 μM). A declining absorption profile observed in (**a-d**), indicates that Cy3.5 is susceptible to damage by reactive agents such as $ONOO^-$, H_2O_2, H_2S, GSH.

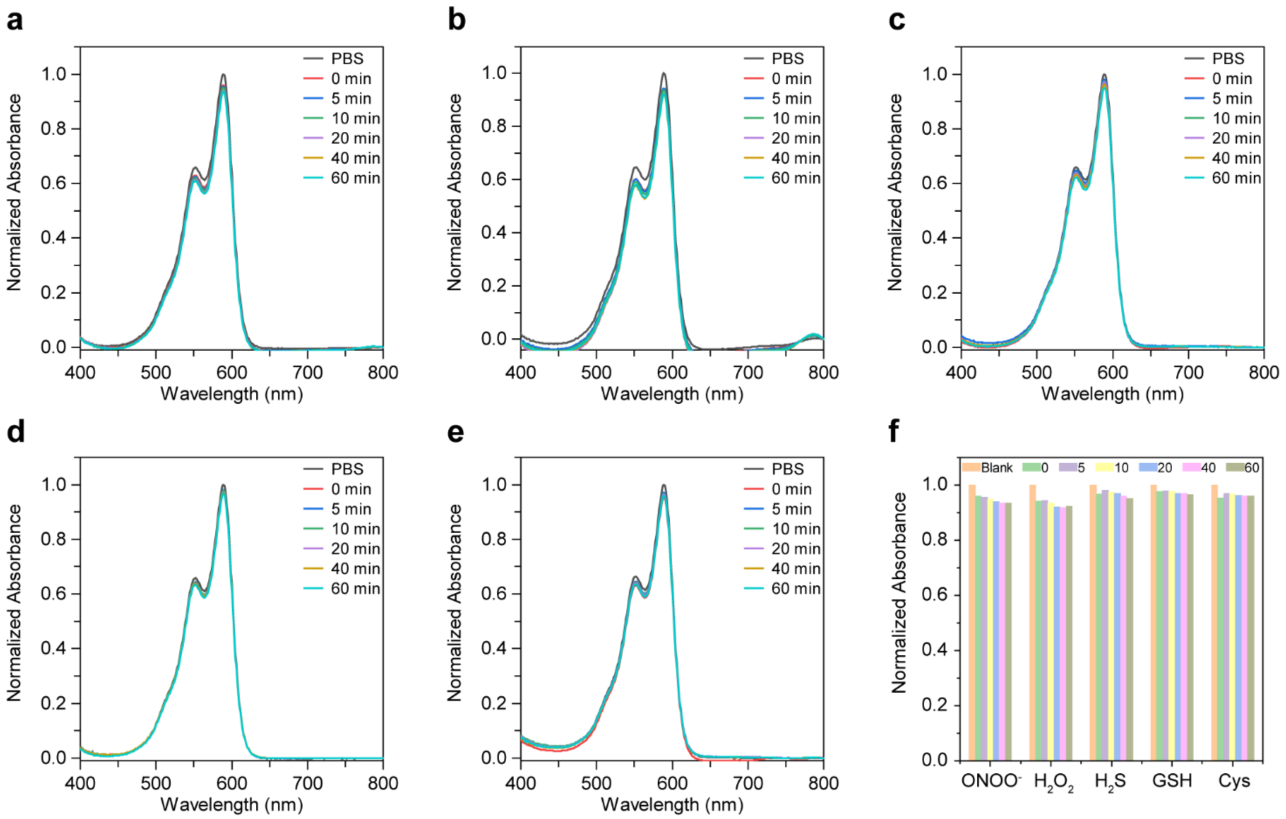

Fig. S23. Time-dependent normalized absorption spectra of Cy3.5AA@CDP (10 μM) with various active agents in PBS (10 mM, pH = 7.4). a, $ONOO^-$ (5 μM); **b**, H_2O_2 (100 μM); **c**, H_2S (100 μM); **d**, GSH (1 mM); **e**, Cys (100 μM). A consistent absorption profile with no peak shifts suggested minimal reaction between the supramolecular probe and the reactive agents.

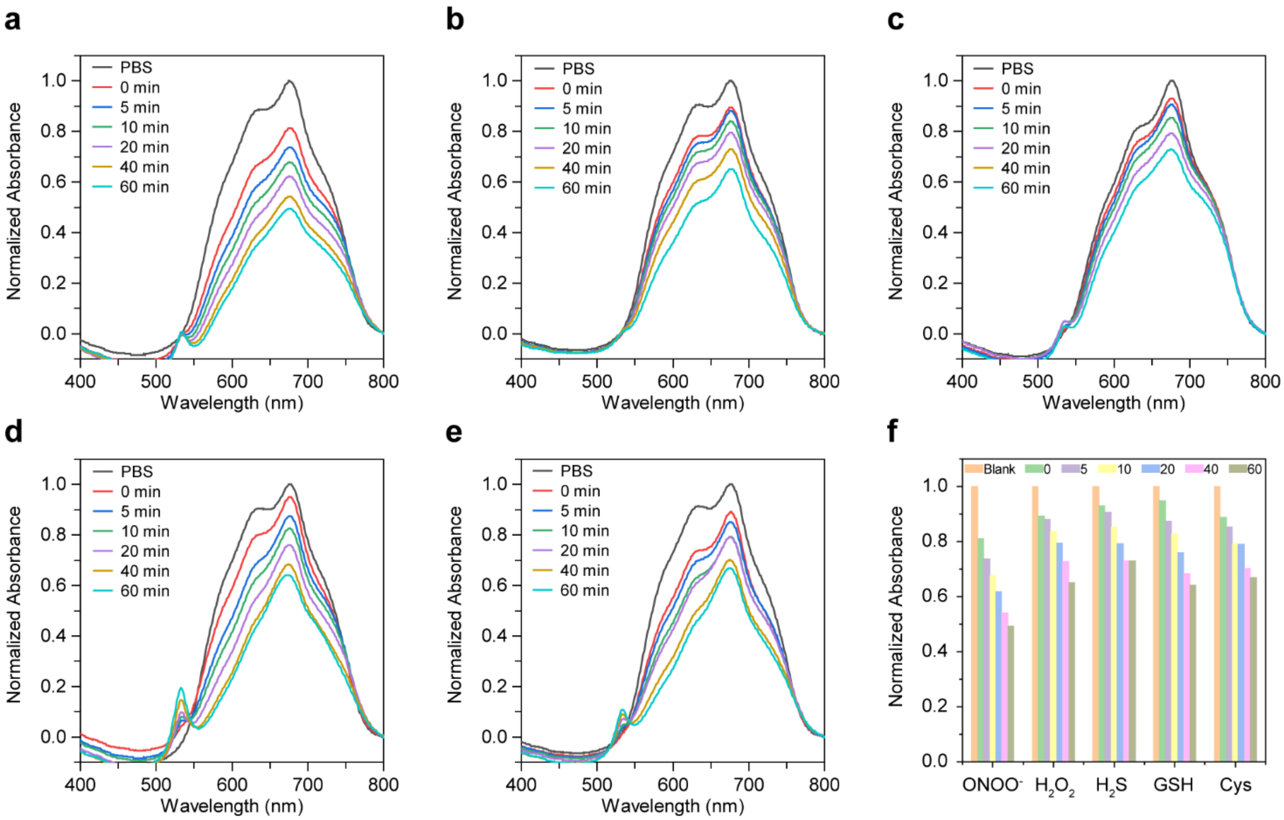

Fig. S24. Time-dependent normalized absorption spectra of Cy5.5 (10 μM) with various active agents in PBS (10 μM, pH = 7.4). a, $ONOO^-$ (5 μM); **b**, H_2O_2 (100 μM); **c**, H_2S (100 μM); **d**, GSH (1 mM); **e**, Cys (100 μM). A declining absorption profile observed in (**a-d**), indicates that Cy5.5 is susceptible to damage by reactive agents such as $ONOO^-$, H_2O_2, H_2S, GSH.

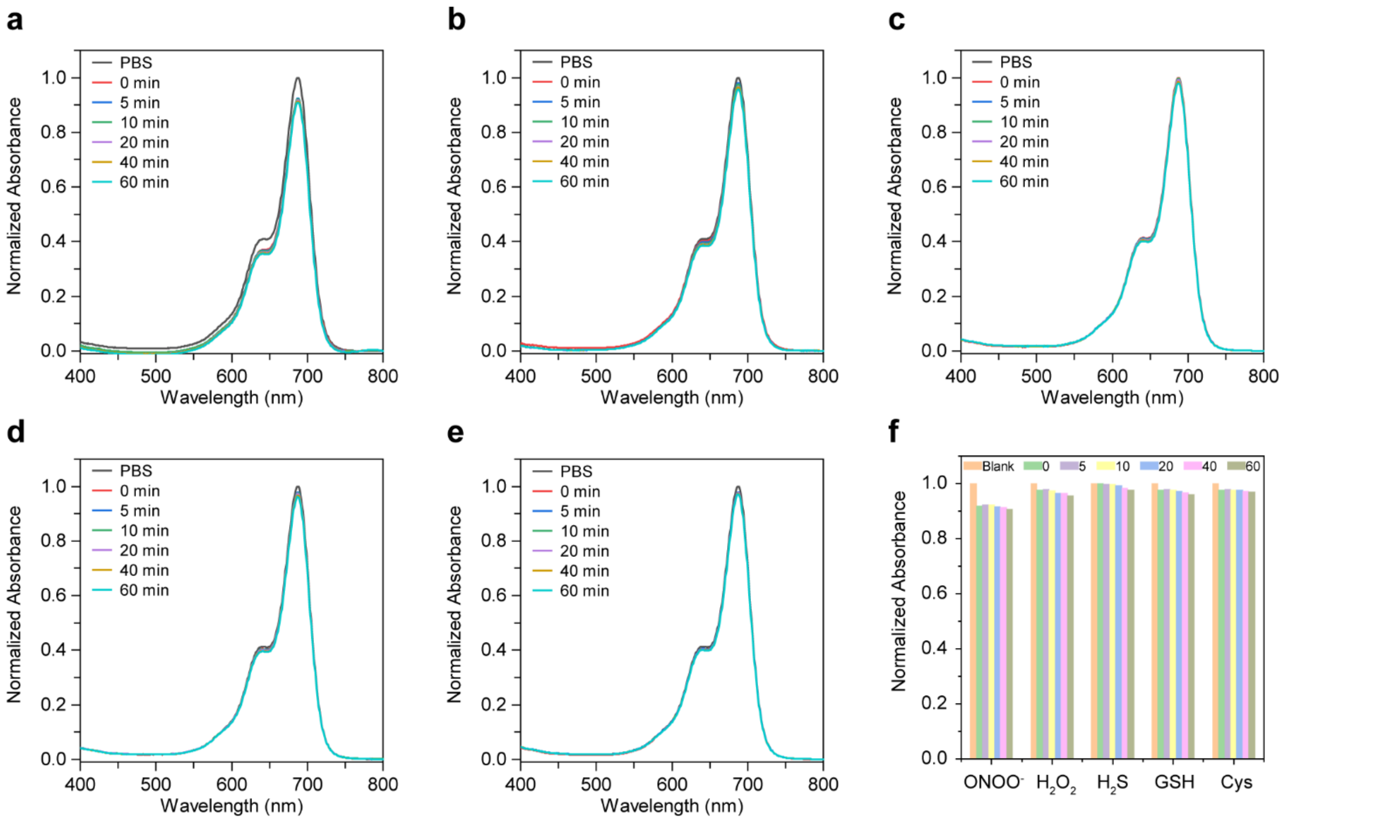

Fig. S25. Time-dependent normalized absorption spectra of Cy5.5AA@CDP (10 μM) with various active agents in PBS (10 mM, pH = 7.4). **a**, $ONOO^-$ (5 μM); **b**, H_2O_2 (100 μM); **c**, H_2S (100 μM); **d**, GSH (1 mM); **e**, Cys (100 μM). A consistent absorption profile with no peak shifts suggested minimal reaction between the supramolecular probe and the reactive agents.

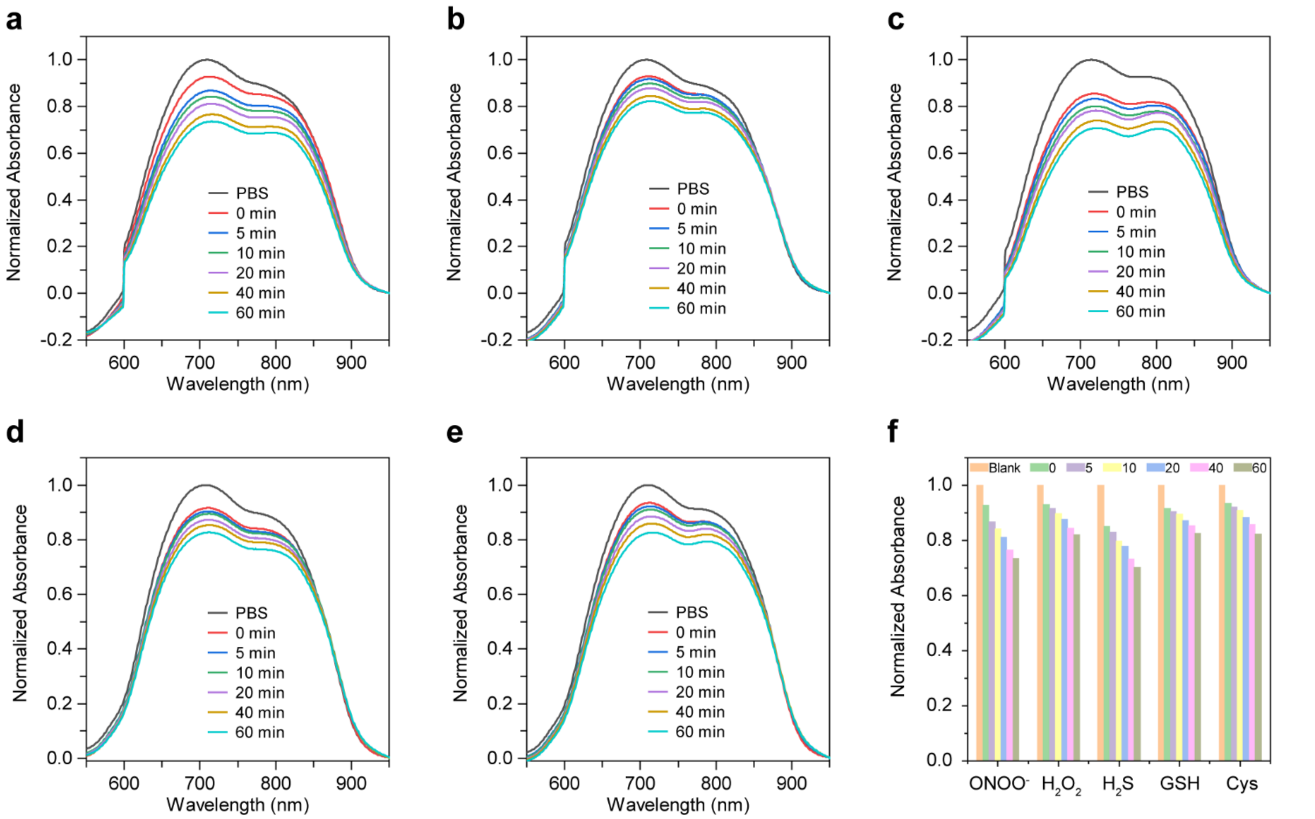

Fig. S26. Time-dependent normalized absorption spectra of Cy7.5 (10 μM) with various active agents in PBS (10 mM, pH = 7.4). a, $ONOO^-$ (5 μM); **b**, H_2O_2 (100 μM); **c**, H_2S (100 μM); **d**, GSH (1 mM); **e**, Cys (100 μM). A declining absorption profile observed in (**a-d**), indicates that Cy7.5 is susceptible to damage by reactive agents such as $ONOO^-$, H_2O_2, H_2S, GSH.

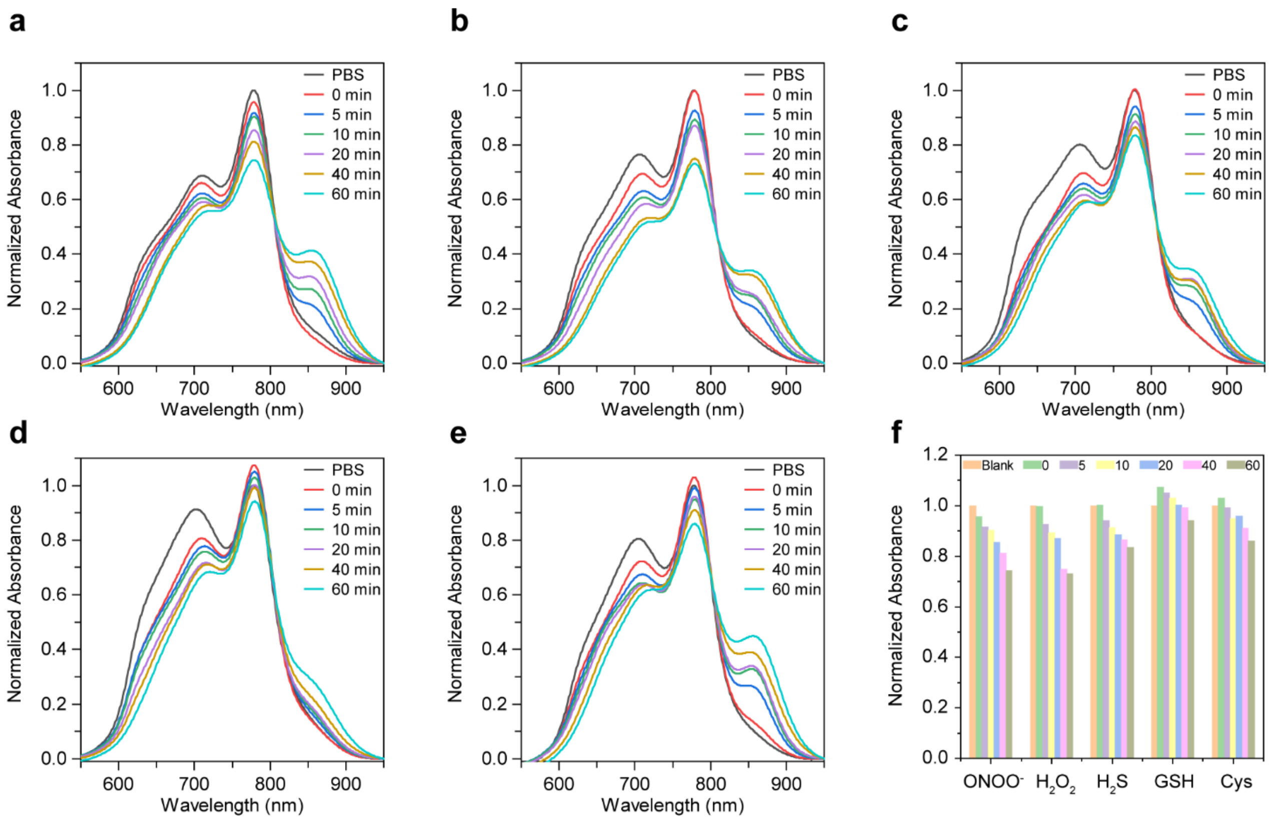

Fig. S27. Time-dependent normalized absorption spectra of ICG (10 μM) with various active agents in PBS (10 mM, pH = 7.4). **a**, $ONOO^-$ (5 μM); **b**, H_2O_2 (100 μM); **c**, H_2S (100 μM); **d**, GSH (1 mM); **e**, Cys (100 μM). A declining absorption profile observed in (**a**-**d**), indicates that ICG is susceptible to damage by reactive agents such as $ONOO^-$, H_2O_2, H_2S, GSH.

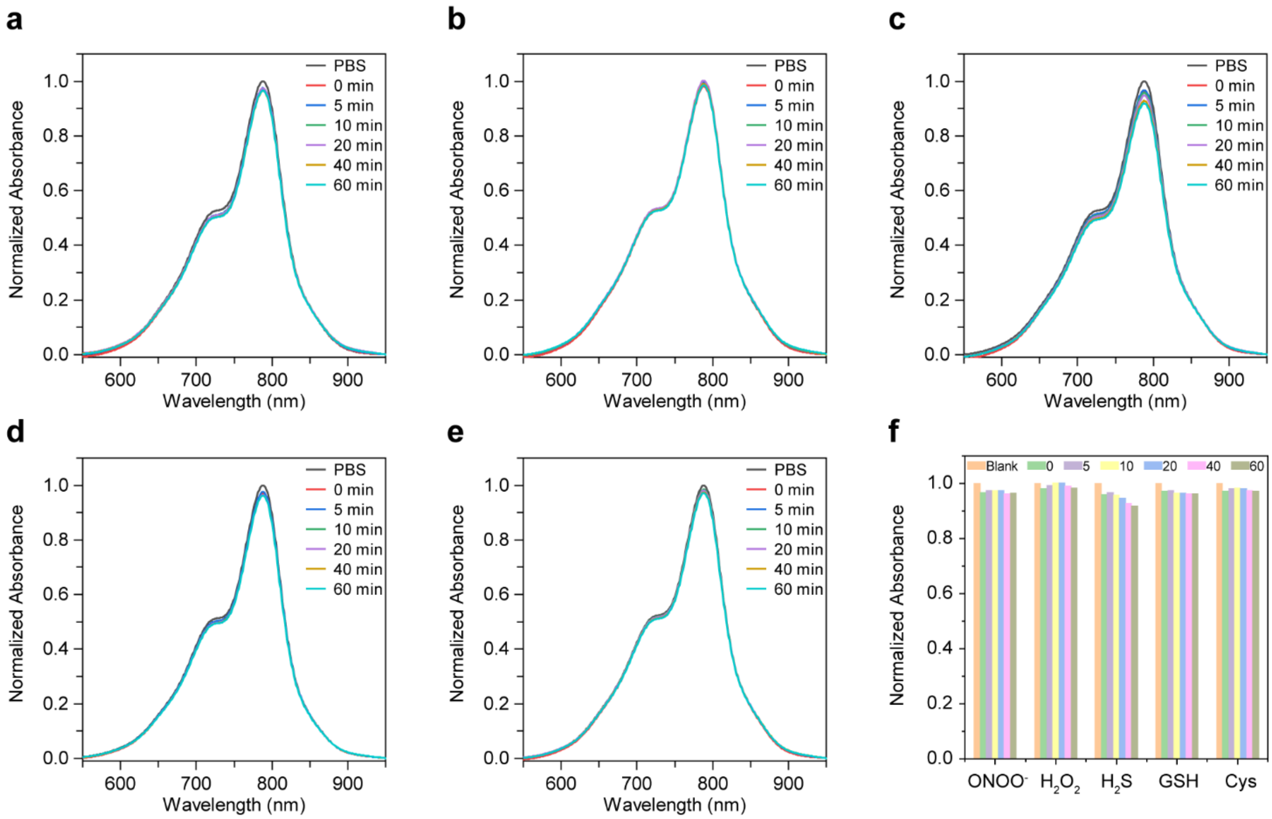

Fig. S28. Time-dependent normalized absorption spectra of Cy7.5AA@CDP (10 μM) with various active agents in PBS (10 mM, pH = 7.4). a, $ONOO^-$ (5 μM); **b**, H_2O_2 (100 μM); **c**, H_2S (100 μM); **d**, GSH (1 mM); **e**, Cys (100 μM). A consistent absorption profile with no peak shifts suggested minimal reaction between the supramolecular probe and the reactive agents.

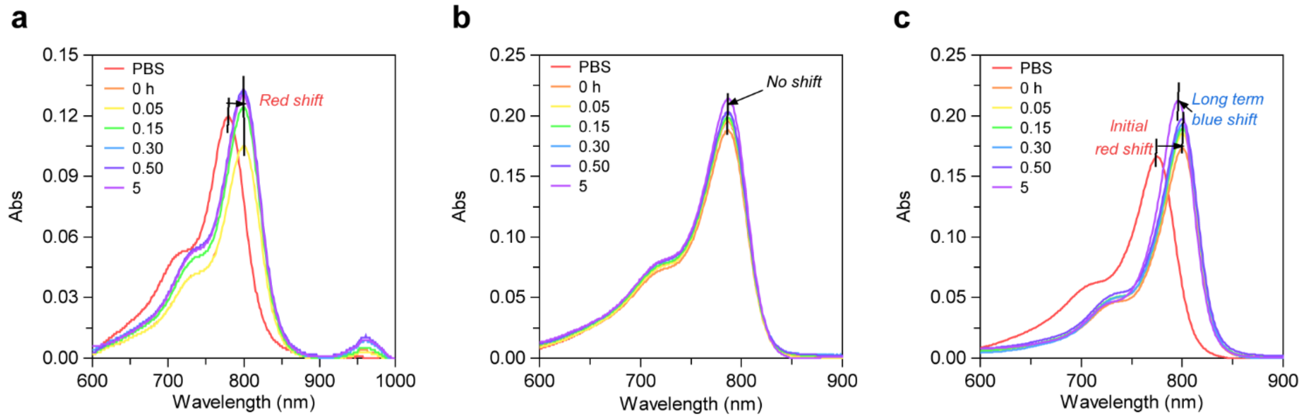

Fig. S29. Evaluation of the supramolecular assembly's influence on interactions with protein. a-c, ICG (1 μM) (**a**), Cl-Cy7AA@CDP (1 μM) (**b**), and Cl-Cy7COOH (1 μM) (**c**) were incubated with human serum albumin (HSA, 50 μM) in PBS at 37°C for 0-5 h and then recorded for UV absorption spectra. An increasing absorption profile with noticeable peak shifts in the case of ICG and Cl-Cy7COOH suggests a strong interaction with HSA (*69, 70*). In contrast, a stable absorption profile without peak shifts in the case of Cl-Cy7AA@CDP indicates minimal interaction with HSA. The use of ICG, Cl-Cy7AA@CDP and Cl-Cy7COOH is to illustrate the advantage of the multivalent assembly approach in reducing non-specific interactions with proteins, which is consistent with the findings for Cy7.5AA@CDP shown in Fig. 2H.

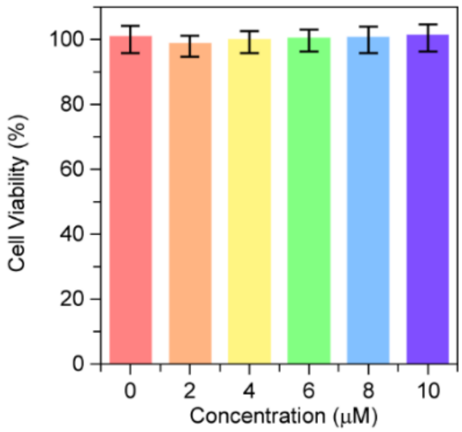

Fig. S30. Cytotoxicity of Cy7.5AA@CDP against CT-26 cells. Cells were incubated with the supramolecular probe at corresponding concentrations for 24 h. Cell viability was measured by MTT assay (*71*) and the results were reported as percentage relative to untreated cells. The data are the mean ± s.d. $n = 3$ independent experiments.

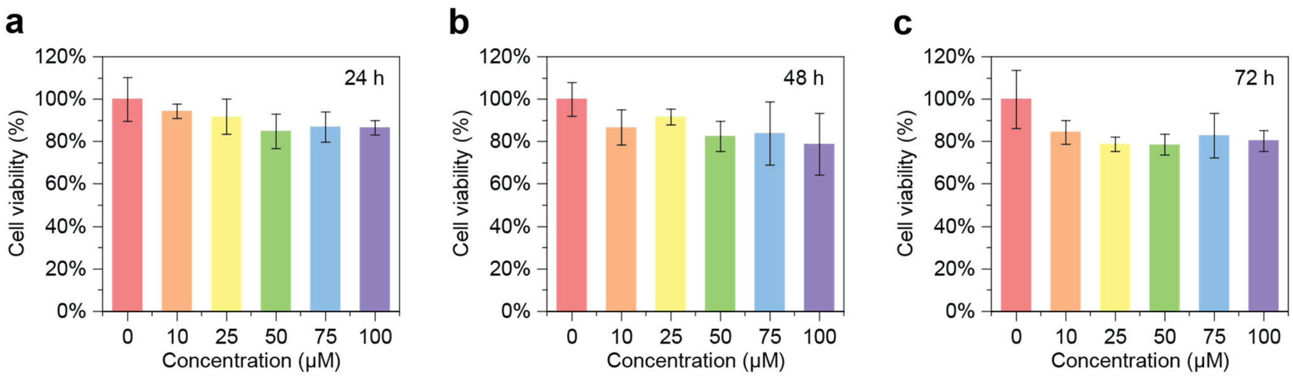

Fig. S31. Cytotoxicity of Cy7.5AA@CDP against HK-2 cells. Cells were incubated with the supramolecular probe at corresponding concentrations for 24 (**a**), 48 (**b**), and 72 (**c**) huors. Cell viability was measured by MTT assay and the results were reported as percentage relative to untreated cells. The data are the mean ± s.d. n = 3 independent experiments.

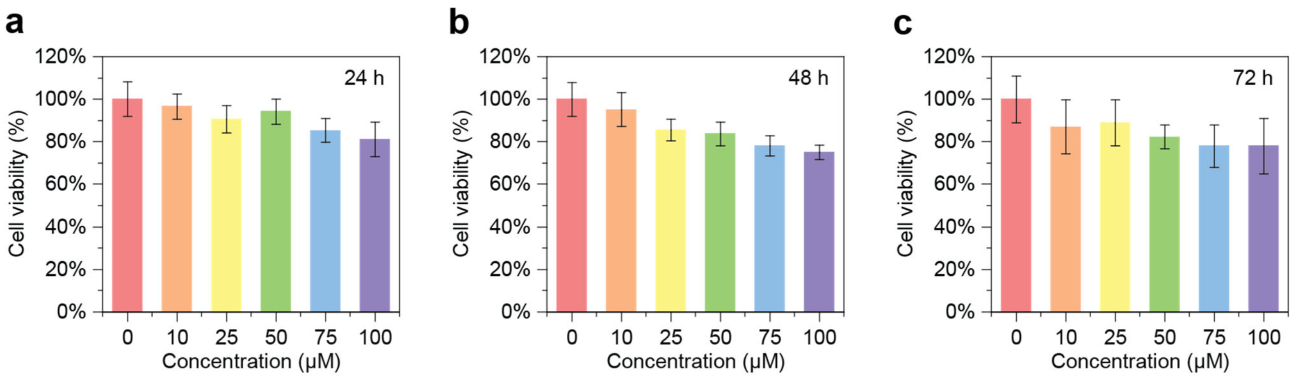

Fig. S32. Cytotoxicity of Cy7.5AA@CDP against LX-2 cells. Cells were incubated with the supramolecular probe at corresponding concentrations for 24 (**a**), 48 (**b**), and 72 (**c**) huors. Cell viability was measured by MTT assay and the results were reported as percentage relative to untreated cells. The data are the mean ± s.d. $n = 3$ independent experiments.

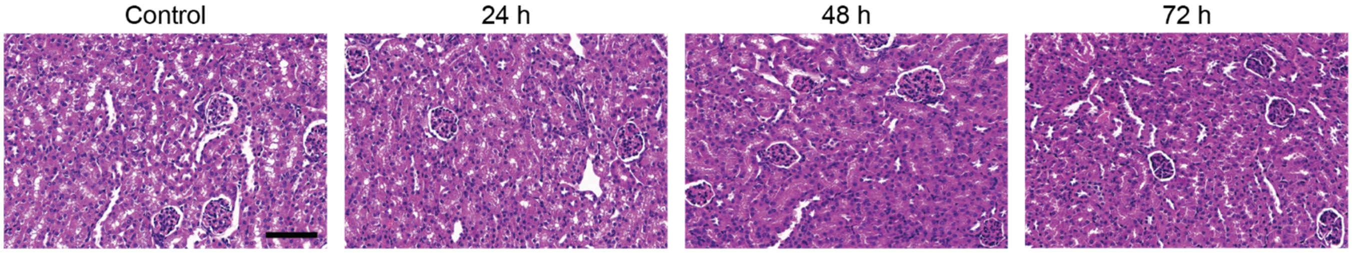

Fig. S33. Histological assessment (H&E staining) of kidney tissues of healthy mice treated with Cy7.5AA@CDP (100 μM, 100 μL injected i.v.) at 24, 48, and 72 hours post-injection. The kidney tissues exhibited normal, healthy morphology with no signs of inflammation, necrosis, or other pathological changes, indicating minimal nephrotoxicity. Scale bar: 20 μm.

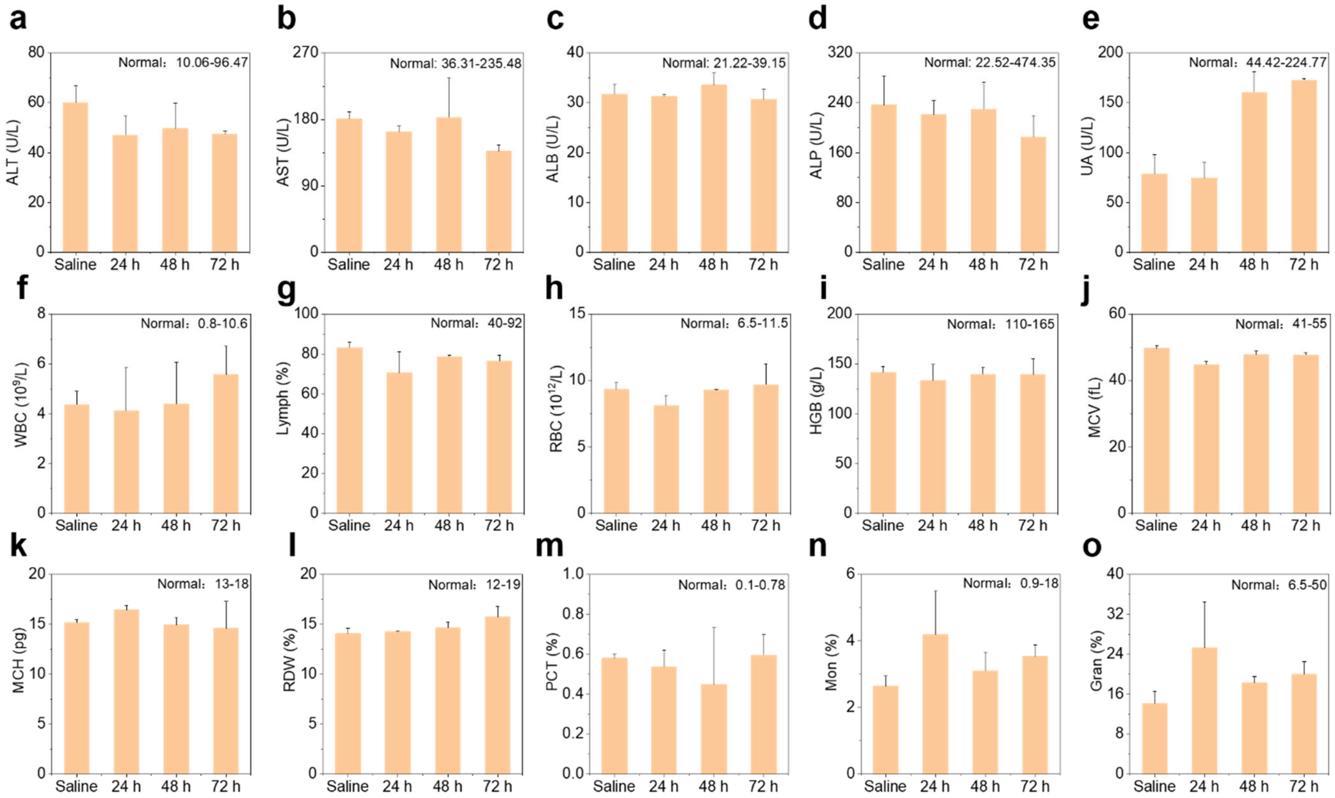

Fig. S34. Biochemistry and hematology analyses on healthy mice treated with Cy7.5AA@CDP (100 μM, 100 μL injected i.v.) at 24, 48, and 72 hours post-injection.

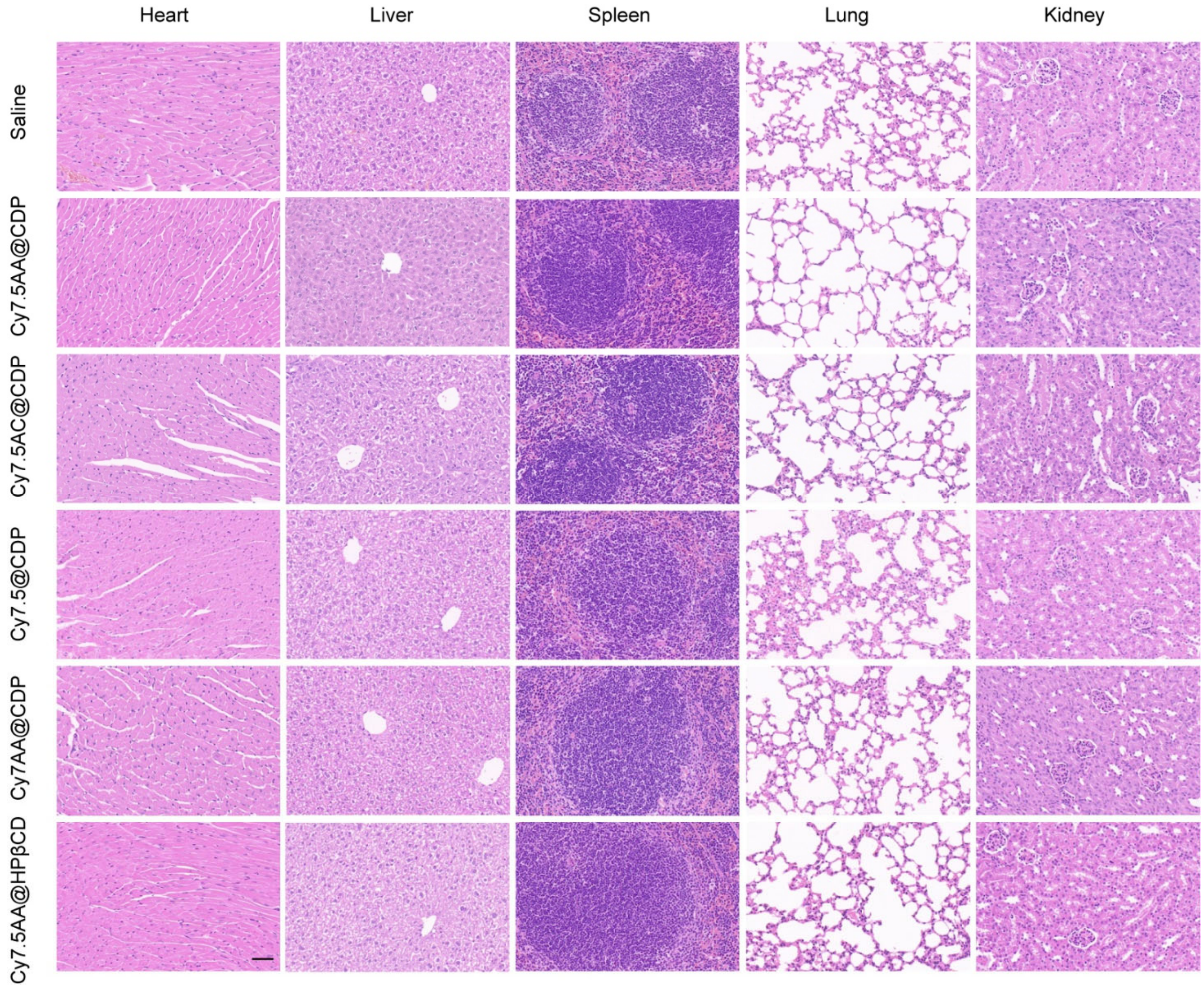

Fig. S35. Evaluation of Supramolecular Probe Biocompatibility. H&E images of five essential organs following the intravenous injection of various substances: saline, Cy7.5AA@CDP, Cy7.5AC@CDP, Cy7.5@CDP, Cy7AA@CDP, and Cy7.5AA@HPβCD. The post-injection time is 24 hours. Scale bar: 20 μm. Negligible tissue damage is observed, suggesting good biocompatibility of Cy7.5AA@CDP.

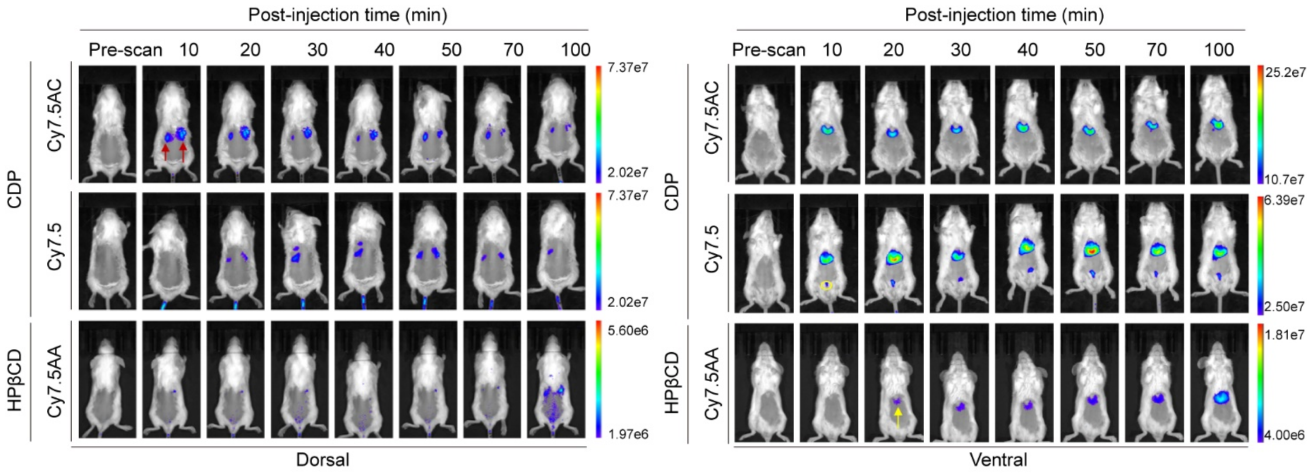

Fig. S36. Fluorescence images of live mice post-intravenous administration of various supramolecular probes at different intervals. Corresponding to the NIR fluorescence images of Cy7.5AC@CDP, Cy7.5@CDP, and Cy7.5AA@HPβCD at their respective independent scales in Fig. 2i. Red and yellow arrows indicate the kidneys and liver on the dorsal and ventral sides, respectively, while yellow circles represent the bladder on the ventral sides.

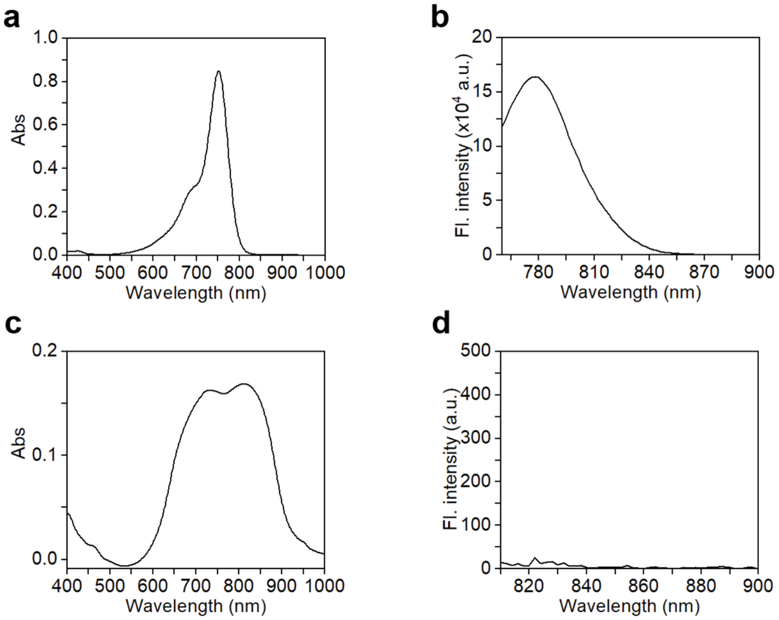

Fig. S37. Comparison of optical properties between Cy7AA@CDP and Cy7.5AA@HPβCD (10 μM, in PBS). a, Absorption spectrum of Cy7AA@CDP. **b**, Fluorescence emission spectrum of Cy7AA@CDP. c, Absorption spectrum of Cy7.5AA@ HPβCD. d, Fluorescence emission spectrum of Cy7.5AA@HPβCD.

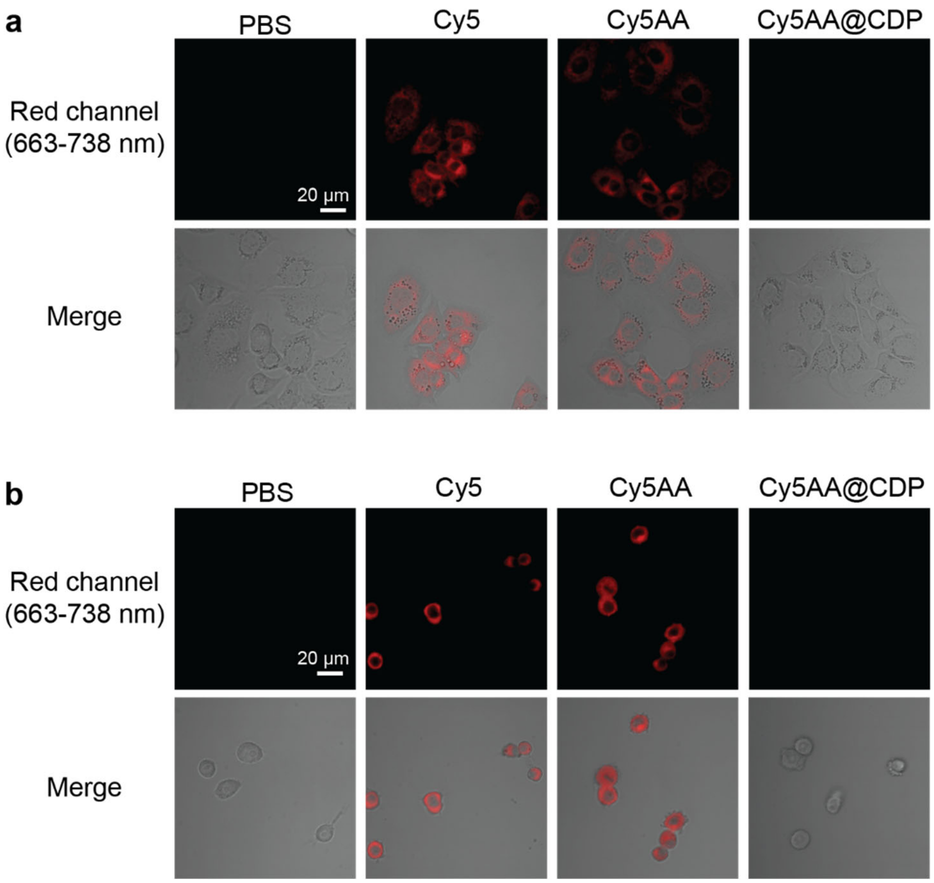

Fig. S38. Cellular uptake profiles of supramolecular probe Cy5AA@CDP. Murine hepatocyte cell line AML12 **(a)** and murine macrophage cell line RAW264.7 **(b)** were incubated with Cy5AA@CDP (10 μM) for 1 h. λ_{ex} = 640 nm, λ_{em} = 663-738 nm.

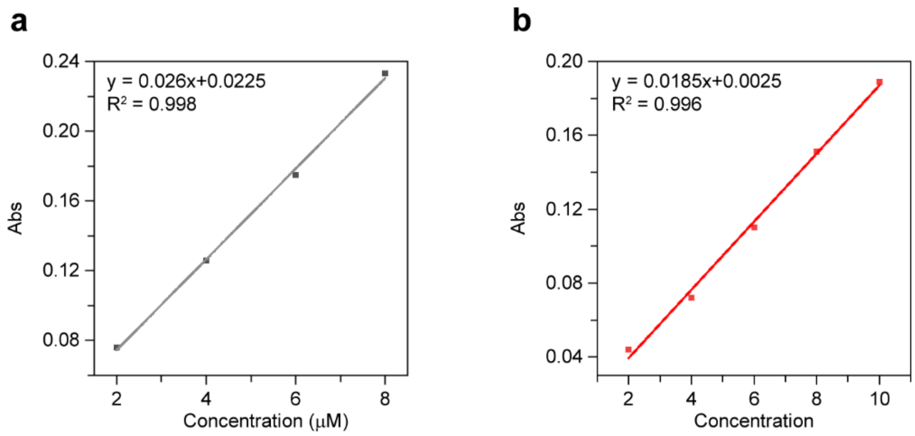

Fig. S39. Standard curves of fluorescence probes in urine for quantification of in vivo clearance profiles using absorbance. a, Standard curve of ICG. **b**, Standard curve of Cy7.5AA@CDP. The curves were established using the probes at known concentrations in urine.

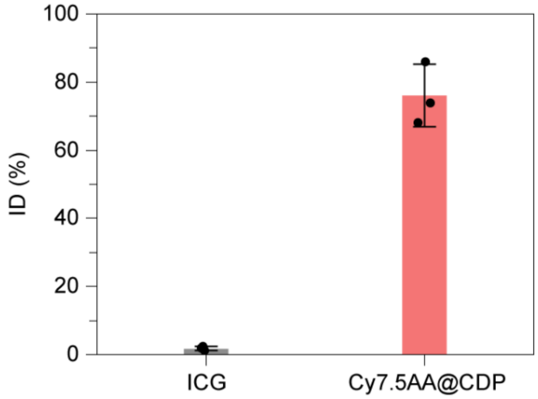

Fig. S40. Renal clearance efficiency for ICG and Cy7.5AA@CDP, 24 hours post-injection. The details for the calculations can be found in Tables S4 and S5.

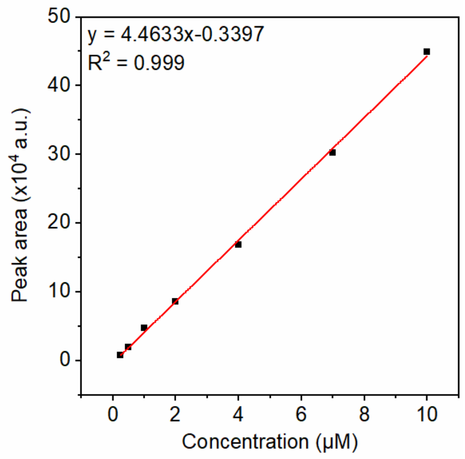

Fig. S41. Standard curve of Cy7.5AA for quantification of in vivo clearance profiles of Cy7.5AA@CDP using HPLC. The curve is established using Cy7.5AA at known concentrations in ethanol/saline solution. Absorbance wavelength is 700 nm.

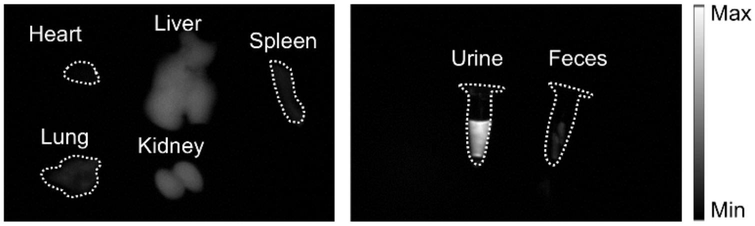

Fig. S42. NIR-II fluorescence images of the urine, feces, and major organs of mice taken 24 hours post-injection of Cy7.5AA@CDP. Strong fluorescence signals were observed in the urine, while weak signals were detected in the liver, kidney, and feces. The urine and feces samples shown were collected over the entire 24-hour period. For consistency and accurate comparison, a standardized 100 μL volume of urine was used for imaging.

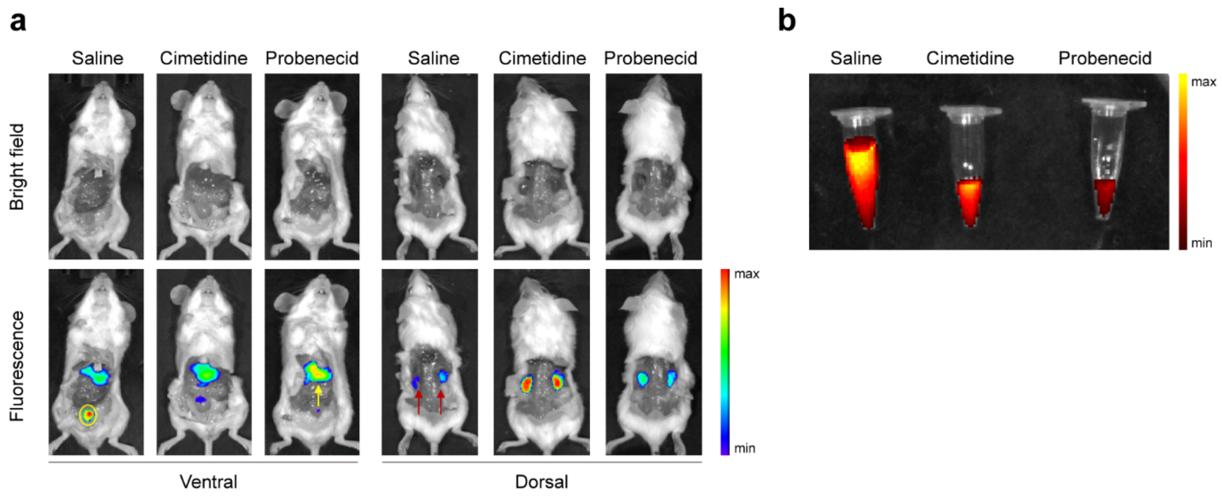

Fig. S43. Post-Administration Fluorescence Images of Mice Injected with Cy7.5AA@CDP. a, Images of mice that received intraperitoneal administrations of saline, cimetidine, and probenecid, followed 30 minutes later by an intravenous injection of Cy7.5AA@CDP. After 120 minutes, the mice were euthanized, and fluorescence images were captured separately from the dissected abdomen and dorsum. **b**, Fluorescence images of urine collected 120 minutes after the mice were injected with Cy7.5AA@CDP. Red and yellow arrows signify the kidneys and liver on the dorsal and ventral sides, respectively, while a yellow circle marks the bladder's location on the ventral side.

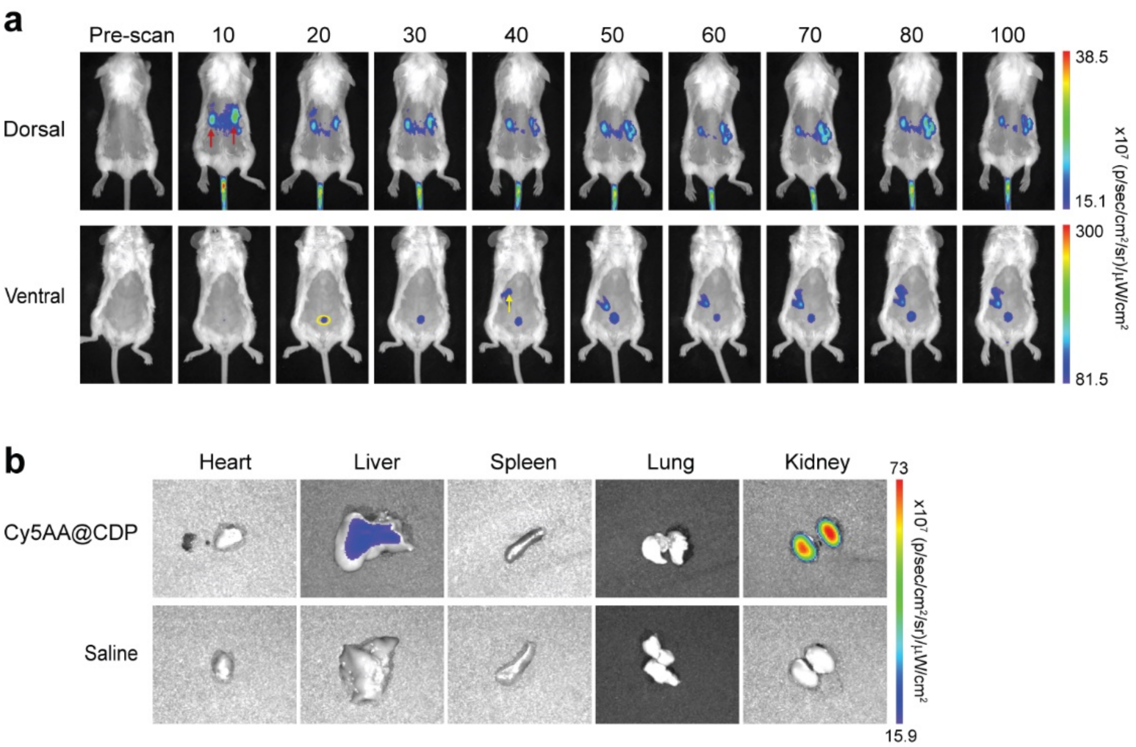

Fig. S44. Evaluation of in vivo biodistribution of Cy5AA@CDP using NIR-I fluorescence imaging. **a,** Fluorescence images of live mice at various intervals post-intravenous administration of supramolecular Cy5AA@CDP. Red and yellow arrows highlight the location of the kidneys and liver, respectively, and yellow circle highlights the location of the bladder. **b,** Representative intensity-scaled fluorescence images of mice organs 100 minutes post-injection of Cy5AA@CDP. Strong signals from the probe are observed in the kidney.

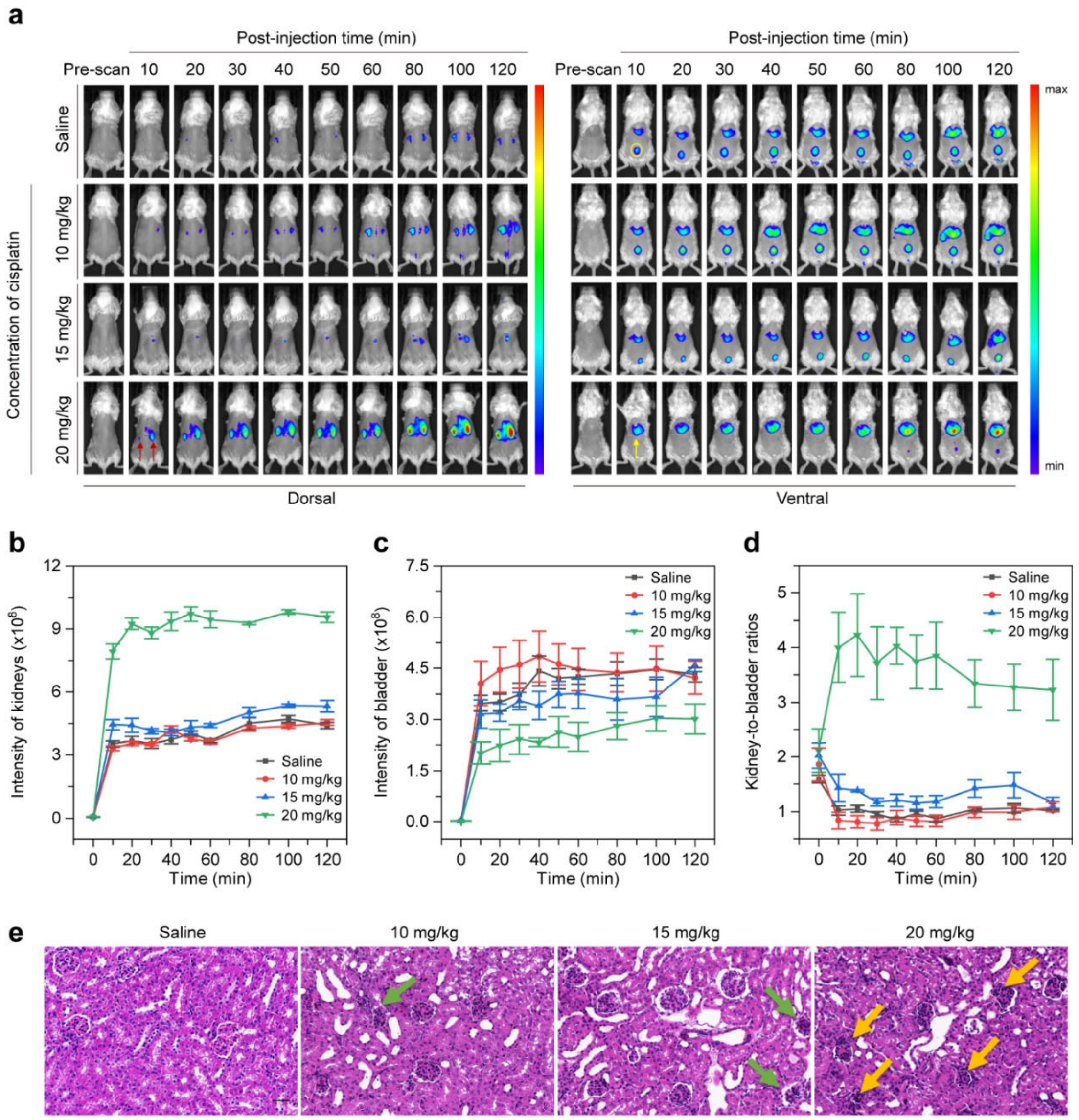

Fig. S45. Real-time in vivo fluorescence imaging of cisplatin-induced acute kidney injury (AKI). a, Representative in vivo fluorescence images of mice following intraperitoneal injections of either saline or varying doses of cisplatin (10 mg/kg, 15 mg/kg, 20 mg/kg), succeeded by intravenous injections of Cy7.5AA@CDP. **b**-**c**, Time-resolved fluorescence intensities of the kidneys (**b**) and bladder (**c**) after Cy7.5AA@CDP injection in mice that were administered saline or cisplatin. **d**, Time-dependent kidney-to-bladder fluorescence intensity ratios in mice following intraperitoneal administration of saline or cisplatin, and subsequent intravenous Cy7.5AA@CDP injection. Red and yellow arrows pinpoint the locations of the kidneys and liver on the dorsal and ventral sides, respectively, while the yellow circle marks the bladder on the ventral side. **e**, Representative photomicrographs of histological assessments (H&E staining) in paraffin-embedded kidney sections from mice post-treatment with either saline or various doses of cisplatin (10 mg/kg, 15 mg/kg, 20 mg/kg). Green and yellow arrows symbolize minor glomerular damage and necrosis, respectively. Scale bar: 20 μm.

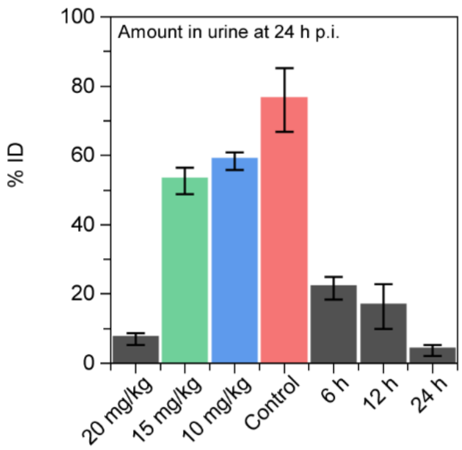

Fig. S46. Examination of 24-hour renal clearance in mice following intraperitoneal injections of either saline or various doses and durations of cisplatin treatment. Mice were administered different doses of cisplatin (10, 15, and 20 mg/kg), or a consistent dose of 20 mg/kg cisplatin for variable durations (t = 6, 12, and 24 hours). Observations reveal a consistent reduction in the renal clearance of Cy7.5AA@CDP in mice treated with cisplatin across all investigated doses and durations.

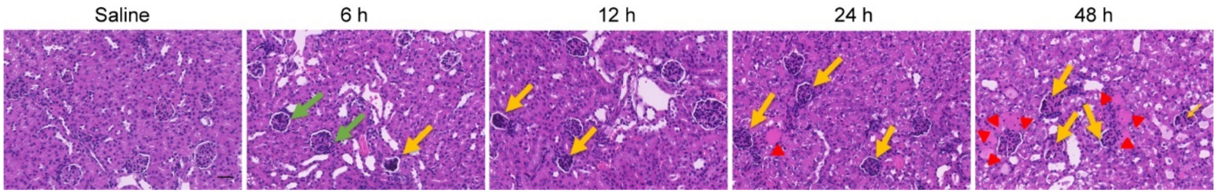

Fig. S47. Representative photomicrographs of histological assessment (H&E staining) of paraffin-embedded kidney sections from mice at varying post-treatment intervals (6, 12, 24, and 48 hours) following cisplatin administration. Green and yellow arrows denote mild glomerular damage and necrosis, respectively, while red triangles highlight the hyalinosis observed in the renal tubules. Scale bar: 20 μm.

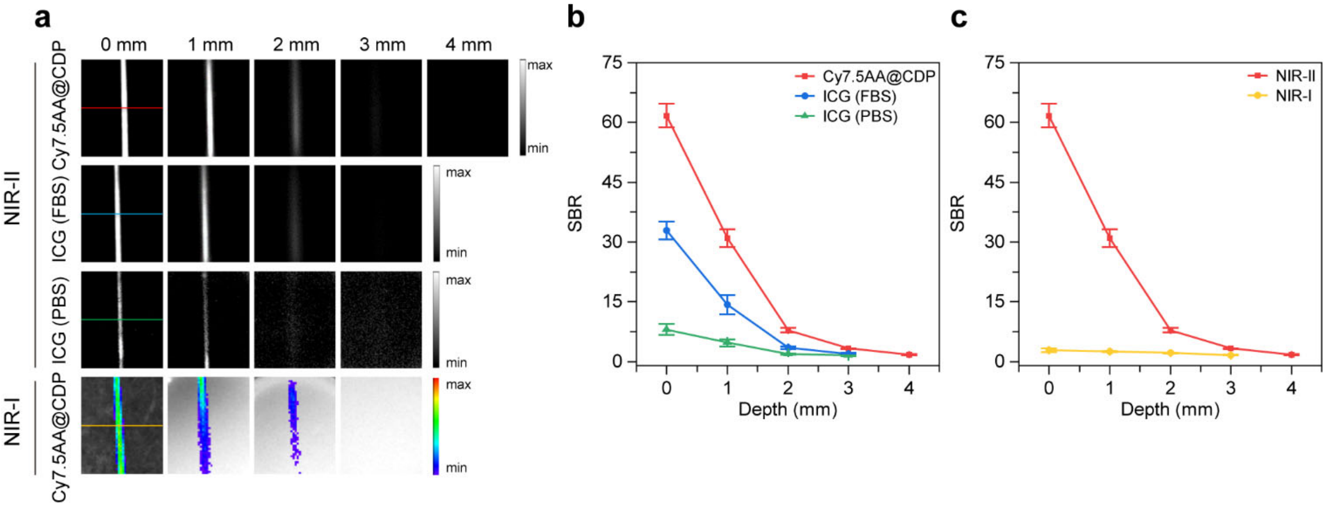

Fig. S48. In Vitro Assessment of Tissue Penetration Depth. A 1% fat emulsion was utilized to simulate biological tissue (*72, 73*). Capillaries were filled with varied solutions of ICG or Cy7.5AA@CDP (10 μM), and subsequently immersed beneath the fat emulsion solution. Increasing amounts of fat emulsion were added to mimic increasing tissue depths. Signals were collected in 1040-1700 nm. **a**, Fluorescence images of capillaries infused with Cy7.5AA@CDP (10 μM, in PBS) and ICG (10 μM, in PBS or FBS), submerged in 1% intralipid at varied depths. Signals within the 1040-1700 nm range were collected and analyzed. **b**-**c**, Calculated signal-to-background ratio (SBR) from the cross-sectional profiles of the capillary images, correlating them with the tissue depths illustrated in Fig. 48a.

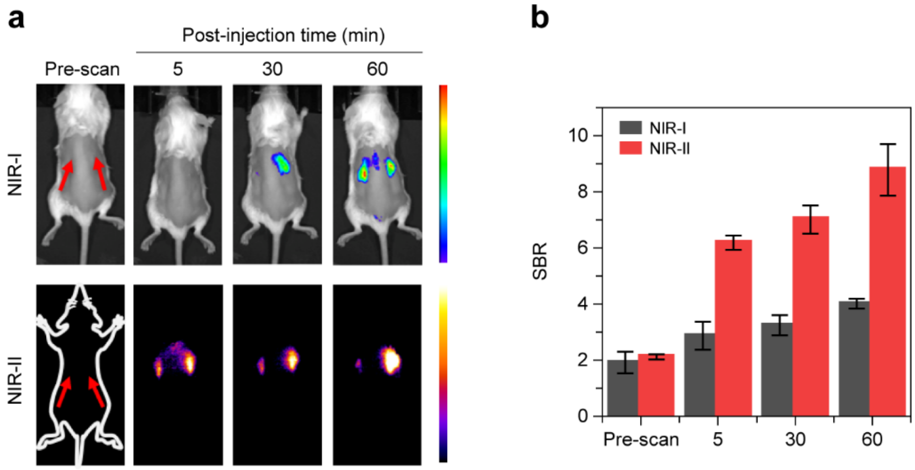

Fig. S49. In vivo tissue penetration depth study. **a**, Time-dependent NIR-I and NIR-II fluorescence imaging of mice post-intravenous administration of Cy7.5AA@CDP. **b**, Analysis of signal-to-background ratios (SBR) for Cy7.5AA@CDP during fluorescence imaging in mice.

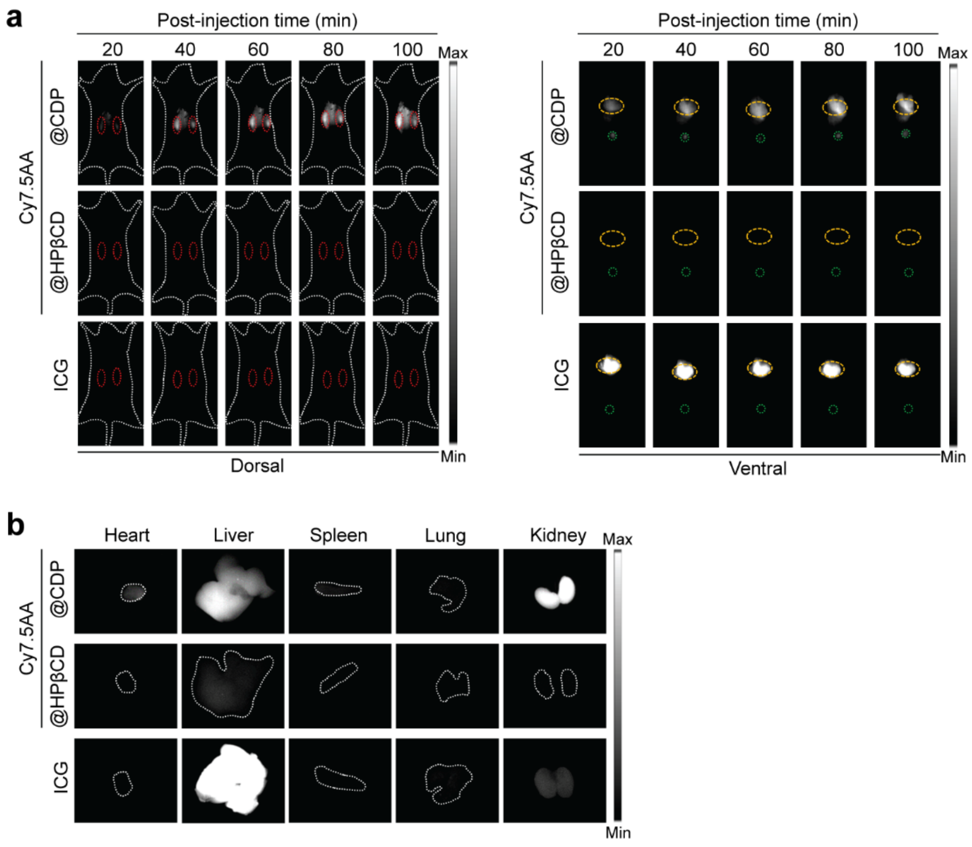

Fig. S50. Evaluation of in vivo biodistribution of the supramolecular probes using NIR-II fluorescence imaging. a, NIR-II fluorescence images of live mice at various intervals post-intravenous administration of supramolecular probes Cy7.5AA@CDP, Cy7.5AA@HPβCD, and the small molecule probe ICG (100 μM, 100 μL). Red, yellow, and green circles highlight the locations of kidney, liver, and bladder, respectively. **b,** Representative intensity-scaled NIR-II fluorescence images of mice organs 100 minutes post-injection of the probes. Strong signals from the multivalent supramolecular probe Cy7.5AA@CDP are observed in the kidney, while mice treated with ICG show strong fluorescence signals in the liver.

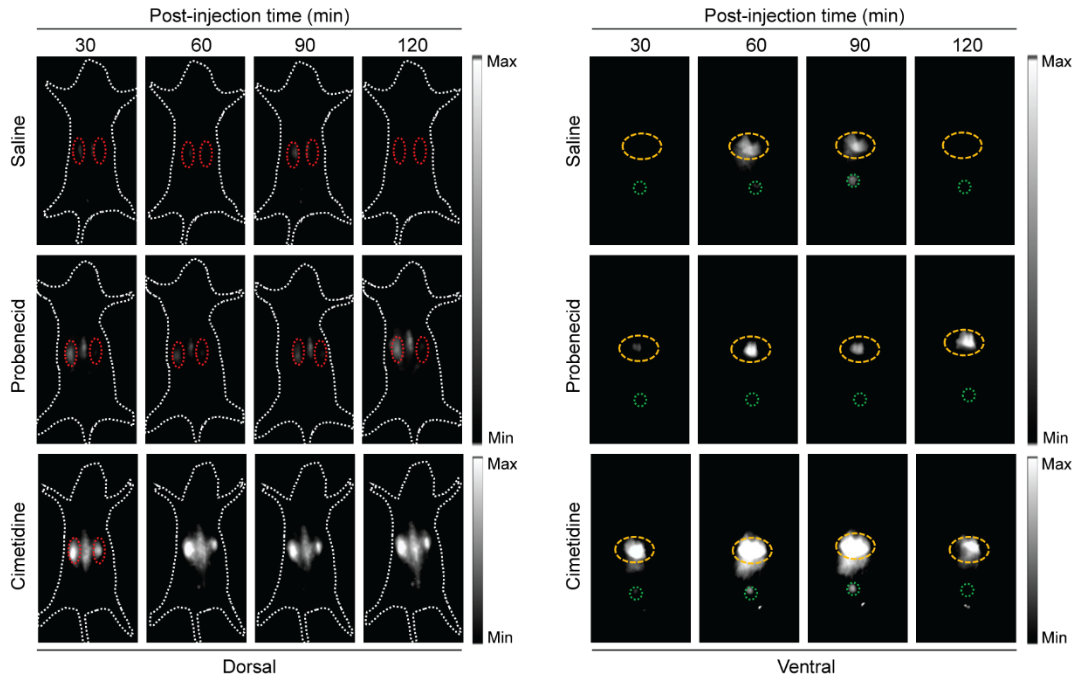

Fig. S51. NIR-II in vivo fluorescence images of live mice at diverse intervals after intraperitoneal administration of either saline, organic cation transporter cimetidine (150 mg/kg), or organic anion probenecid (150 mg/kg), followed by an intravenous injection of Cy7.5AA@CDP (100 μM, 100 μL). With the pre-treatment of renal tubular secretion and reabsorption inhibitors, the kidney fluorescence signals of mice treated with Cy7.5AA@CDP showed higher signal intensity compared to the negative controls pretreated with saline due to reduced clearance.

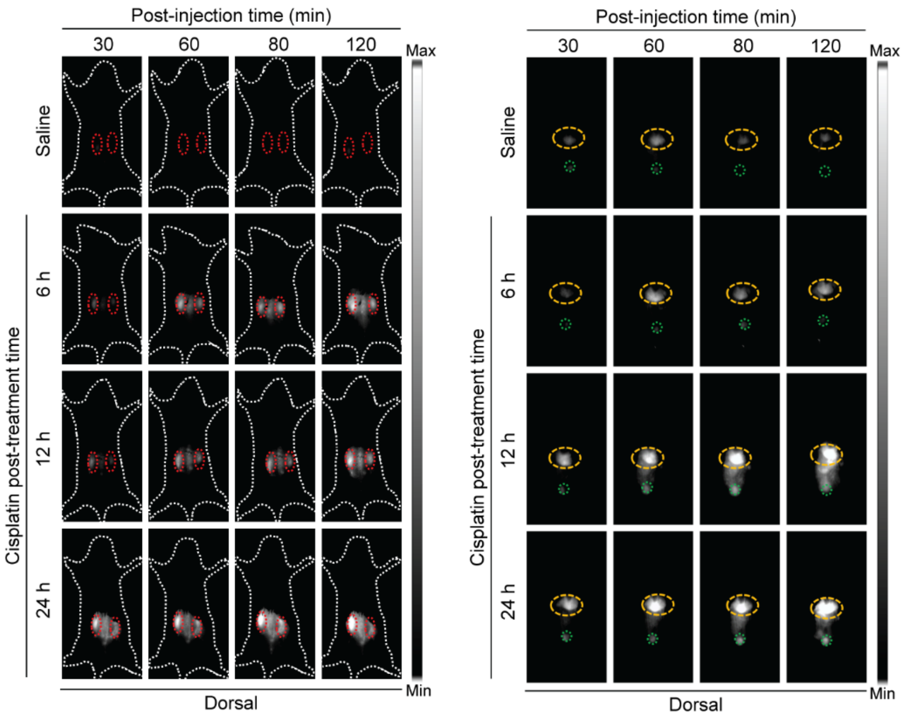

Fig. S52. NIR-II in vivo fluorescence images of mice post intraperitoneal injection with either saline or cisplatin, followed by intravenous injection of Cy7.5AA@CDP (100 μM, 75 μL). Red, yellow, and green circles highlight the locations of the kidney, liver, and bladder, respectively. For the cisplatin-treated groups, the introduction of Cy7.5AA@CDP resulted in a continuous enhancement of kidney signals, with fluorescence intensity surpassing that of the control group. This phenomenon can be attributed to reduced renal tubular secretion and reabsorption caused by cisplatin-induced AKI, resulting in the accumulation of Cy7.5AA@CDP in the kidney.

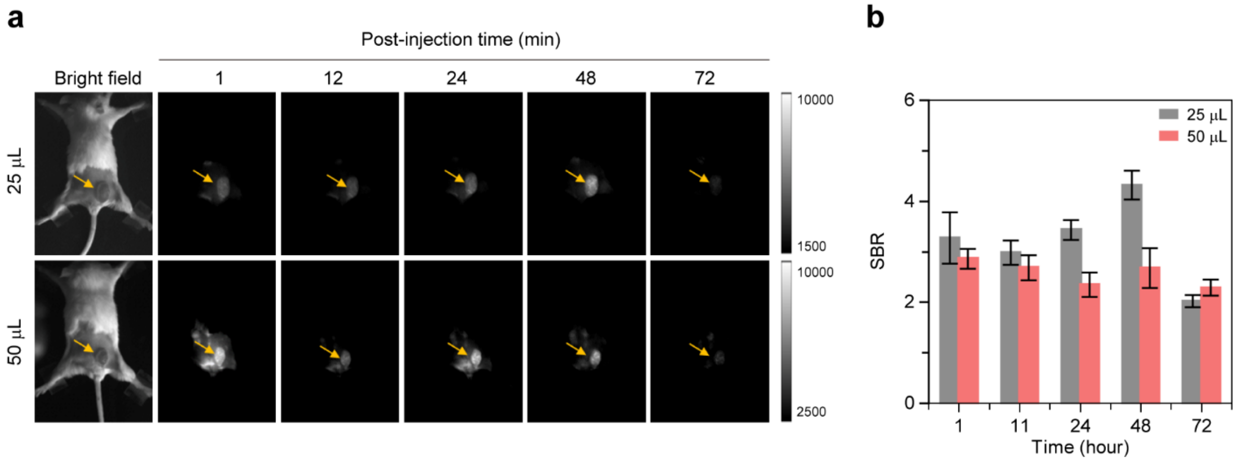

Fig. S53. NIR-II fluorescence-guided in vivo tumor imaging. a, Time-dependent NIR-II fluorescence imaging of mice bearing subcutaneous CT-26 tumor xenografts post-intravenous administration of varied doses of Cy7.5AA@CDP. **b**, Analysis of signal-to-background ratios (SBR) during NIR-II imaging of mice with subcutaneous CT-26 tumors following the administration of Cy7.5AA@CDP. Yellow arrows indicate tumor regions. The administered volumes of Cy7.5AA@CDP were 25 and 50 μL.

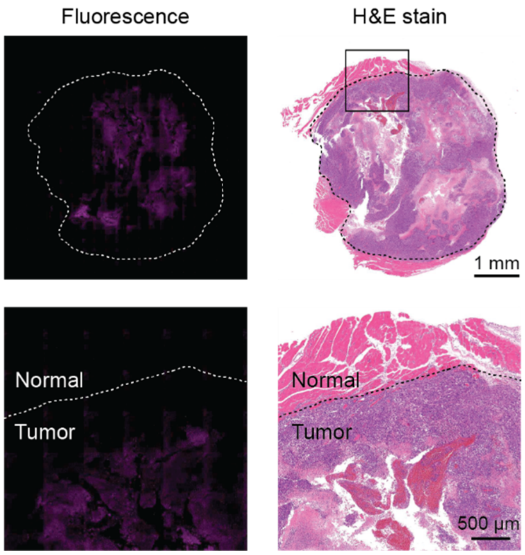

Fig. S54. Section image of CT26 tumor-normal tissue boundaries. The results demonstrate that fluorescence signals from Cy7.5AA@CDP are confined to the tumor tissues and are not present in adjacent normal tissues.

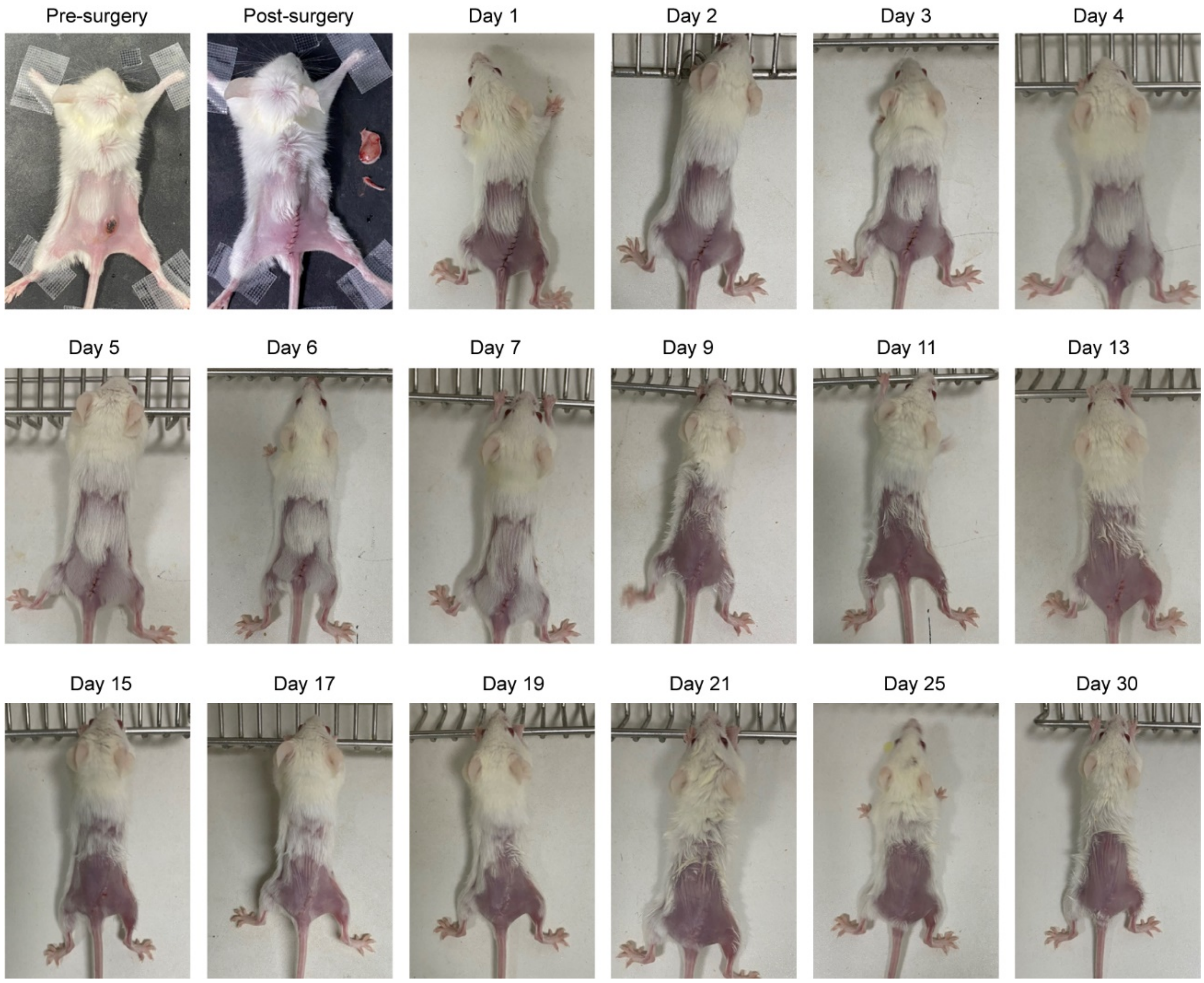

Fig. S55. Post-surgical health monitoring of mice. Following surgery, the mice exhibited good health and showed no indications of tumor recurrence.

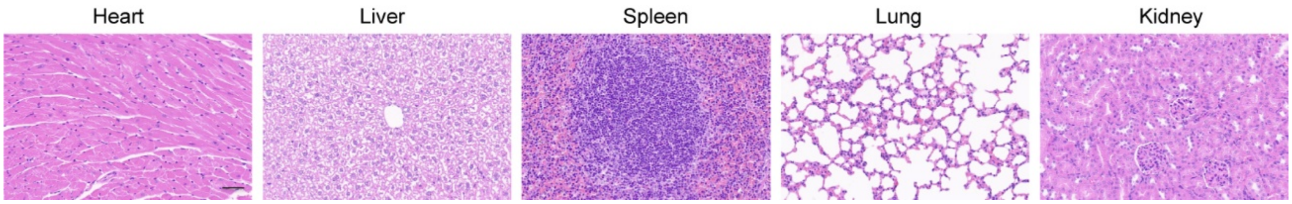

Fig. S56. Representative photomicrographs of histological assessment (H&E staining) of major organs including heart, liver, spleen, lung, and kidney from mice one month after surgery. Post-surgery, the mice displayed robust health, no signs of tumor recurrence, and prolonged postoperative survival. Scale bar: 20 μm.

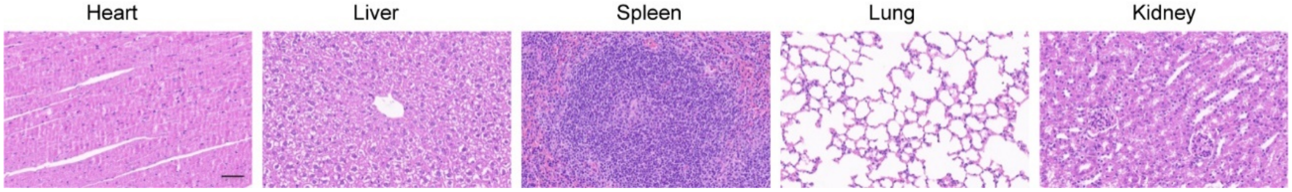

Fig. S57. Representative photomicrographs of histological assessment (H&E staining) of major organs including heart, liver, spleen, lung, and kidney from mice half a month after mastectomy. Post-surgery, the mice displayed robust health, no signs of tumor recurrence, and prolonged postoperative survival. Scale bar: 20 μm.

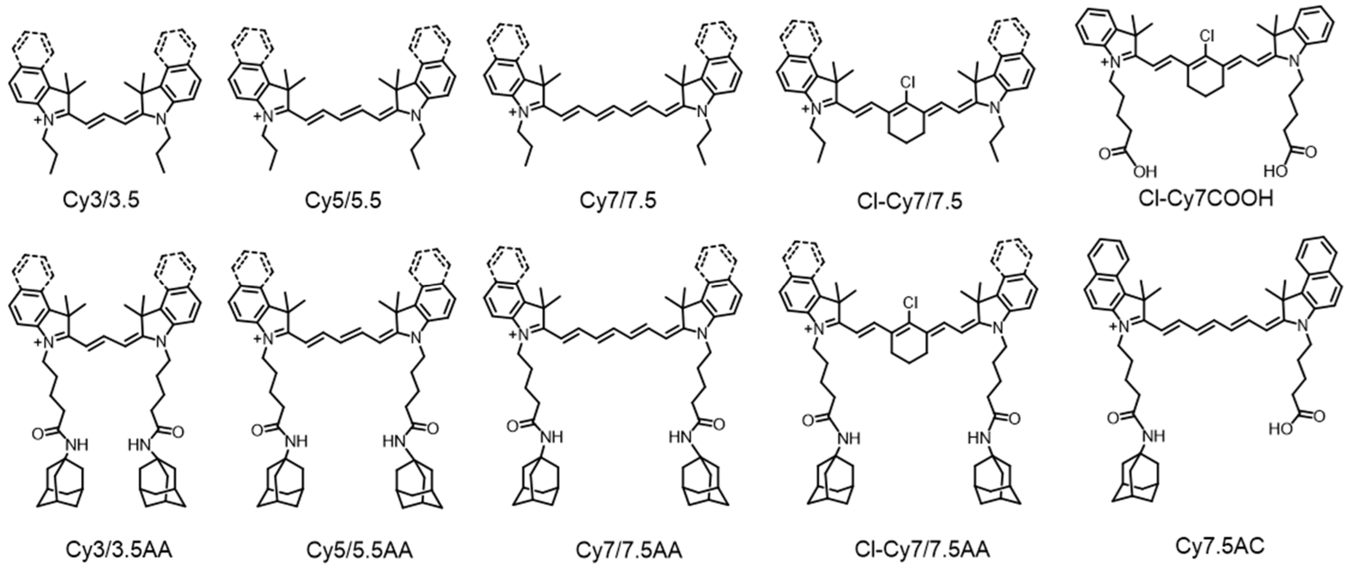

Fig. S58. Structures of the cyanine dyes. To introduce multivalent host-guest interactions, aimed at increasing complexation strength and improving the optical properties of supramolecular probes in aqueous media, indo or benzo[e]indo moieties were integrated as the terminal groups of the symmetric polymethine skeleton. This approach resulted in compounds denoted as Cyn and Cyn.5, where n = 3, 5, 7. Adamantane groups were introduced via the nitrogen atoms inherent in the dye structures, yielding symmetric derivatives CynAA and Cyn.5AA. Asymmetric Cy7.5AC bearing one adamantane was also prepared. Cl-Cy7COOH was synthesized and used to evaluate serum protein binding properties.

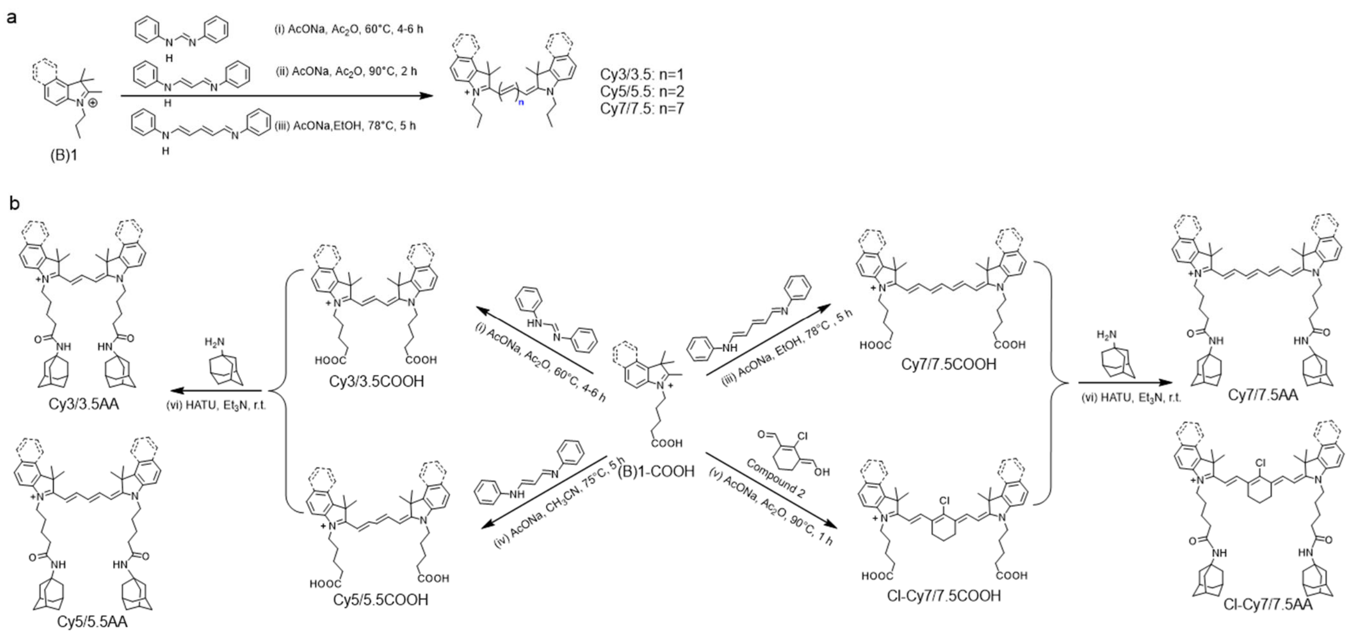

Fig. S59. Synthetic routes for cyanine dyes. a, Synthetic routes of Cy3/3.5、Cy5/5.5、Cy7/7.5 probes. These compounds were synthesized according to previous work reported (*74-78*). **b**, Synthetic routes of Cy3/3.5AA、Cy5/5.5AA、Cy7/7.5AA and Cl-Cy7/7.5AA probes. (B)1-COOH and compound 2 were synthesized according to previous methods (*79*). Reagents and conditions: (i) *N*, *N*'-Diphenylformamidine, AcONa, Ac_2O, 60℃, 4-6 h. (ii) Malonaldehyde Dianilide Hydrochloride, AcONa, Ac_2O, 90℃, 2 h. (iii) *N*-((2*E*,4*E*)-5-(Phenylamino)penta-2,4-dien-1-ylidene)aniline hydrochloride, AcONa, EtOH, 78℃, 5 h. (iv) Malonaldehyde Dianilide xHydrochloride, AcONa, CH_3CN, 75℃, 5 h. (v) Compound 2, AcONa, Ac_2O, 90℃, 1 h. (vi) Adamantan-1-amine, O-(7-Azabenzotriazol-1-yl)-*N*, *N*, *N* ', *N* '-tetramethyl uronium hexafluorophosphate (HATU), triethylamine (Et_3N), room temperature, 1-2 h.

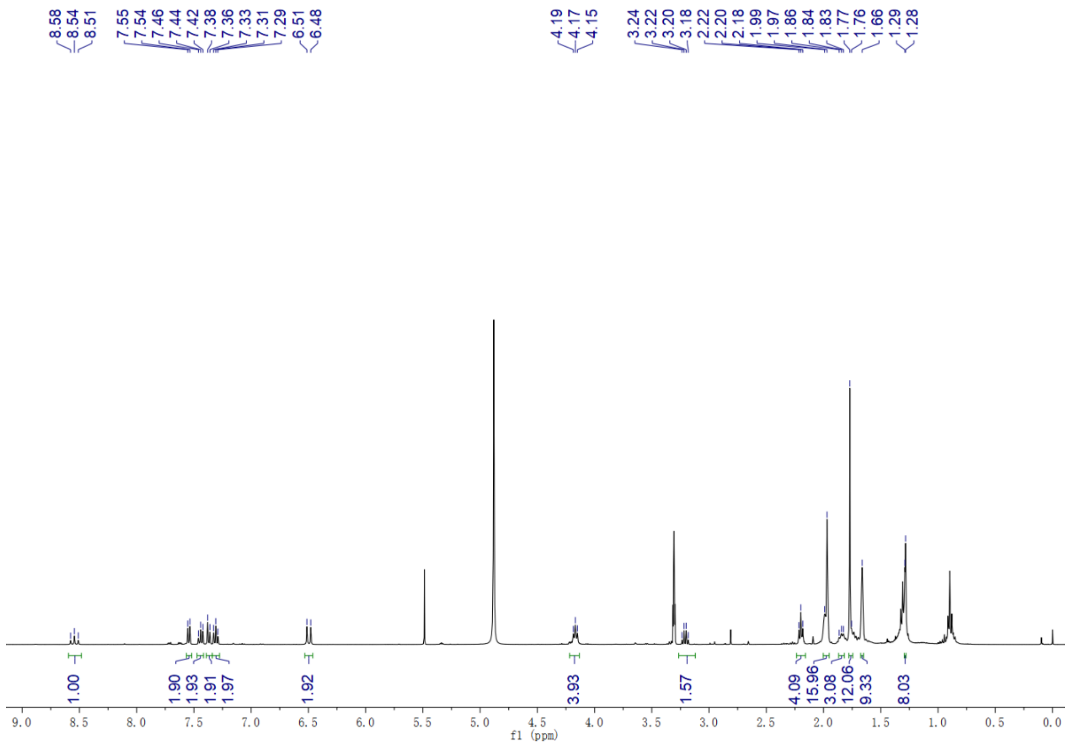

Fig. S60. ^{1}H NMR (400 MHz, Methanol-d_4) spectrum of Cy3AA.

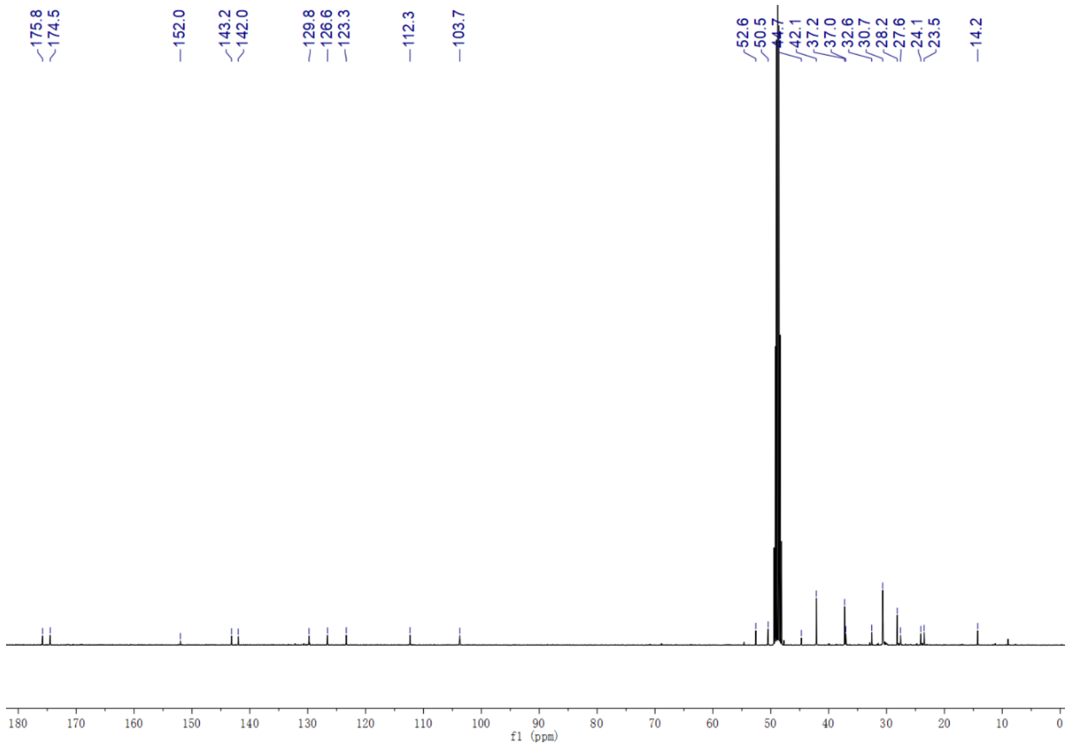

Fig. S61. ^{13}C NMR (101 MHz, Methanol-d_4) spectrum of Cy3AA.

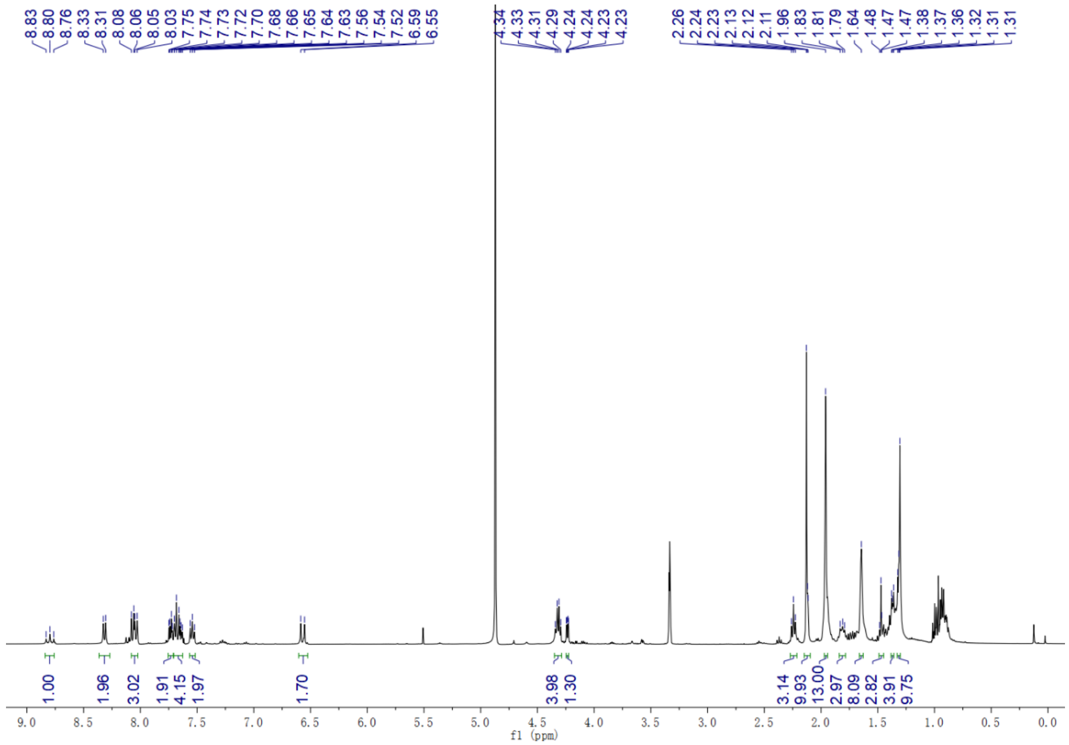

Fig. S62. ^{1}H NMR (400 MHz, Methanol-d_4) spectrum of Cy3.5AA.

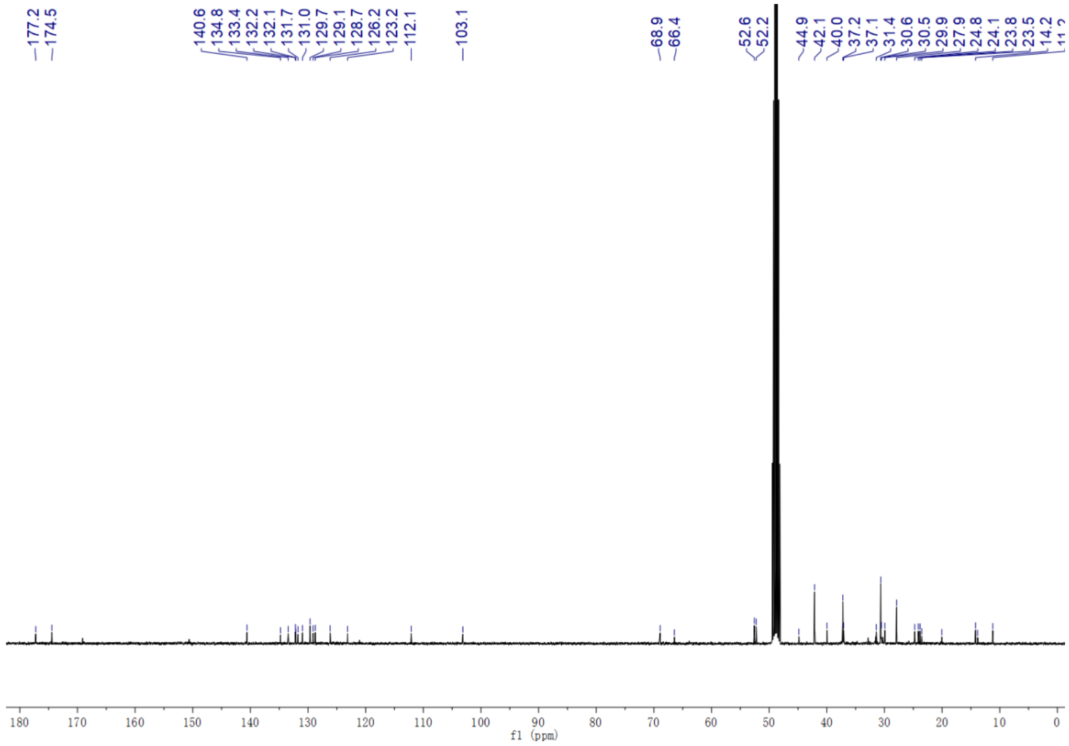

Fig. S63. ^{13}C NMR (101 MHz, Methanol-d_4) spectrum of Cy3.5AA.

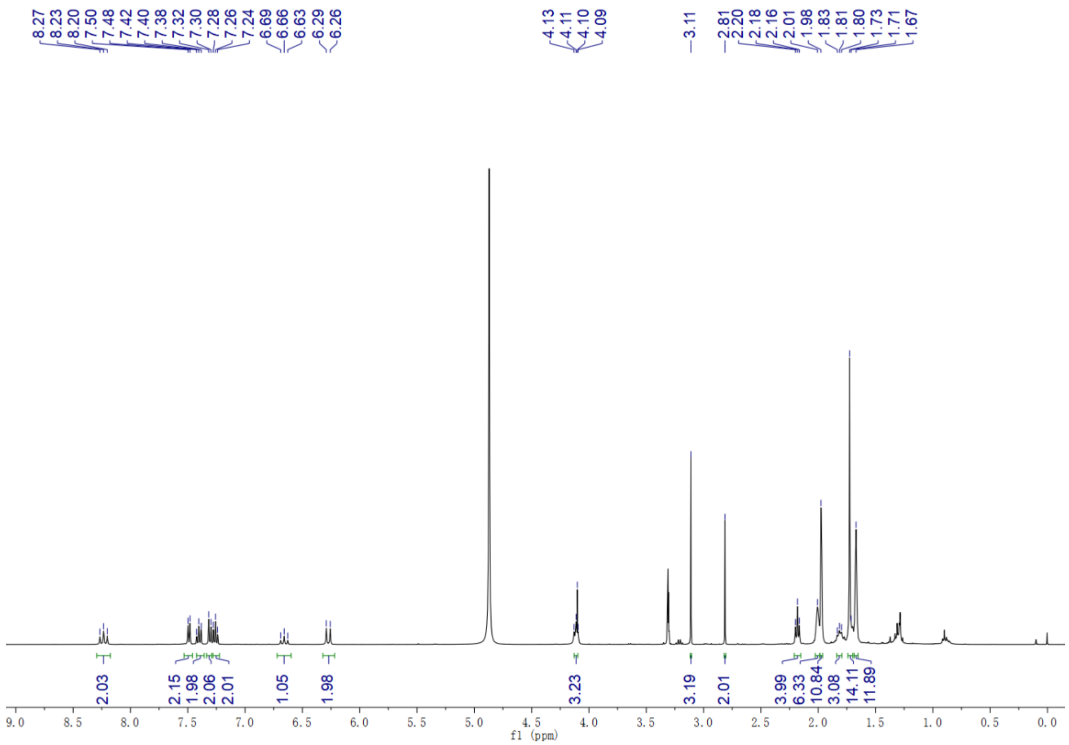

Fig. S64. ^{1}H NMR (400 MHz, Methanol-d_4) spectrum of Cy5AA.

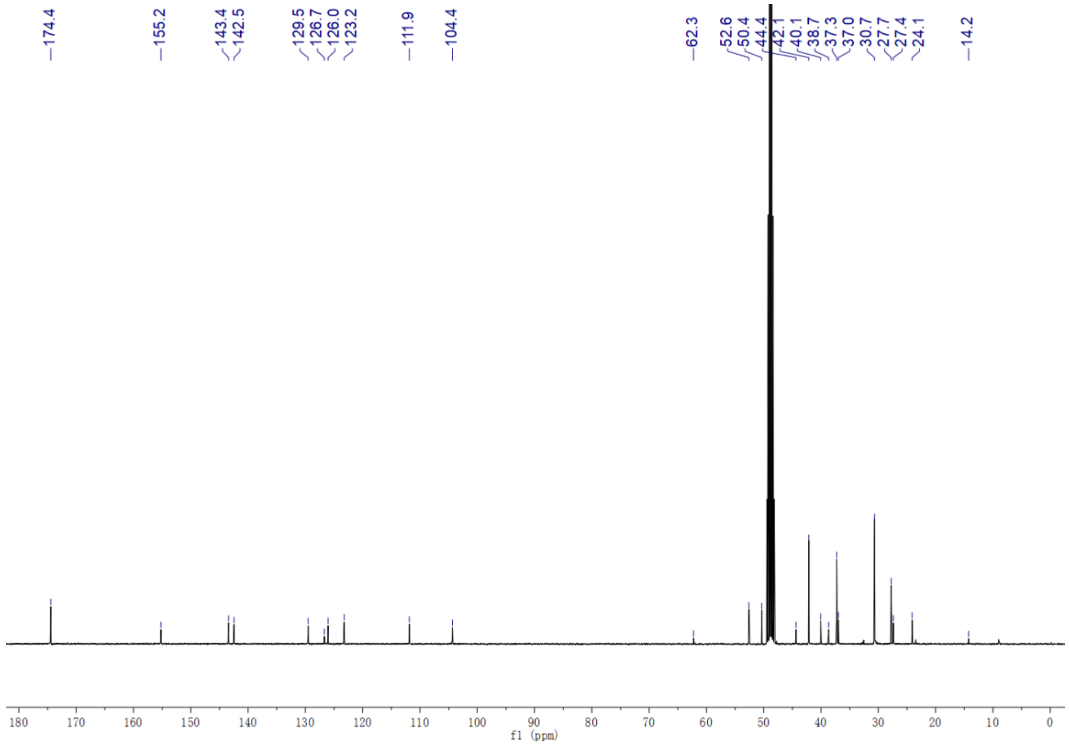

Fig. S65. ^{13}C NMR (101 MHz, Methanol-d_4) spectrum of Cy5AA.

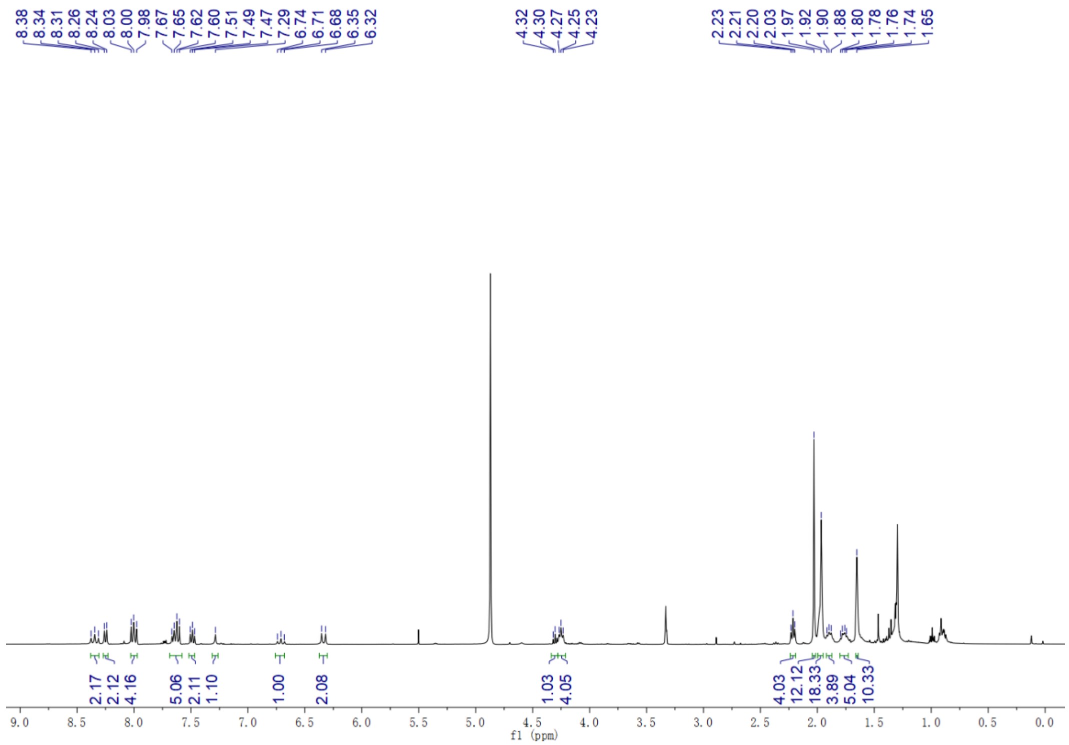

Fig. S66. ^{1}H NMR (400 MHz, Methanol-d_4) spectrum of Cy5.5AA.

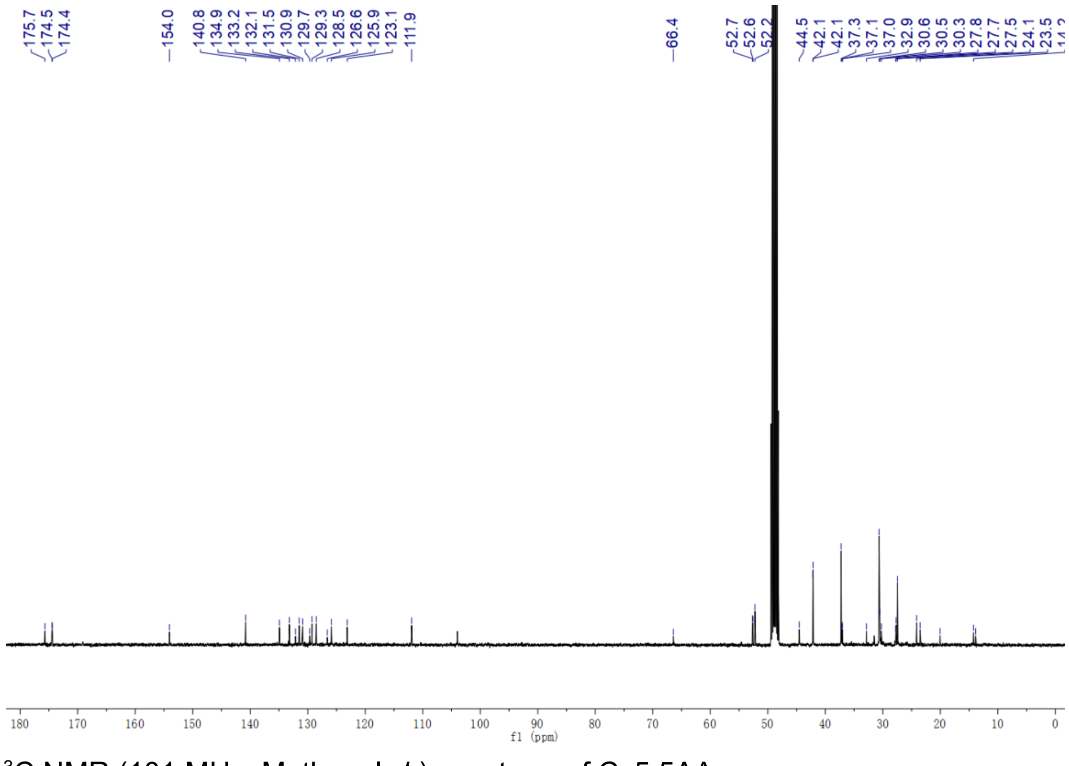

Fig. S67. ^{13}C NMR (101 MHz, Methanol-d_4) spectrum of Cy5.5AA.

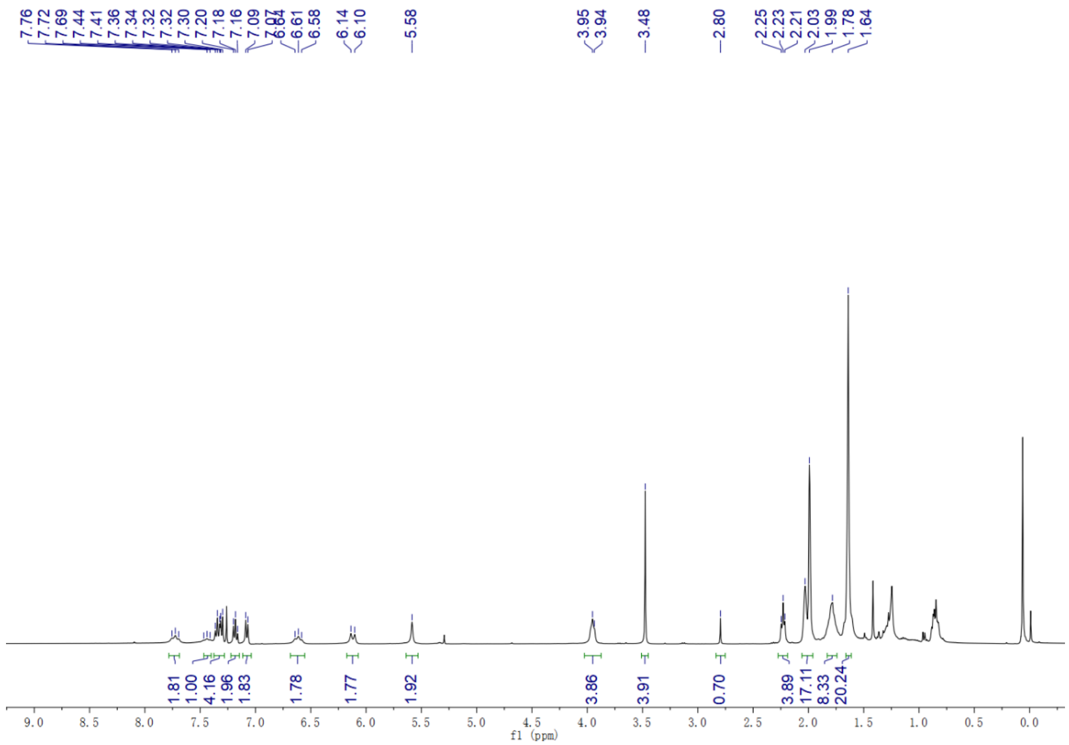

Fig. S68. ^{1}H NMR (400 MHz, Chloroform-*d*) spectrum of Cy7AA.

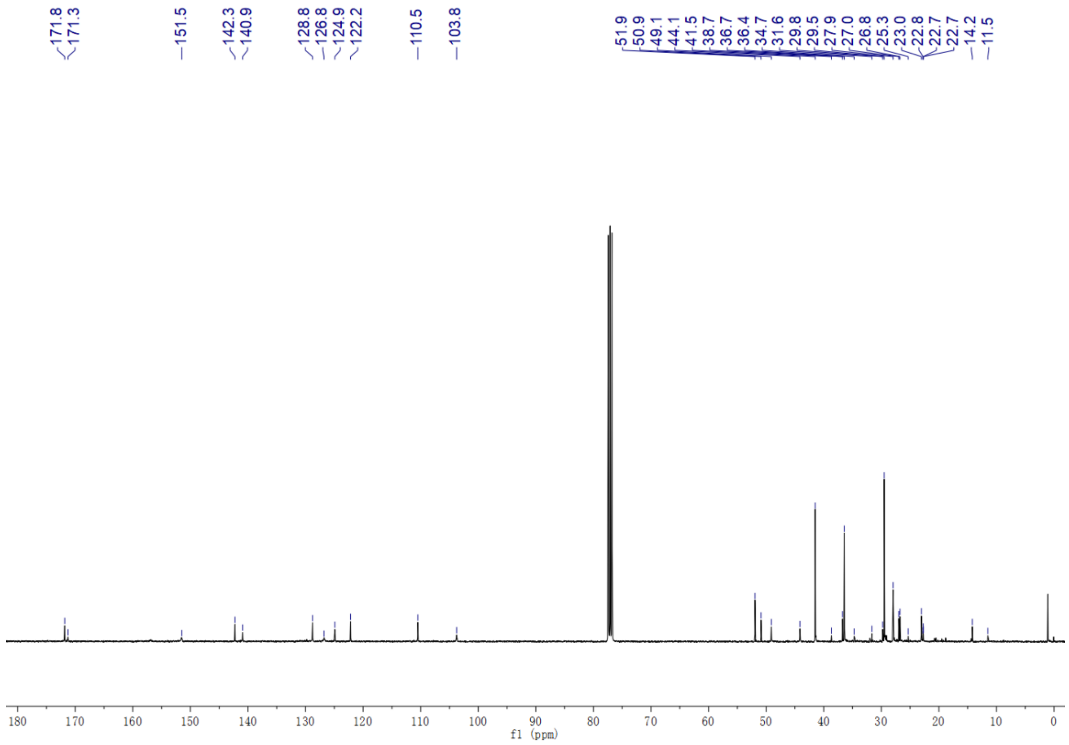

Fig. S69. ^{13}C NMR (101 MHz, Chloroform-*d*) spectrum of Cy7AA.

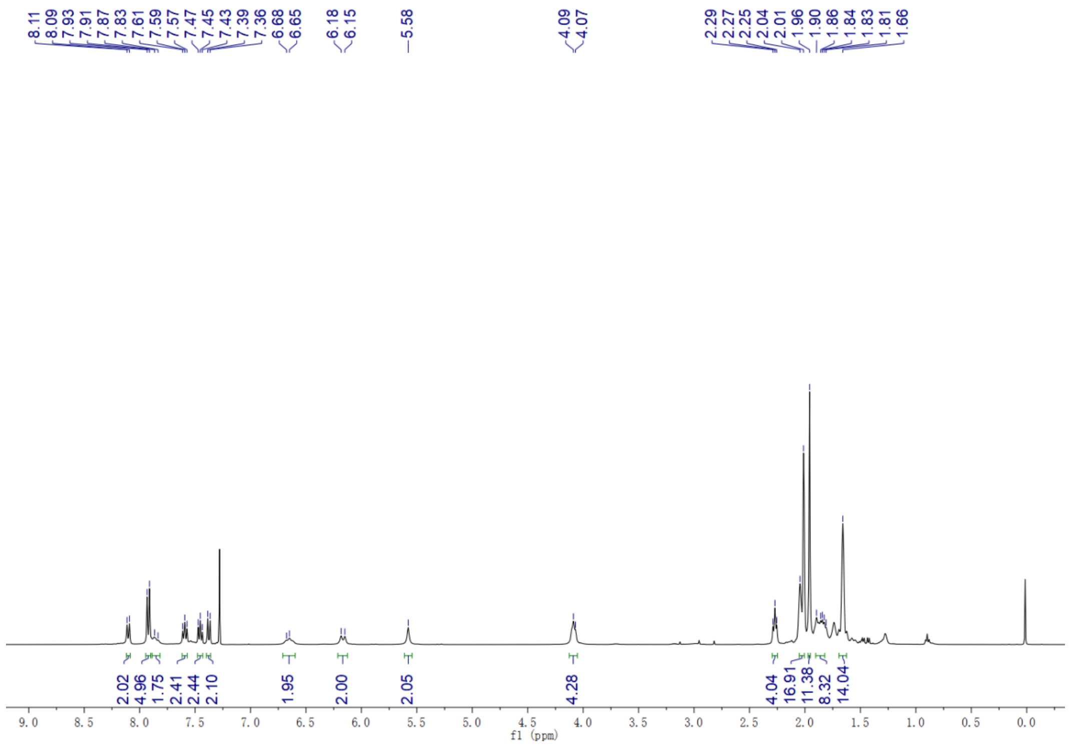

Fig. S70. ^1H NMR (400 MHz, Chloroform-*d*) spectrum of Cy7.5AA.

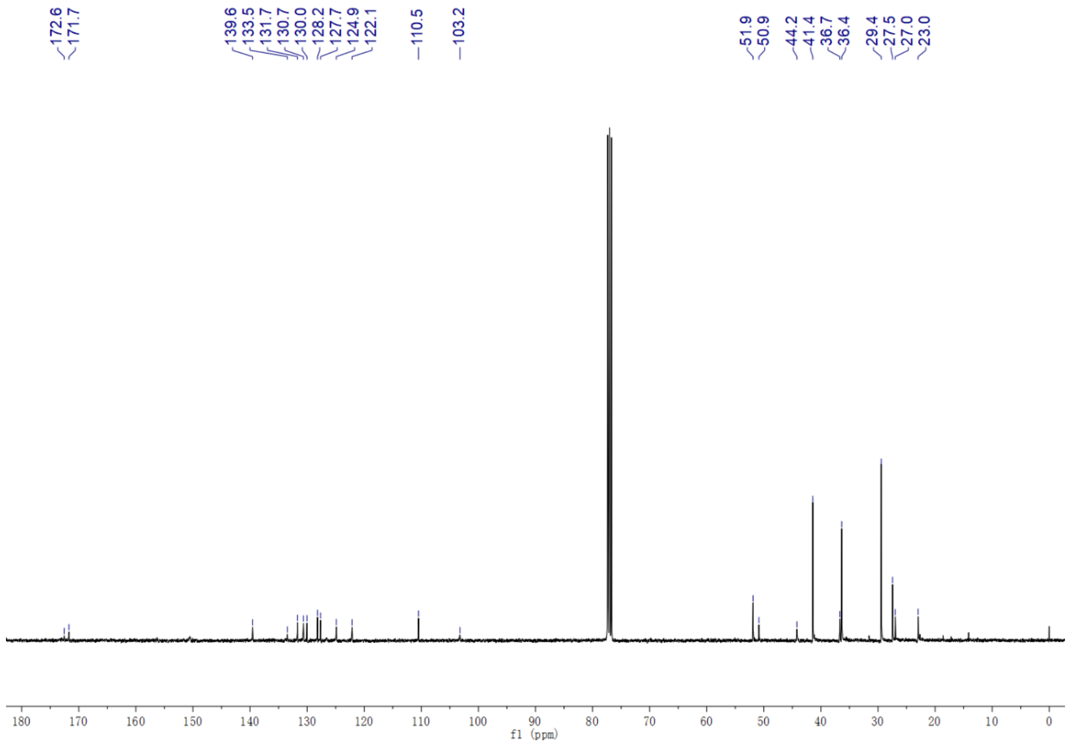

Fig. S71. ^{13}C NMR (101 MHz, Chloroform-*d*) spectrum of Cy7.5AA.

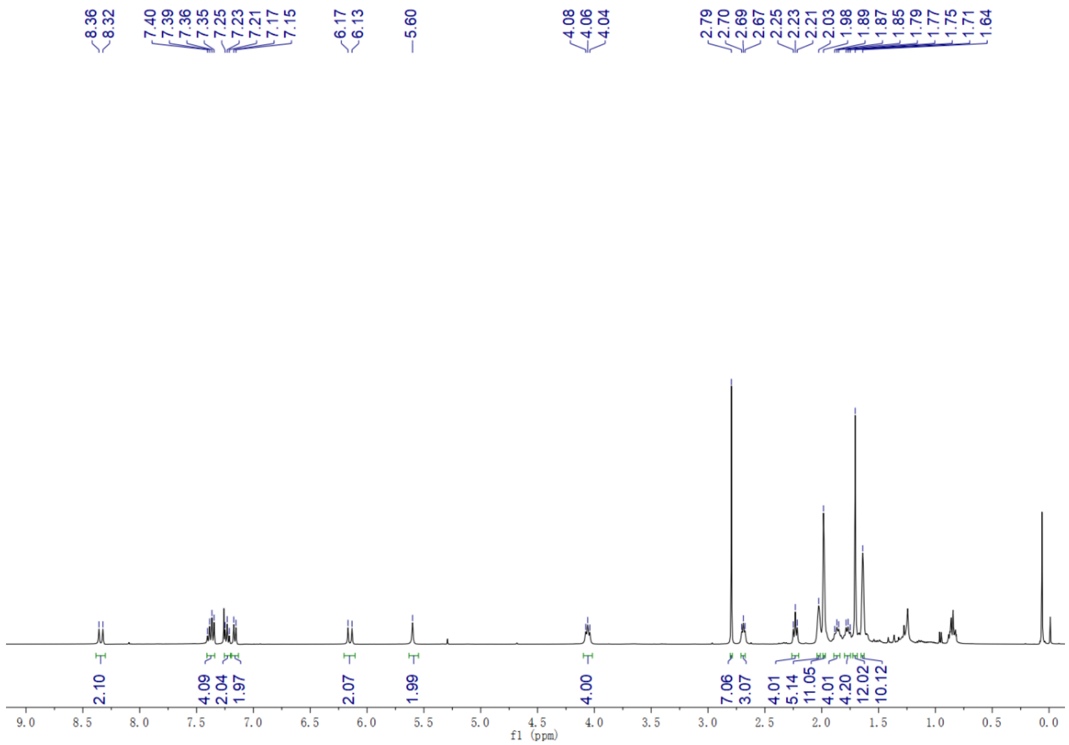

Fig. S72. ^{1}H NMR (400 MHz, Chloroform-*d*) spectrum of Cl-Cy7AA.

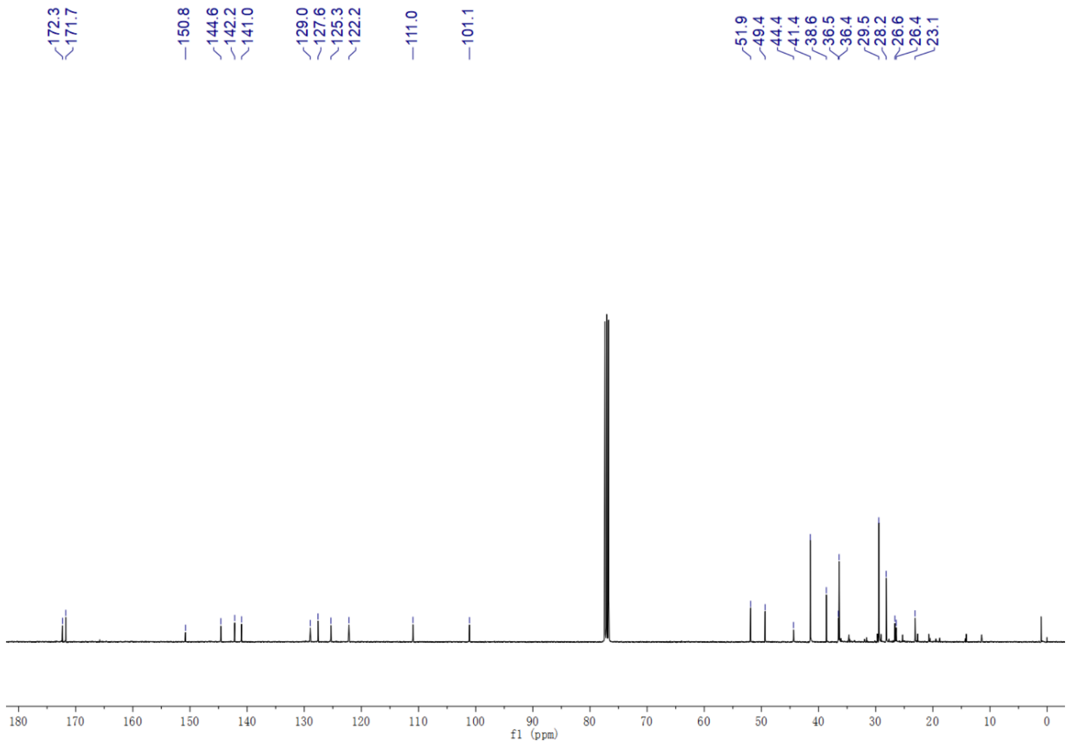

Fig. S73. ^{13}C NMR (101 MHz, Chloroform-*d*) spectrum of Cl-Cy7AA.

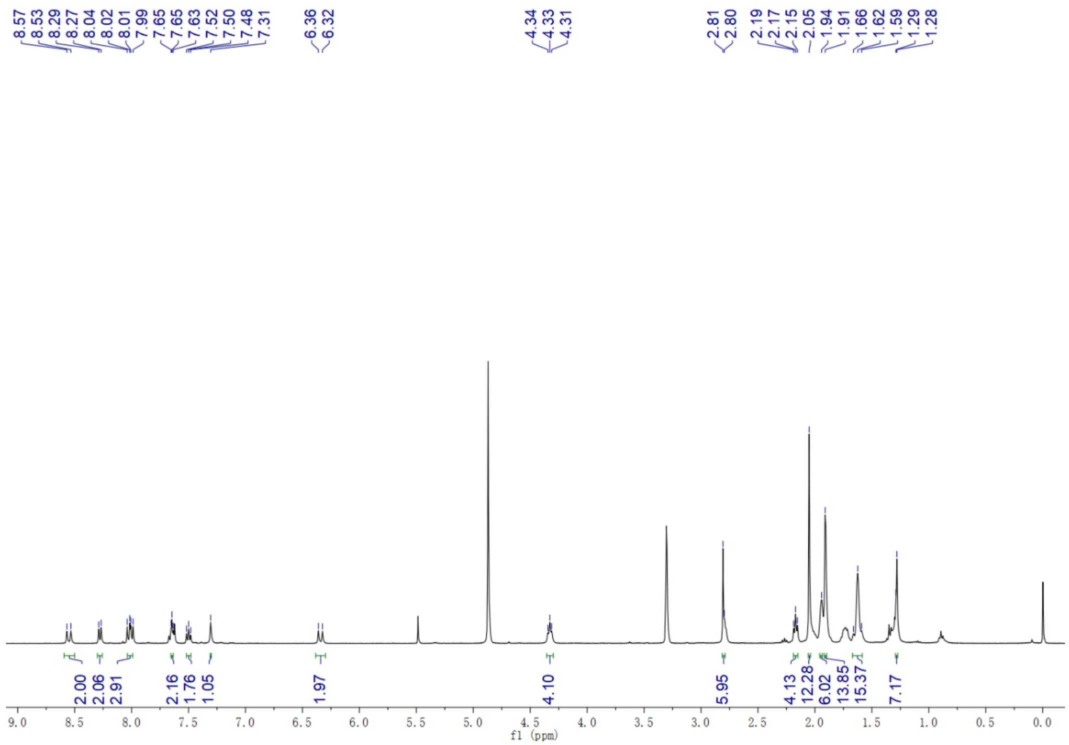

Fig. S74. ^{1}H NMR (400 MHz, Methanol-d_4) spectrum of Cl-Cy7.5AA.

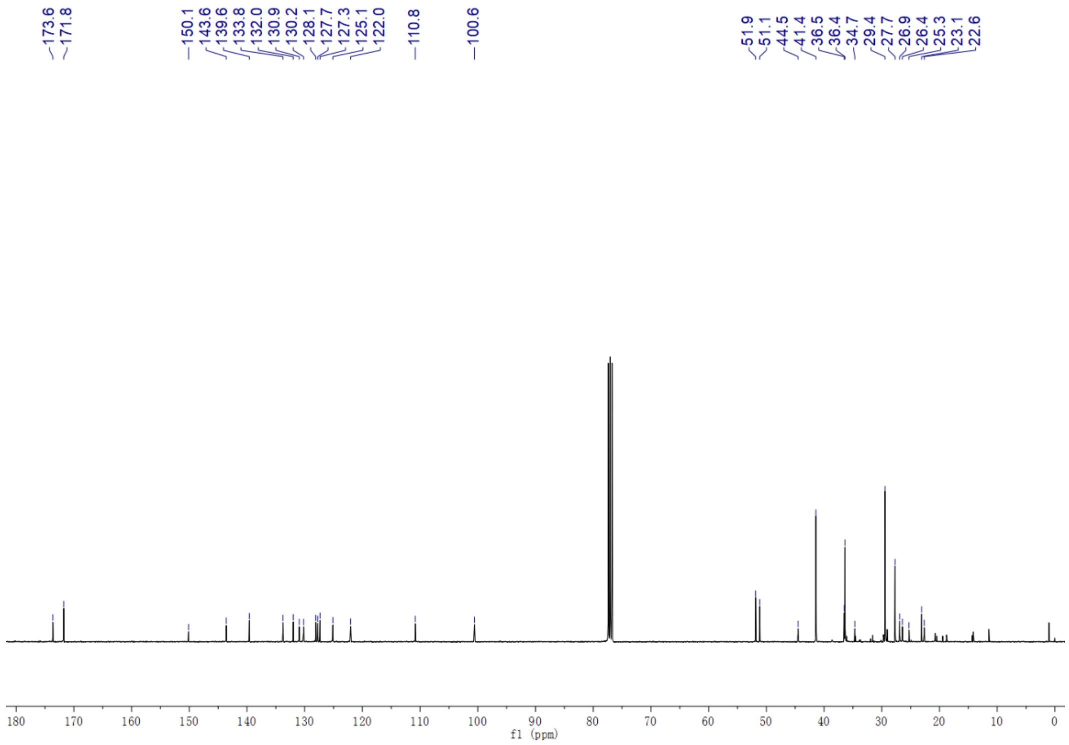

Fig. S75. ^{13}C NMR (101 MHz, Chloroform-d) spectrum of Cl-Cy7.5AA.

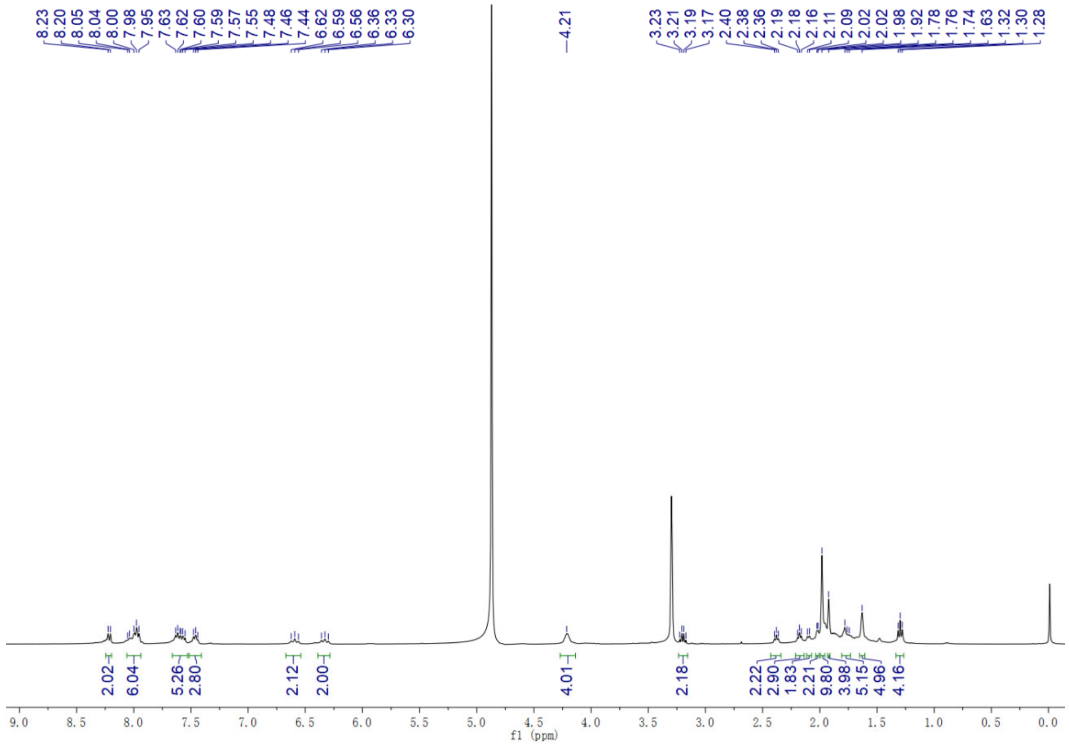

Fig. S76. ^{1}H NMR (400 MHz, Methanol-d_4) spectrum of Cy7.5AC.

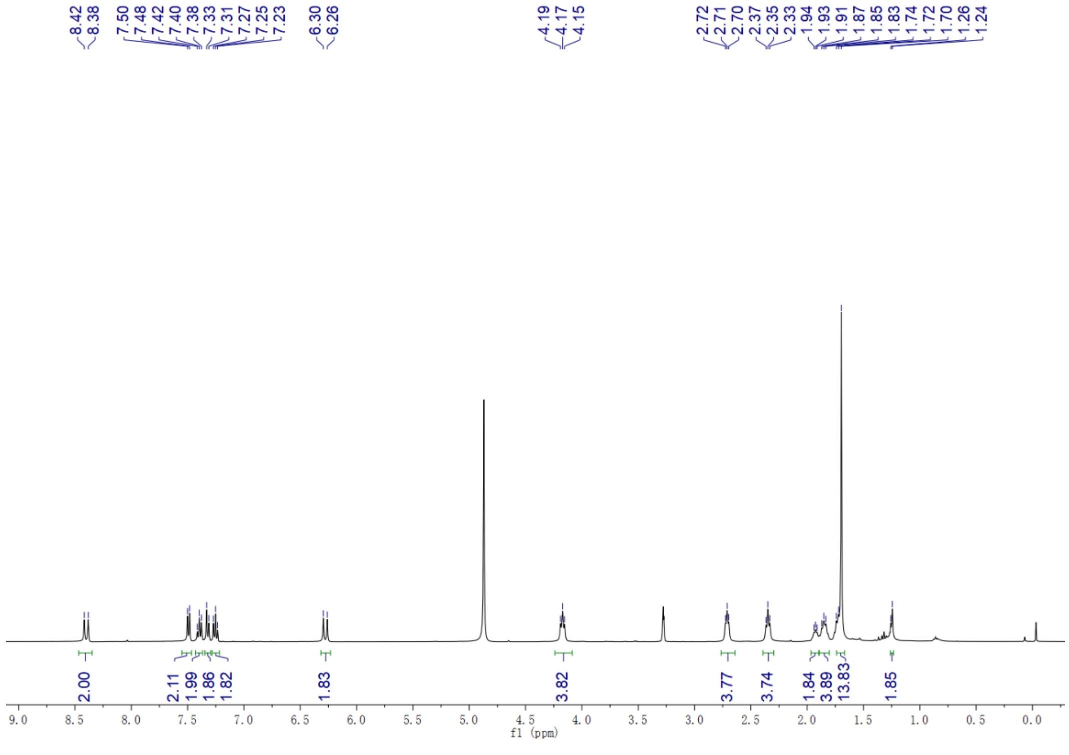

Fig. S77. ^{1}H NMR (400 MHz, MeOH) spectrum of Cl-Cy7COOH.

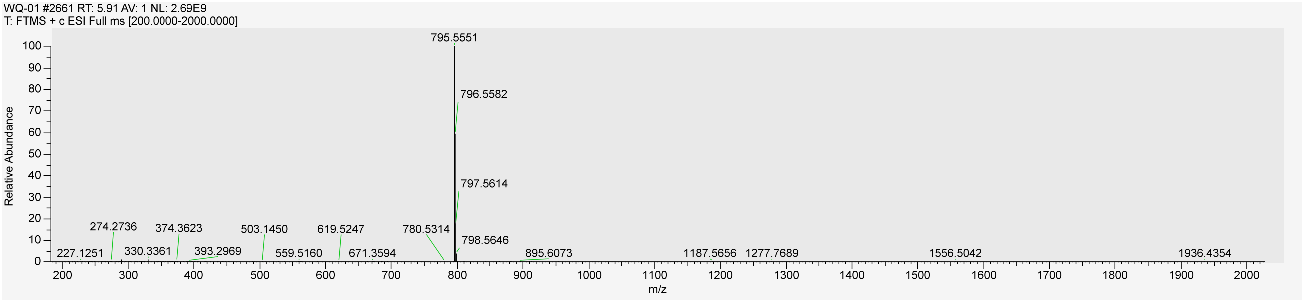

Fig. S78. HRMS spectrum of Cy3AA.

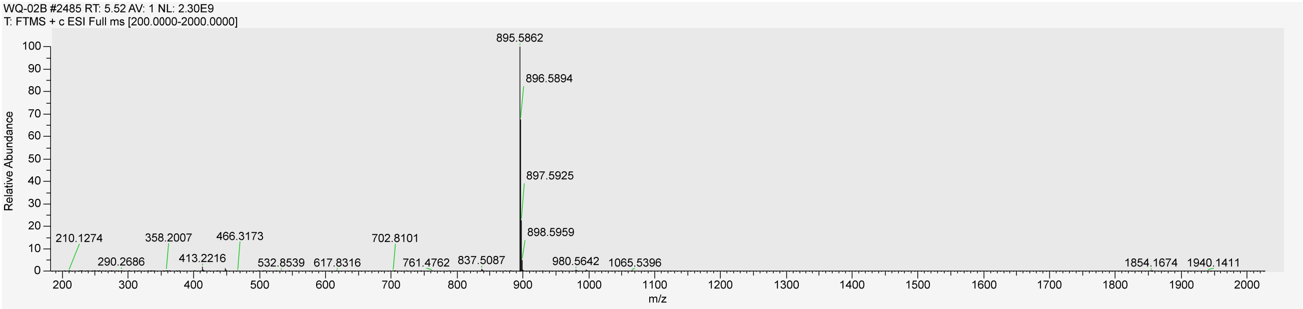

Fig. S79. HRMS spectrum of Cy3.5AA.

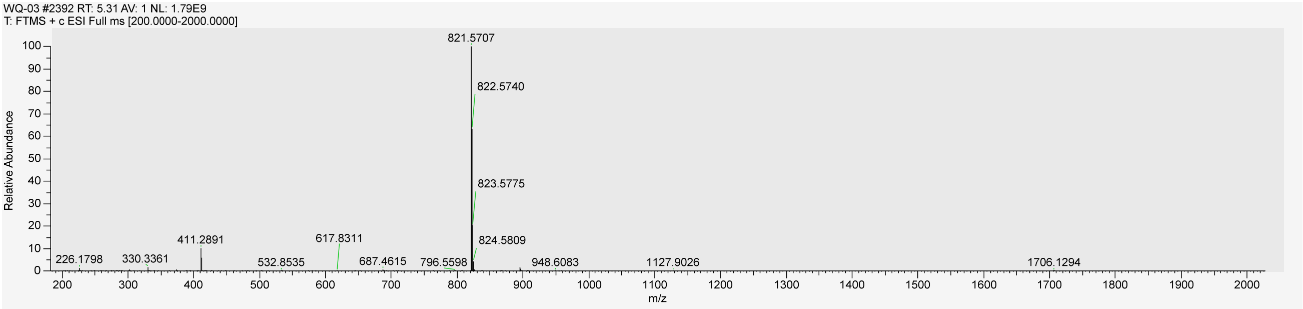

Fig. S80. HRMS spectrum of Cy5AA.

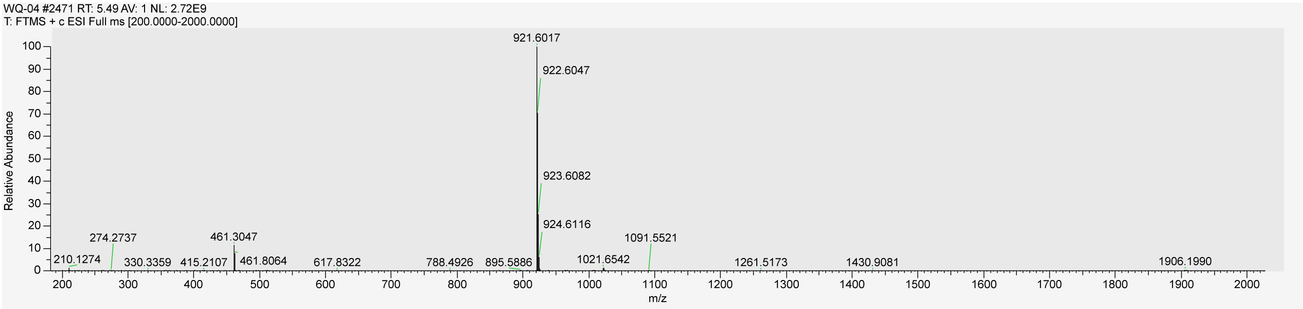

Fig. S81. HRMS spectrum of Cy5.5AA.

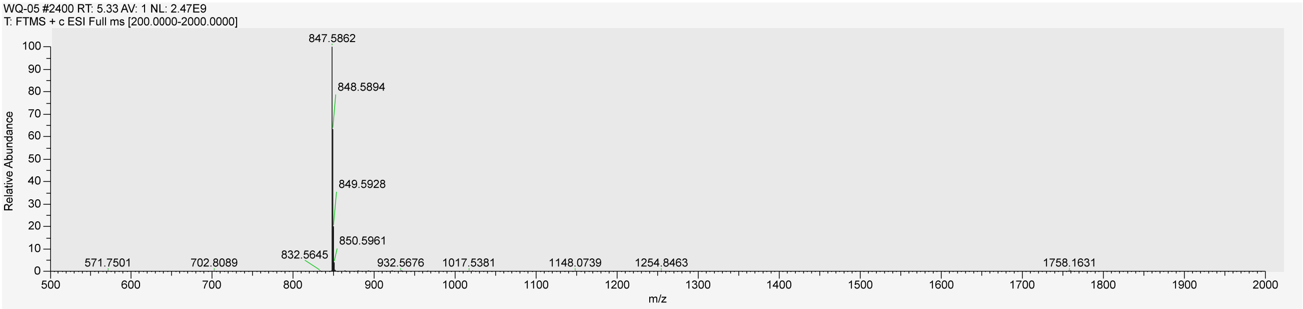

Fig. S82. HRMS spectrum of Cy7AA.

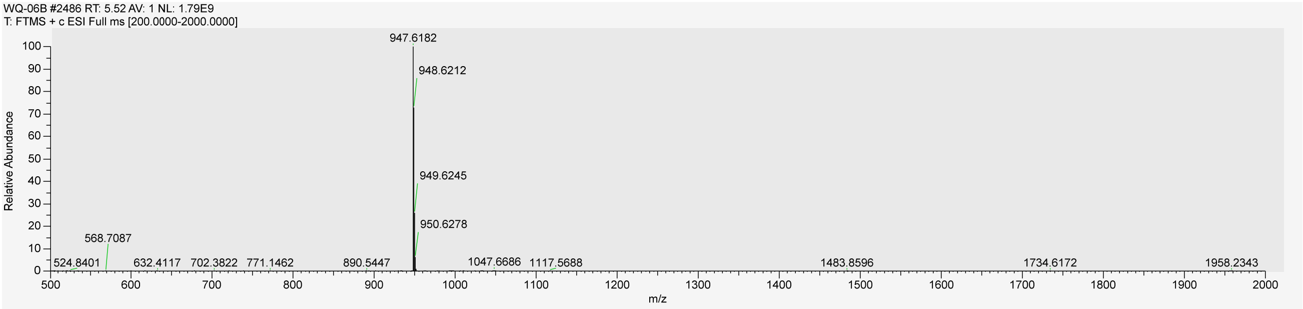

Fig. S83. HRMS spectrum of Cy7.5AA.

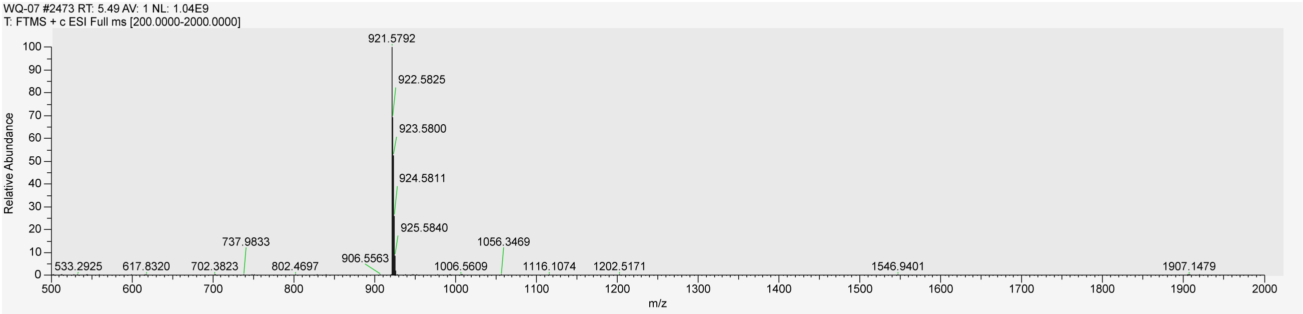

Fig. S84. Cl-Cy7AA.

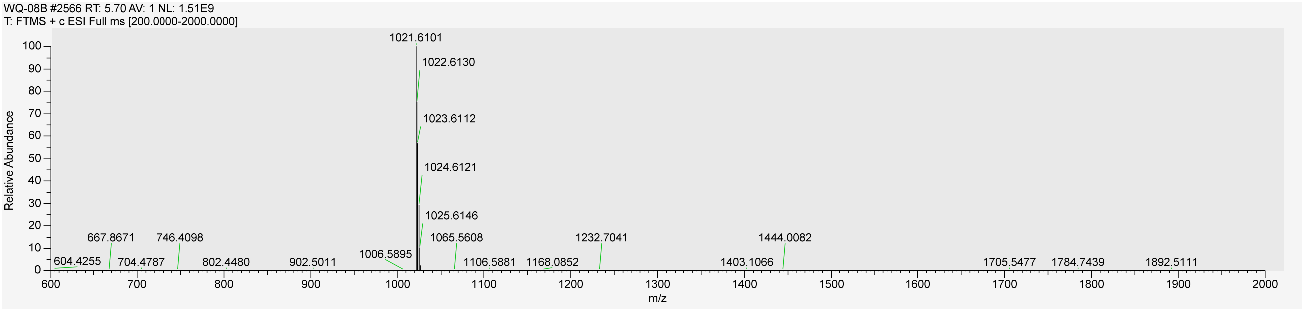

Fig. S85. HRMS spectrum of Cl-Cy7.5AA.

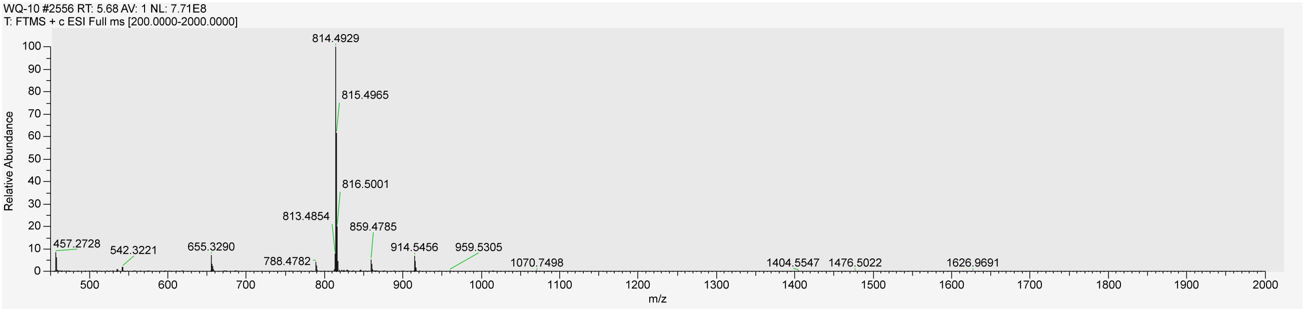

Fig. S86. HRMS spectrum of Cy7.5AC.

3. Supplementary Tables

Table S1. Apparent association constants (K_a) of different supramolecular probes with CDP as the host molecule in PBS (10 mM, pH 7.4).

K_a (M^{-1})	Cyn	Cyn.5	CynAA	Cyn.5AA
n = 3	$(7.06\pm0.88)x10^5$	$(2.38\pm0.21)x10^6$	$(1.99\pm0.11)\ x10^6$	$(2.15\pm0.56)\ x10^6$
n = 5	$(9.47\pm1.96)x10^5$	$(1.64\pm0.37)x10^6$	$(3.94\pm0.80)\ x10^6$	$(8.86\pm1.24)\ x10^6$
n = 7	$(2.80\pm0.30)x10^5$	$(9.12\pm0.95)x10^5$	$(1.40\pm0.20)\ x10^6$	$(5.38\pm0.89)\ x10^8$

Table S2. Apparent association constants (K_a) of a series of supramolecular probes with Cy7 skeleton in PBS (10 mM, pH 7.4).

Host \ Guest	Cy7	Cy7.5	Cy7AA	Cy7.5AC	Cy7.5AA
**CDP**	$(2.80\pm0.30)\times10^5$	$(9.12\pm0.95)\times10^5$	$(1.40\pm0.20)\times10^6$	$(4.21\pm0.40)\times10^6$	$(5.38\pm0.89)\times10^8$
**HPβCD**	$(1.97\pm0.91)\times10^5$	$(1.56\pm0.47)\times10^5$	$(7.45\pm0.87)\times10^5$	$(6.84\pm0.72)\times10^5$	$(1.80\pm0.70)\times10^6$

Table S3. Photophysical properties of different small molecule probes and supramolecular probes in PBS (10 mM, pH 7.4).

Fluorophore	λ_{ab} (nm)	λ_{em} (nm)	Stokes shift (nm)	ε ($\times 10^5$ $M^{-1}cm^{-1}$)	QY (φ,%)	$\varepsilon \times \varphi$ ($\times 10^5$ M^{-1} cm^{-1})
Cy3	544	565	21	1.6	5.1	8.2
Cy3AA@CDP	547	568	21	1.4	**57.3**	**80.2**
Cy3.5	635	642	7	0.4	7.2	2.8
Cy3.5AA@CDP	589	610	21	1.0	**74.0**	**74.0**
Cy5	640	665	25	1.8	16.3	29.3
Cy5AA@CDP	645	668	23	1.2	**58.8**	**70.6**
Cy5.5	676	701	25	0.5	9.0	4.5
Cy5.5AA@CDP	687	706	19	1.2	**60.2**	**72.2**
Cy7	741	772	31	0.9	8.9	8.1
Cy7AA@CDP	752	777	25	0.8	**14.1**	**11.3**
Cl-Cy7	774	797	23	0.4	8.9	0.8
Cl-Cy7AA@CDP	786	812	26	1.3	**10.2**	**13.3**
Cy7.5	700	—	—	0.2	6.6	1.3
ICG	778	817	39	0.7	2.0	1.5
Cy7.5AA@CDP	789	813	24	1.1	**15.5**	**17.1**

"-" represent that the value could not be measured, due to aggregation of Cy7.5.

Table S4. Calculation for renal clearance efficiency %ID of Cy7.5AA@CDP using absorbance

A	*c* (μM)	*x*	v_1 (mL)	**% ID**[a]
0.0073	0.259	20	1.40	**72.5**
0.024	1.162	5	1.20	**69.7**
0.0136	0.600	10	1.45	**87.0**

[a]The %ID (percentage of injected dose) can be calculated using the following formula:

$$\%\ \mathrm{ID} = \frac{\mathrm{c}\ x\ v_1}{c_0\ v_0} \times 100\%$$

Wherein *c* is the concentration of the solution calculated based on the absorbance *A* of the solution using the standard curve *A* = 0.0185*c* +0.0025, *x* is the dilution factor of the urine, v_1 is the volume of collected urine, c_0 is the concentration of the injected probe solution (100 μM), and v_0 is the volume of the injected solution (0.1 mL). The obtained parameters and calculation are shown in table S4. Thus, the renal clearance efficiency of Cy7.5AA@CDP was determined to be 76.4 ± 9.3% ID.

Table S5. Calculation for renal clearance efficiency %ID of ICG using absorbance

A	*c* (μM)	*x*	v_1 (mL)	**% ID[a]**
0.03204	0.367	1	1.40	**2.2**
0.03177	0.356	1	1.20	**1.1**
0.0359	0.515	1	1.45	**2.0**

[a]The %ID (percentage of injected dose) can be calculated using the following formula:

$$\%\,\mathrm{ID} = \frac{\mathrm{c}\;x\;v_1}{c_0\;v_0} \times 100\%$$

Wherein *c* is the concentration of the solution calculated based on the absorbance *A* of the solution using the standard curve $A = 0.026c + 0.0225$, *x* is the dilution factor of the urine, v_1 is the volume of collected urine, c_0 is the concentration of the injected probe solution (100 μM), and v_0 is the volume of the injected solution (0.1 mL). The obtained parameters and calculation are shown in table S5. Thus, the renal clearance efficiency of ICG was determined to be 1.8 ± 0.6% ID.

REFERENCES AND NOTES

1. H.-H. Han, H. Tian, Y. Zang, A. C. Sedgwick, J. Li, J. L. Sessler, X.-P. He, T. D. James, Small-molecule fluorescence-based probes for interrogating major organ diseases. *Chem. Soc. Rev.* **50**, 9391–9429 (2021).

2. G. Hong, A. L. Antaris, H. Dai, Near-infrared fluorophores for biomedical imaging. *Nat. Biomed. Eng.* **1**, 0010 (2017).

3. J. Huang, X. Chen, Y. Jiang, C. Zhang, S. He, H. Wang, K. Pu, Renal clearable polyfluorophore nanosensors for early diagnosis of cancer and allograft rejection. *Nat. Mater.* **21**, 598–607 (2022).

4. K. M. Tsoi, S. A. MacParland, X.-Z. Ma, V. N. Spetzler, J. Echeverri, B. Ouyang, S. M. Fadel, E. A. Sykes, N. Goldaracena, J. M. Kaths, J. B. Conneely, B. A. Alman, M. Selzner, M. A. Ostrowski, O. A. Adeyi, A. Zilman, I. D. McGilvray, W. C. W. Chan, Mechanism of hard-nanomaterial clearance by the liver. *Nat. Mater.* **15**, 1212–1221 (2016).

5. J. Shi, P. W. Kantoff, R. Wooster, O. C. Farokhzad, Cancer nanomedicine: Progress, challenges and opportunities. *Nat. Rev. Cancer* **17**, 20–37 (2017).

6. X. Jiang, B. Du, J. Zheng, Glutathione-mediated biotransformation in the liver modulates nanoparticle transport. *Nat. Nanotechnol.* **14**, 874–882 (2019).

7. J. Huang, J. Li, Y. Lyu, Q. Miao, K. Pu, Molecular optical imaging probes for early diagnosis of drug-induced acute kidney injury. *Nat. Mater.* **18**, 1133–1143 (2019).

8. H. S. Choi, S. L. Gibbs, J. H. Lee, S. H. Kim, Y. Ashitate, F. Liu, H. Hyun, G. Park, Y. Xie, S. Bae, M. Henary, J. V. Frangioni, Targeted zwitterionic near-infrared fluorophores for improved optical imaging. *Nat. Biotechnol.* **31**, 148–153 (2013).

9. M.-J. Baek, D.-T. Nguyen, D. Kim, S.-Y. Yoo, S. M. Lee, J.-Y. Lee, D.-D. Kim, Tailoring renal-clearable zwitterionic cyclodextrin for colorectal cancer-selective drug delivery. *Nat. Nanotechnol.* **18**, 945–956 (2023).

10. H. S. Choi, K. Nasr, S. Alyabyev, D. Feith, J. H. Lee, S. H. Kim, Y. Ashitate, H. Hyun, G. Patonay, L. Strekowski, M. Henary, J. V. Frangioni, Synthesis and in vivo fate of zwitterionic near-infrared fluorophores. *Angew. Chem. Int. Ed. Engl.* **50**, 6258–6263 (2011).

11. B. Du, Y. Chong, X. Jiang, M. Yu, U. G. Lo, A. Dang, Y.-A. Chen, S. Li, E. Hernandez, J. C. Lin, J.-T. Hsieh, J. Zheng, Hyperfluorescence imaging of kidney cancer enabled by renal secretion pathway dependent efflux transport. *Angew. Chem. Int. Ed. Engl.* **60**, 351–359 (2021).

12. Y. Chen, P. Pei, Z. Lei, X. Zhang, D. Yin, F. Zhang, A promising NIR-II fluorescent sensor for peptide-mediated long-term monitoring of kidney dysfunction. *Angew. Chem. Int. Ed. Engl.* **60**, 15809–15815 (2021).

13. P. Cheng, K. Pu, Molecular imaging and disease theranostics with renal-clearable optical agents. *Nat. Rev. Mater.* **6**, 1095–1113 (2021).

14. C. Yao, Y. Chen, M. Zhao, S. Wang, B. Wu, Y. Yang, D. Yin, P. Yu, H. Zhang, F. Zhang, A bright, renal-clearable NIR-II brush macromolecular probe with long blood circulation time for kidney disease bioimaging. *Angew. Chem. Int. Ed. Engl.* **61**, e202114273 (2022).

15. J. Zhou, L. Rao, G. Yu, T. R. Cook, X. Chen, F. Huang, Supramolecular cancer nanotheranostics. *Chem. Soc. Rev.* **50**, 2839–2891 (2021).

16. J. Zhao, Z. Zhou, G. Li, P. J. Stang, X. Yan, Light-emitting self-assembled metallacages. *Natl. Sci. Rev.* **8**, nwab045 (2021).

17. S. Chagri, D. Y. W. Ng, T. Weil, Designing bioresponsive nanomaterials for intracellular self-assembly. *Nat. Rev. Chem.* **6**, 320–338 (2022).

18. A. Levin, T. A. Hakala, L. Schnaider, G. J. L. Bernardes, E. Gazit, T. P. J. Knowles, Biomimetic peptide self-assembly for functional materials. *Nat. Rev. Chem.* **4**, 615–634 (2020).

19. R. M. Williams, J. Shah, B. D. Ng, D. R. Minton, L. J. Gudas, C. Y. Park, D. A. Heller, Mesoscale nanoparticles selectively target the renal proximal tubule epithelium. *Nano Lett.* **15**, 2358–2364 (2015).

20. Y. Chen, S. Wang, F. Zhang, Near-infrared luminescence high-contrast in vivo biomedical imaging. *Nat. Rev. Bioeng.* **1**, 60–78 (2023).

21. J.-S. Guo, J.-J. Li, Z.-H. Wang, Y. Liu, Y.-X. Yue, H.-B. Li, X.-H. Zhao, Y.-J. Sun, Y.-H. Ding, F. Ding, D.-S. Guo, L. Wang, Y. Chen, Dual hypoxia-responsive supramolecular complex for cancer target therapy. *Nat. Commun.* **14**, 5634 (2023).

22. C. Chen, X. Ni, H.-W. Tian, Q. Liu, D.-S. Guo, D. Ding, Calixarene-based supramolecular AIE dots with highly inhibited nonradiative decay and intersystem crossing for ultrasensitive fluorescence image-guided cancer surgery. *Angew. Chem. Int. Ed. Engl.* **59**, 10008–10012 (2020).

23. M. Raynal, P. Ballester, A. Vidal-Ferran, P. W. N. M. van Leeuwen, Supramolecular catalysis. Part 1: Non-covalent interactions as a tool for building and modifying homogeneous catalysts. *Chem. Soc. Rev.* **43**, 1660–1733 (2014).

24. C. Fasting, C. A. Schalley, M. Weber, O. Seitz, S. Hecht, B. Koksch, J. Dernedde, C. Graf, E.-W. Knapp, R. Haag, Multivalency as a chemical organization and action principle. *Angew. Chem. Int. Ed. Engl.* **51**, 10472–10498 (2012).

25. W. Sun, S. Guo, C. Hu, J. Fan, X. Peng, Recent development of chemosensors based on cyanine platforms. *Chem. Rev.* **116**, 7768–7817 (2016).

26. V. G. Bandi, M. P. Luciano, M. Saccomano, N. L. Patel, T. S. Bischof, J. G. P. Lingg, P. T. Tsrunchev, M. N. Nix, B. Ruehle, C. Sanders, L. Riffle, C. M. Robinson, S. Difilippantonio, J. D. Kalen, U. Resch-Genger, J. Ivanic, O. T. Bruns, M. J. Schnermann, Targeted multicolor in vivo imaging over 1,000 nm enabled by nonamethine cyanines. *Nat. Methods* **19**, 353–358 (2022).

27. K. Hirose, A practical guide for the determination of binding constants. *J. Incl. Phenom. Macrocycl. Chem.* **39**, 193–209 (2001).

28. C. Dalvit, I. Gmür, P. Rößler, A. D. Gossert, Affinity measurement of strong ligands with NMR spectroscopy: Limitations and ways to overcome them. *Prog. Nucl. Magn. Reson. Spectrosc.* **138-139**, 52–69 (2023).

29. D. Shetty, J. K. Khedkar, K. M. Park, K. Kim, Can we beat the biotin–avidin pair? Cucurbit[7]uril-based ultrahigh affinity host–guest complexes and their applications. *Chem. Soc. Rev.* **44**, 8747–8761 (2015).

30. S. C. Penchala, M. R. Miller, A. Pal, J. Dong, N. R. Madadi, J. Xie, H. Joo, J. Tsai, P. Batoon, V. Samoshin, A. Franz, T. Cox, J. Miles, W. K. Chan, M. S. Park, M. M. Alhamadsheh, A biomimetic approach for enhancing the in vivo half-life of peptides. *Nat. Chem. Biol.* **11**, 793–798 (2015).

31. P. Aggarwal, J. B. Hall, C. B. McLeland, M. A. Dobrovolskaia, S. E. McNeil, Nanoparticle interaction with plasma proteins as it relates to particle biodistribution, biocompatibility and therapeutic efficacy. *Adv. Drug Deliv. Rev.* **61**, 428–437 (2009).

32. R. Bilardo, F. Traldi, A. Vdovchenko, M. Resmini, Influence of surface chemistry and morphology of nanoparticles on protein corona formation. *Wiley Interdiscip. Rev. Nanomed. Nanobiotechnol.* **14**, e1788 (2022).

33. H. Shinohara, A. Tanaka, T. Kitai, N. Yanabu, T. Inomoto, S. Satoh, E. Hatano, Y. Yamaoka, K. Hirao, Direct measurement of hepatic indocyanine green clearance with near-infrared spectroscopy: Separate evaluation of uptake and removal. *Hepatology* **23**, 137–144 (1996).

34. X. Ma, Y. Zhao, Biomedical applications of supramolecular systems based on host–guest interactions. *Chem. Rev.* **115**, 7794–7839 (2015).

35. J. Huang, C. Xie, X. Zhang, Y. Jiang, J. Li, Q. Fan, K. Pu, Renal-clearable molecular semiconductor for second near-infrared fluorescence imaging of kidney dysfunction. *Angew. Chem. Int. Ed. Engl.* **58**, 15120–15127 (2019).

36. B. Du, X. Jiang, A. Das, Q. Zhou, M. Yu, R. Jin, J. Zheng, Glomerular barrier behaves as an atomically precise bandpass filter in a sub-nanometre regime. *Nat. Nanotechnol.* **12**, 1096–1102 (2017).

37. B. Du, M. Yu, J. Zheng, Transport and interactions of nanoparticles in the kidneys. *Nat. Rev. Mater.* **3**, 358–374 (2018).

38. R. M. Williams, J. Shah, H. S. Tian, X. Chen, F. Geissmann, E. A. Jaimes, D. A. Heller, Selective nanoparticle targeting of the renal tubules. *Hypertension* **71**, 87–94 (2018).

39. W. Lee, R. B. Kim, Transporters and renal drug elimination. *Annu. Rev. Pharmacol. Toxicol.* **44**, 137–166 (2004).

40. B. Wang, X. He, Z. Zhang, Y. Zhao, W. Feng, Metabolism of nanomaterials in vivo: Blood circulation and organ clearance. *Acc. Chem. Res.* **46**, 761–769 (2013).

41. L. Ye, K.-T. Yong, L. Liu, I. Roy, R. Hu, J. Zhu, H. Cai, W.-C. Law, J. Liu, K. Wang, J. Liu, Y. Liu, Y. Hu, X. Zhang, M. T. Swihart, P. N. Prasad, A pilot study in non-human primates shows no adverse response to intravenous injection of quantum dots. *Nat. Nanotechnol.* **7**, 453–458 (2012).

42. J. A. Kellum, P. Romagnani, G. Ashuntantang, C. Ronco, A. Zarbock, H.-J. Anders, Acute kidney injury. *Nat. Rev. Dis. Primers.* **7**, 52 (2021).

43. J. A. Kellum, J. R. Prowle, Paradigms of acute kidney injury in the intensive care setting. *Nat. Rev. Nephrol.* **14**, 217–230 (2018).

44. M. Joannidis, W. Druml, L. G. Forni, A. B. J. Groeneveld, P. M. Honore, E. Hoste, M. Ostermann, H. M. Oudemans-van Straaten, M. Schetz, Prevention of acute kidney injury and protection of renal function in the intensive care unit: Update 2017. *Intensive Care Med.* **43**, 730–749 (2017).

45. M. Darmon, M. Ostermann, J. Cerda, M. A. Dimopoulos, L. Forni, E. Hoste, M. Legrand, N. Lerolle, E. Rondeau, A. Schneider, B. Souweine, M. Schetz, Diagnostic work-up and specific causes of acute kidney injury. *Intensive Care Med.* **43**, 829–840 (2017).

46. Y. Xu, H. Ma, J. Shao, J. Wu, L. Zhou, Z. Zhang, Y. Wang, Z. Huang, J. Ren, S. Liu, X. Chen, J. Han, A role for tubular necroptosis in cisplatin-induced AKI. *J. Am. Soc. Nephrol.* **26**, 2647–2658 (2015).

47. C. Tang, M. J. Livingston, R. Safirstein, Z. Dong, Cisplatin nephrotoxicity: New insights and therapeutic implications. *Nat. Rev. Nephrol.* **19**, 53–72 (2023).

48. A. Ozkok, C. L. Edelstein, Pathophysiology of cisplatin-induced acute kidney injury. *Biomed. Res. Int.* **2014**, 967826 (2014).

49. J. Weng, Y. Wang, Y. Zhang, D. Ye, An activatable near-infrared fluorescence probe for in vivo imaging of acute kidney injury by targeting phosphatidylserine and caspase-3. *J. Am. Chem. Soc.* **143**, 18294–18304 (2021).

50. D. Jiang, Z. Ge, H.-J. Im, C. G. England, D. Ni, J. Hou, L. Zhang, C. J. Kutyreff, Y. Yan, Y. Liu, S. Y. Cho, J. W. Engle, J. Shi, P. Huang, C. Fan, H. Yan, W. Cai, DNA origami nanostructures can exhibit preferential renal uptake and alleviate acute kidney injury. *Nat. Biomed. Eng.* **2**, 865–877 (2018).

51. P. Cheng, W. Chen, S. Li, S. He, Q. Miao, K. Pu, Fluoro-photoacoustic polymeric renal reporter for real-time dual imaging of acute kidney injury. *Adv. Mater.* **32**, 1908530 (2020).

52. J. Huang, Y. Lyu, J. Li, P. Cheng, Y. Jiang, K. Pu, A renal-clearable duplex optical reporter for real-time imaging of contrast-induced acute kidney injury. *Angew. Chem. Int. Ed. Engl.* **58**, 17796–17804 (2019).

53. M. Yu, J. Zhou, B. Du, X. Ning, C. Authement, L. Gandee, P. Kapur, J.-T. Hsieh, J. Zheng, Noninvasive staging of kidney dysfunction enabled by renal-clearable luminescent gold nanoparticles. *Angew. Chem. Int. Ed. Engl.* **55**, 2787–2791 (2016).

54. R. L. Siegel, K. D. Miller, A. Jemal, Cancer statistics, 2020. *CA Cancer J. Clin.* **70**, 7–30 (2020).

55. Z. Hu, C. Fang, B. Li, Z. Zhang, C. Cao, M. Cai, S. Su, X. Sun, X. Shi, C. Li, T. Zhou, Y. Zhang, C. Chi, P. He, X. Xia, Y. Chen, S. S. Gambhir, Z. Cheng, J. Tian, First-in-human

liver-tumour surgery guided by multispectral fluorescence imaging in the visible and near-infrared-I/II windows. *Nat. Biomed. Eng.* **4**, 259–271 (2020).

56. H. Wang, X. Li, B. W.-C. Tse, H. Yang, C. A. Thorling, Y. Liu, M. Touraud, J. B. Chouane, X. Liu, M. S. Roberts, X. Liang, Indocyanine green-incorporating nanoparticles for cancer theranostics. *Theranostics* **8**, 1227–1242 (2018).

57. L. van Manen, H. J. M. Handgraaf, M. Diana, J. Dijkstra, T. Ishizawa, A. L. Vahrmeijer, J. S. D. Mieog, A practical guide for the use of indocyanine green and methylene blue in fluorescence-guided abdominal surgery. *J. Surg. Oncol.* **118**, 283–300 (2018).

58. A. L. Antaris, H. Chen, K. Cheng, Y. Sun, G. Hong, C. Qu, S. Diao, Z. Deng, X. Hu, B. Zhang, X. Zhang, O. K. Yaghi, Z. R. Alamparambil, X. Hong, Z. Cheng, H. Dai, A small-molecule dye for NIR-II imaging. *Nat. Mater.* **15**, 235–242 (2016).

59. E. D. Cosco, A. L. Spearman, S. Ramakrishnan, J. G. P. Lingg, M. Saccomano, M. Pengshung, B. A. Arús, K. C. Y. Wong, S. Glasl, V. Ntziachristos, M. Warmer, R. R. McLaughlin, O. T. Bruns, E. M. Sletten, Shortwave infrared polymethine fluorophores matched to excitation lasers enable non-invasive, multicolour in vivo imaging in real time. *Nat. Chem.* **12**, 1123–1130 (2020).

60. J. A. Carr, D. Franke, J. R. Caram, C. F. Perkinson, M. Saif, V. Askoxylakis, M. Datta, D. Fukumura, R. K. Jain, M. G. Bawendi, O. T. Bruns, Shortwave infrared fluorescence imaging with the clinically approved near-infrared dye indocyanine green. *Proc. Natl. Acad. Sci. U.S.A.* **115**, 4465–4470 (2018).

61. T. Kitai, T. Inomoto, M. Miwa, T. Shikayama, Fluorescence navigation with indocyanine green for detecting sentinel lymph nodes in breast cancer. *Breast Cancer* **12**, 211–215 (2005).

62. M. G. Jørgensen, A. P. Hermann, A. R. Madsen, S. Christensen, J. A. Sørensen, Indocyanine green lymphangiography is superior to clinical staging in breast cancer-related lymphedema. *Sci. Rep.* **11**, 21103 (2021).

63. B. Ouyang, W. Poon, Y.-N. Zhang, Z. P. Lin, B. R. Kingston, A. J. Tavares, Y. Zhang, J. Chen, M. S. Valic, A. M. Syed, P. MacMillan, J. Couture-Senécal, G. Zheng, W. C. W. Chan, The dose threshold for nanoparticle tumour delivery. *Nat. Mater.* **19**, 1362–1371 (2020).

64. Q. Wen, Y. Zhang, C. Li, S. Ling, X. Yang, G. Chen, Y. Yang, Q. Wang, NIR-II fluorescent self-assembled peptide nanochain for ultrasensitive detection of peritoneal metastasis. *Angew. Chem. Int. Ed. Engl.* **58**, 11001–11006 (2019).

65. D. Zhong, W. Chen, Z. Xia, R. Hu, Y. Qi, B. Zhou, W. Li, J. He, Z. Wang, Z. Zhao, D. Ding, M. Tian, B. Z. Tang, M. Zhou, Aggregation-induced emission luminogens for image-guided surgery in non-human primates. *Nat. Commun.* **12**, 6485 (2021).

66. P. Wang, Y. Fan, L. Lu, L. Liu, L. Fan, M. Zhao, Y. Xie, C. Xu, F. Zhang, NIR-II nanoprobes in-vivo assembly to improve image-guided surgery for metastatic ovarian cancer. *Nat. Commun.* **9**, 2898 (2018).

67. D. M. Mofford, G. R. Reddy, S. C. Miller, Aminoluciferins extend firefly luciferase bioluminescence into the near-infrared and can be preferred substrates over D-luciferin. *J. Am. Chem. Soc.* **136**, 13277–13282 (2014).

68. H. Langhals, A. Varja, P. Laubichler, M. Kernt, K. Eibl, C. Haritoglou, Cyanine dyes as optical contrast agents for ophthalmological surgery. *J. Med. Chem.* **54**, 3903–3925 (2011).

69. S. M. Usama, K. Burgess, Hows and whys of tumor-seeking dyes. *Acc. Chem. Res.* **54**, 2121–2131 (2021).

70. S. M. Usama, C.-M. Lin, K. Burgess, On the mechanisms of uptake of tumor-seeking cyanine dyes. *Bioconjug. Chem.* **29**, 3886–3895 (2018).

71. P. Kumar, A. Nagarajan, P. D. Uchil, Analysis of cell viability by the MTT assay. *Cold Spring Harb. Protoc.* **2018**, pdb.prot095505 (2018).

72. B. Li, L. Lu, M. Zhao, Z. Lei, F. Zhang, An efficient 1064 nm NIR-II excitation fluorescent molecular dye for deep-tissue high-resolution dynamic bioimaging. *Angew. Chem. Int. Ed. Engl.* **57**, 7483–7487 (2018).

73. T.-B. Ren, Z.-Y. Wang, Z. Xiang, P. Lu, H.-H. Lai, L. Yuan, X.-B. Zhang, W. Tan, A general strategy for development of activatable NIR-II fluorescent probes for in vivo high-contrast bioimaging. *Angew. Chem. Int. Ed. Engl.* **60**, 800–805 (2021).

74. M. Matsui, S. Ando, M. Fukushima, T. Shibata, Y. Kubota, K. Funabiki, Fluorescence properties of indolenium carbocyanine dyes in solid state. *Tetrahedron* **71**, 3528–3534 (2015).

75. E. A. Owens, H. Hyun, J. G. Tawney, H. S. Choi, M. Henary, Correlating molecular character of NIR imaging agents with tissue-specific uptake. *J. Med. Chem.* **58**, 4348–4356 (2015).

76. A. Samanta, M. Vendrell, R. Das, Y.-T. Chang, Development of photostable near-infrared cyanine dyes. *Chem. Commun.* **46**, 7406–7408 (2010).

77. C. L. Lawrence, A. O. Okoh, V. Vishwapathi, S. T. McKenna, M. E. Critchley, R. B. Smith, N-alkylated linear heptamethine polyenes as potent non-azole leads against Candida albicans fungal infections. *Bioorg. Chem.* **102**, 104070 (2020).

78. E. A. Owens, N. Bruschi, J. G. Tawney, M. Henary, A microwave-assisted and environmentally benign approach to the synthesis of near-infrared fluorescent pentamethine cyanine dyes. *Dyes Pigm.* **113**, 27–37 (2015).

79. J. Peng, A. Samanta, X. Zeng, S. Han, L. Wang, D. Su, D. T. B. Loong, N.-Y. Kang, S.-J. Park, A. H. All, W. Jiang, L. Yuan, X. Liu, Y.-T. Chang, Real-time in vivo hepatotoxicity monitoring through chromophore-conjugated photon-upconverting nanoprobes. *Angew. Chem. Int. Ed. Engl.* **56**, 4165–4169 (2017).
